# Membrane protein solubilization and structure determination using de novo–designed proteins

**DOI:** 10.1126/science.adr3817

**Published:** 2026-07-02

**Authors:** Ljubica Mihaljević, David E. Kim, Pooja D. Bandawane, Helen E. Eisenach, Andrew J. Borst, Alexis Courbet, Connor Weidle, Kenneth D. Carr, Everton Bettin, Qiushi Liu, Aldo T. Trejos, Sagardip Majumder, Surabhi Kokane, Alexander Stevens, Edin Muratspahić, Thomas Schlichthaerle, Marc Expòsit, Xinting Li, Mila Lamb, Analisa Nicole Azcárraga Murray, Rashmi Ravichandran, Elizabeth C. Williams, Shuyuan Hu, Lynda Stuart, Linda Grillová, Nicholas R. Thomson, Michael Landreh, Pengxiang Chang, Lorenzo Giacani, Melissa J. Caimano, Kelly L. Hawley, Neil P. King, David Baker

**Affiliations:** 1 Department of Biochemistry, Institute for Protein Design, University of Washington, Seattle, WA 98195, USA.; 2 Department of Pediatrics, UConn Health, Farmington, CT 06030-3715, USA.; 3 Department of Medicine, UConn Health, Farmington, CT 06030-3715, USA.; 4 Department of Molecular Biology and Biophysics, UConn Health, Farmington, CT 06030-3715, USA.; 5 Division of Infectious Diseases and Immunology, Connecticut Children’s, Hartford CT 06106, USA.; 6 Department of Research, Connecticut Children’s Research Institute, Hartford CT 06106, USA.; 7 Howard Hughes Medical Institute, University of Washington, Seattle, WA, USA.; 8 Centre de Biologie Structurale, University of Montpellier, CNRS, INSERM. Montpellier, France.; 9 Kymab, a Sanofi Company, Babraham Research Campus, Cambridge, UK.; 10 Parasites and Microbes Programme, Wellcome Sanger Institute, Hinxton, UK.; 11 Faculty of Infectious and Tropical Diseases, London School of Hygiene & Tropical Medicine, London, UK.; 12 Department of Medicine, Division of Allergy and Infectious Diseases, University of Washington; 13 Department of Global Health, University of Washington; 14 Department of Cell and Molecular Biology, Uppsala University, 751 24 Uppsala, Sweden; 15 Department of Microbiology, Tumor and Cell Biology, Karolinska Institutet, 171 65 Solna, Sweden

## Abstract

Developing therapies and vaccines against integral membrane proteins is hindered by their extensive hydrophobic surfaces, which complicate production and structural analysis. Here, we describe a general deep learning–based design approach for solubilizing native membrane proteins while preserving their sequence, fold, active-site, and ligand-binding properties. Genetically encoded *de novo* protein WRAPs [water-soluble RFdiffused amphipathic proteins] surround the lipid-interacting hydrophobic surfaces, rendering them thermostable and water-soluble without the need for detergents. We design WRAPs for both monomeric and oligomeric beta-barrel outer membrane proteins and helical multipass transmembrane proteins. A 2.95-angstrom-resolution cryo–electron microscopy structure of WRAPed mycobacterial porin demonstrates that WRAPs can be used for the structural determination of membrane proteins in solution. As a step toward syphilis vaccine development, we generated soluble versions of *Treponema pallidum* antigens.

Membrane proteins constitute nearly a third of the proteome (1), are targeted by more than half of clinically approved drugs (1, 2), and have considerable potential as vaccine targets (3). However, the challenges associated with working with membrane proteins, particularly their extraction and poor stability (4), remain substantial barriers to research and drug discovery efforts. Redesigning membrane proteins to be expressed in a soluble form bypasses the need for conventional extraction from the membrane with detergents (5–8). Most existing redesign methods rely on modifying native protein sequences by mutating lipid-facing hydrophobic residues to polar amino acids either through manual manipulation (5) or computational approaches (6–8). The SIMPLEx (solubilization of integral membrane proteins with high levels of expression) method uses genetic fusion of the amphipathic apolipoprotein AI (Apo-AI) and a soluble decoy to membrane proteins, conferring water solubility without modification of the native sequence by shielding the lipid-facing hydrophobic regions through nonspecific hydrophobic interactions (9). However, the flexibility of Apo-AI and the lack of specificity of the interactions with the shielded target likely complicate structure determination (9).

We reasoned that machine learning–based tools for de novo protein design could enable the design of custom solubilizing domains with a polar exterior and a nonpolar interior complementary to the lipid-facing hydrophobic surface of membrane proteins of interest. We set out to explore the use of RoseTTAFold diffusion (RFdiffusion), a generative model for de novo protein backbone design (10), to generate both alpha-helical and beta-sheet target-specific solubilizing domains, which we named WRAPs (water-soluble RFdiffused amphipathic proteins), and to investigate the structural and biochemical properties of the WRAPed targets.

## Results

### *De novo* design of WRAPs

In initial experiments, we found it difficult to guide free diffusion (starting from randomized coordinates) of the terminus of a target membrane protein to fully protect all of the lipid-facing hydrophobic regions. Therefore, we instead turned to a two-step approach in which we first generated idealized de novo helical and beta-barrel backbones for WRAPs that roughly matched the diameter and height of the membrane-spanning portion of the target protein. Cylindrical antiparallel helical assemblies with an inner diameter compatible with the membrane-spanning region of the target protein were generated using fold-conditioned RFdiffusion (10) (Fig. 1A and fig. S1A). To generate beta-barrel backbones, we built ideal backbones parametrically (11), selecting the length and number of beta strands and the barrel shear (the register shift between the first and last strands) to generate backbones with internal diameters and barrel heights to accommodate side-chain–packing interactions with the target protein while maintaining the interstrand hydrogen bonds of the barrel.

We next refined the designed backbones to be complementary in shape and side-chain interactions to the target protein using partial diffusion (12). The target protein was positioned in the center of the designed helical or barrel WRAP backbones using PyMOL (13) or PyRosetta (14) (Fig. 1A). Partial diffusion was then used to mold the de novo backbone (s) to the target by partially randomizing the coordinates of the designed backbones and successively removing noise using RFdiffusion conditioned on the target structure (Fig. 1B). With this conditioning, the resultant optimized backbones are expected to be as complementary in shape to the target as typical protein-protein interfaces in the Protein Data Bank (PDB). We then designed amino acid sequences for the de novo WRAP domains using a variant of ProteinMPNN (8, 15). Standard ProteinMPNN (15) was trained on a set of PDB structures that included transmembrane proteins and, as a result, it tends to place nonpolar residues on the exposed surface of long alpha helices. A version of ProteinMPNN specialized for soluble proteins (SolubleMPNN) (8) was therefore used to design sequences for the refined backbones, keeping the amino acid identities but not the side-chain conformations of the target membrane protein residues fixed, yielding sequences that direct folding to the desired structure and make specific interactions with the target. AlphaFold2 (AF2) (16) was then used to select designs predicted to fold to the target structure for experimental characterization. Per-residue confidence score (pLDDT_binder) and predicted aligned error of interaction (PAE_i) cutoffs for each design are outlined in table S1.

### Solubilization of outer membrane beta-barrel proteins

Outer membrane proteins (OMPs) are transmembrane beta-barrel proteins found in Gram-negative bacteria that upon recombinant expression require refolding from inclusion bodies in addition to purification with detergents (17). As a first test of our method, we sought to solubilize the outer membrane protein A (OmpA) of *Escherichia coli*, a well-characterized eight-stranded transmembrane beta-barrel protein (18). We generated both alpha-helical and beta-barrel WRAPs for OmpA [PDB ID: 2GE4(19)] and expressed the WRAP-OmpA as single-gene fusion proteins in the *E. coli* cytoplasm (fig. S1B). We were able to purify both helical (Fig. 2A) and beta-barrel (Fig. 2B) WRAP-OmpA proteins from the soluble fraction of *E. coli* lysates. Five of six N-terminally fused helical WRAP-OmpA designs and one of six beta-barrel WRAP-OmpA C-terminally fused designs were soluble (figs. S1B and S2A). Size-exclusion chromatography (SEC) profiles of representative alpha-helical and beta-barrel WRAP-OmpA proteins were monodisperse with single, well-defined peaks (Fig. 2, A and B). Circular dichroism (CD) spectra, melting temperatures, and differential scanning fluorimetry (DSF) showed that both designs were folded and stable at 95°C (Fig. 2, A and B, and figs. S3A and S4, A and B).

We used cryo–electron microscopy (cryo-EM) to characterize the structures of both alpha-helical (61.2 kDa) and beta-barrel (41.4 kDa) WRAPed-OmpA. We note that proteins in this size range are near the lower practical limit for cryo-EM and typically yield lower-resolution reconstructions without oligomerization or symmetry. Two-dimensional (2D) class averages and 3D reconstructions at 4.8-Å resolution for the helical WRAP-OmpA (Fig. 2, C and D) and 5.6 Å (Fig. 2, E and F) for the beta-barrel WRAP-OmpA were consistent with the intended locations of secondary structure elements of both the native OmpA beta-barrel and the surrounding designed WRAP after rigid-body docking of both design models into the corresponding cryo-EM density (Fig. 2, D and F). The WRAP structures highlight the generalization capabilities of RFdiffusion because there are very few concentrically nested beta-barrels in the PDB (none that are asymmetric monomers) and there are no hits [TM score > 0.6 (20)] that align to the WRAP-OmpA structures in searches against all available structure databases using Foldseek (21). To assess the preservation of antibody recognition elements in the WRAP constructs, we performed enzyme-linked immunosorbent assays (ELISA) with a polyclonal OmpA antibody and found that the WRAPs retained the corresponding epitopes, as did a native OmpA control purified with detergent and reconstituted into amphipols (Fig. 2G). Native mass spectrometry (MS) confirmed that WRAP-OmpA binds chitobiose, a native OmpA ligand (22) (fig. S5, A and B). Membrane protein ligands and cofactors are frequently lost during native MS due to the increased activation (heating) necessary to strip detergents, and the use of soluble WRAPed membrane proteins overcomes this limitation (23).

These data establish that our computational pipeline can generate custom alpha-helical or beta-barrel WRAP domains that result in soluble, thermostable versions of target transmembrane proteins. The beta-barrel WRAPs required substantially more computational power to achieve similar AF2 metrics (fig. S1C), likely due to the difficulty in satisfying the geometric constraints of interstrand hydrogen bonds for proper barrel formation while maintaining a complementary interface with the target. We therefore focused primarily on designing helical WRAPs for the remainder of our targets. Successfully solubilized designs are listed in the supplementary materials (fig. S2 and table S1).

### Solubilizing *Treponema pallidum* subspecies *pallidum* OMPs

Targeting OMPs in vaccine development primes the immune system to combat pathogenic bacteria such as *Treponema pallidum* subspecies *pallidum* (*Tp*), the causative agent of syphilis (24). To date, there are no reported experimentally determined structures of *Tp*-integral OMPs (24), and the production of wild-type proteins from *E. coli* has been challenging (25). Because of their potential as vaccine antigens, we aimed to solubilize four putative Tp OMPs: TP0126, TP0479, TP0698, and TP0733 (3, 24). These proteins are eight-stranded beta-barrels with transmembrane domains (TMDs) predicted with high confidence by AF2 (16) (pLDDT > 90; fig. S6). Starting from a small (~10 to 48) set of design models for each protein, we used a strategy similar to that described above for OmpA to screen for successful solubilization. After expression in *E. coli*, 15 to 30% of designs for each target were soluble and eluted at the expected size during SEC purification (fig. S2, B to E). Three designs with the most prominent SEC peaks were selected for characterization by analytical SEC and CD spectroscopy and were monodisperse and properly folded (Fig. 3, A to C). By contrast, *Tp* proteins linked as single fusions with Apo-AI failed to produce SEC peaks of the correct size (fig. S2F). 2D cryo-EM class averages of WRAP-TP0126 (72.1 kDa) and WRAP-TP0698 (49.9 kDa) indicated secondary structure elements matching those in the computational design models and AF2-predicted structures (Fig. 3, D and E). Although we were not able to generate a reliable 3D reconstruction of WRAP-TP126 due to issues with preferred orientation, a 4.0-Å-resolution 3D reconstruction of WRAP-TP0698 displayed secondary structural features consistent with the predicted structure after rigid-body docking of the design model into cryo-EM density (Fig. 3F). The density corresponding to the extracellular loops of TP0698 differed from the AF2 (16) prediction, which is not surprising because the prediction has very low confidence in this region (pLDDT < 50) (fig. S6).

To evaluate the antigenic properties of WRAP-TPs, we used sera from rabbits infected long term (>80 days) with *Tp*. ELISA experiments showed that the infected rabbit serum antibodies (postbleeds) had stronger reactivity to all three WRAP-TP proteins than to sera collected from the animals immediately before infection (prebleeds) (Fig. 3G). To date, no monoclonal antibodies targeting specific epitopes of these surface antigens are available. This is primarily due to the challenges associated with handling these highly hydrophobic proteins, which require renaturation after purification, often without certainty that their native structure has been fully restored. Additionally, the continued presence of detergents in solution is necessary for downstream applications, complicating antibody isolation and purification. Soluble scaffolds displaying single extracellular loops react with immune rabbit serum (26), but such scaffolds cannot be used to detect antibodies that bind epitopes involving multiple extracellular loops in native-like conformations or the beta-barrel core. Current efforts are directed at identifying the antibodies in the sera that react with the WRAPed antigens. In this way, WRAPed TP antigens could help to generate, isolate, and characterize monoclonal antibodies and could potentially have promise as components of a syphilis vaccine.

### WRAPed helical multipass transmembrane proteins retain enzyme active-site and ligand-binding properties

We next sought to solubilize the rhomboid protease GlpG and a C-X-C chemokine receptor type 4 (CXCR4) while maintaining enzymatic activity and the ability to bind ligands, respectively. GlpG, located in the inner membrane of *E. coli*, is one of the best biochemically characterized intramembrane proteases and cleaves its substrates directly inside the lipid bilayer (27). Three of six WRAP-GlpG designs were soluble, including both the wild-type GlpG [PDB ID: 2IC8 (28)] enzyme and a catalytic site mutant S201A+H254A (Fig. 4A and fig. S2, G and H). CD and DSF analyses indicated that WRAPed GlpG, like OmpA, was highly thermostable, retaining its structure even when heated to 95°C (Fig. 4A and fig. S4C). By contrast, native GlpG solubilized in detergent has a transition midpoint temperature of 77°C (29). Cryo-EM 2D class averages revealed clear secondary structural elements that agree well with the computational design model (Fig. 4B). We were also able to generate a 4.7-Å cryo-EM 3D reconstruction of WRAP-GlpG (74.3 kDa) and found it to be in close agreement with the computational design model (Fig. 4C) after rigid-body docking of the predicted model into the cryo-EM density map. To evaluate the activity of WRAP-GlpG, we used a nonspecific fluorophosphonate serine hydrolase probe, FP-TAMRA, which selectively labels functionally active enzymes, including GlpG (30). Time-dependent active-probe labeling showed that WRAPed GlpG accumulated substantial labeling over a 2-hour period, whereas labeling of the catalytic mutant was nearly undetectable (Fig. 4, D and E).

CXCR4 is a human G protein–coupled receptor (GPCR) with seven TMDs, and solubilizing it could facilitate high-throughput drug screening for this important cancer drug target (31). We first attempted to solubilize the full-length wild-type receptor [PDB ID: 4RWS (32)] using the WRAP pipeline described above; however, the protein was undetectable in the cytosolic fraction of *E. coli* (figs. S7 and S2I). Suspecting that aggregation was driven by the long hydrophobic transmembrane helices, we focused on WRAPing a truncated version (miniCXCR4) that preserves only the pharmacologically relevant surface of the GPCR, yielding a small, soluble mimetic suitable for drug screening. We retained the native sequence up to just below the ligand-binding pocket and connected the truncated transmembrane helices using short, flexible loops designed using RFdiffusion and ProteinMPNN to maintain structural integrity (Fig. 5A and table S1). We designed 46 truncated CXCR4 WRAPed variants (miniCXCR4 (66.2 kDa); Fig. 5B) and tested their expression with the WRAP fused either at the N or C terminus. Two of 46 WRAPed miniCXCR4 designs were found in the soluble fraction, one with an N-terminally fused WRAP and one with both N-and C-terminally fused WRAPs (Fig. 5C and fig. S2J). CD analysis showed that miniCXCR4-WRAP maintains stability even at 95°C, a substantial increase in thermostability compared with previously redesigned soluble GPCR variants (33) (Fig. 5D). To assess whether WRAPed miniCXCR4 has a properly folded extracellular surface, we measured binding to the native ligand CXCL12. Biolayer interferometry (BLI) measurements (Fig. 5E) indicated that the ligand bound to the miniCXCR4-WRAP with an apparent steady-state equilibrium dissociation constant (KD) of 390 ± 90 nM obtained by fitting equilibrium responses to a 1:1 binding model (errors represent standard errors of the fitted parameter). Thermal stability was evaluated by heating miniCXCR4-WRAP to 55° or 95°C for 15 min, followed by cooling at 25°C for 1 or 12 hours, before performing the binding measurements on BLI at 25°C. Although we observed differences in binding affinities after heat treatment (fig. S8, B to E) and prolonged incubation of the protein sample over 7 days at room temperature (fig. S8G), measurable binding of CXCL12 to the miniCXCR4-WRAP was retained, indicating that the protein remained folded and capable of binding. miniCXCR4-WRAP binding affinity for CXCL12 is similar to detergent-purified native CXCR4 previously reported by BLI (~156 to 180 nM) and using cell-based assays (~200 nM) (34) but lower than that measured using CXCR4 displayed on the surface of lentiviral particles (~37 nM) (35). Using native MS, we monitored binding of plerixafor, a small-molecule CXCR4 antagonist, to miniCXCR4-WRAP under charge-reducing conditions to preserve labile ligand interactions (36). Broad adduct peaks consistent with a plerixafor adduct were detected, consistent with formation of a 1:1 protein–drug complex. Tandem MS (MS/MS) analysis (37) confirmed the interaction of plerixafor with miniCXCR4-WRAP (fig. S5, C and D).

Together, these data show that WRAPs can preserve protein folds, help maintain enzyme active-site and ligand-binding properties, and enhance thermostability of the solubilized membrane proteins. We found that the WRAP approach can be applied to the second major class of membrane proteins, including the helical multipass membrane proteins that are of interest for drug discovery.

### Cryo-EM Structure of WRAPed oligomeric TMD of the mycobacterial porin

To extend our WRAP pipeline to oligomeric proteins, we used secondary structure and block adjacency to guide the diffusion-based placement of helices and to define interactions both between subunits and with the target (10). The outer membrane *Mycobacterium smegmatis* porin A (MspA) is a highly conductive octameric homo-oligomer that is widely used in nanopore DNA sequencing (38). We set out to wrap the MspA [PDB: 1UUN (39)]-insoluble TMD (stem) and pore constriction (base domains) (MspA_SBDs) to stabilize the loops critical for sequencing. We excluded the MspA rim and extracellular domains (N and C termini) because they are already soluble, which simplified design and reduced computational time and complexity. Eighty-three–residue helices were added to the N terminus and guided through a block-adjacency matrix to interact with each beta-strand of a monomer, as well as with neighboring helices within the WRAP (WRAP-MspA_ SBD). The secondary structure and block adjacency information (fig. S9, A and B) was then provided to RFdiffusion to generate a WRAP as described above while also specifying C8 symmetry to accommodate the oligomeric architecture. One of eight designs tested was well expressed and soluble and had the expected SEC profile (Fig. 6A and fig. S2K). WRAP-MspA_SBD remained fully folded up to 75°C, and no transition temperature was observed in the CD temperature melt profiles (Fig. 6A and figs. S3B and S4, D and E). Full-length MspA is thermostable up to 100°C (40); with C-terminal methionine 183 contributing to thermostability and oligomerization through interaction with N-terminal tryptophan 40 (41) (both removed in MspA_SBDs). Deletion of the last three residues (182 to 184) of native MspA abolishes detectable protein expression (41). By contrast, our WRAPed-MspA_SBDs construct has 62 C-terminal residues (122 to 184) deleted, yet it is expressed at higher levels than wild-type MspA, indicating that WRAPing compensates for the loss of N-and C-terminal stabilizing interactions.

Given the substantially larger size of WRAP-MspA_SBD relative to the other protein complexes analyzed in this study, we selected this construct as the primary target for structural determination with the goal of assessing the accuracy of our design pipeline at high resolution. In contrast to the smaller WRAP constructs, which were primarily evaluated for secondary structure agreement and not pursued with similarly extensive data collection, the larger size of this construct enabled deeper data collection and optimized acquisition strategies, including multiple grid types and stage-tilt angles to mitigate preferred orientation. This issue was also observed in the smaller WRAPed protein samples but could not be effectively addressed in those cases using similar strategies due to a much lower signal-to-noise ratio. The resulting cryo-EM analysis of purified WRAP-MspA_SBD (136.7 kDa) revealed that the designed C8 oligomers represent the predominant species in solution, although minor off-target cyclic assemblies were also detected (fig. S10B). Representative 2D class averages showed clearly resolved secondary structure features in the top, side, and tilted views, revealing both the expected C8 symmetry along the major axis and an unexpected dimerization between C8 units along the minor axis (Fig. 6B). This broad angular coverage, coupled with the high molecular weight of WRAP-MspA_SBD, enabled the reconstruction of a 2.9-Å-resolution map with D8 symmetry enforced and a 3.3-Å-resolution map with C1 symmetry, both of which showed well-defined density spanning the MspA_SBDs and the designed WRAP domain (Fig. 6C, figs. S10 to S12, and table S4). Local resolution estimates for these reconstructions indicated comparable resolution for the MspA_SBD core and the WRAP domains, consistent with limited relative flexibility of WRAP with respect to the MspA_SBD scaffold and supporting the presence of a well-ordered interface (figs. S10 and S11). This high-resolution map supported model building at all levels of structural hierarchy, resulting in a complete atomic model of the designed complex (Fig. 6D, figs. S10 to S12, and table S4). Comparison of the cryo-EM structure with the original C8 design model revealed close agreement: The C8 component aligned with the design model with a root mean square deviation (RMSD) of 0.7 Å (Fig. 6E), and a focused comparison of only the MspA_SBDs yielded an even closer match (RMSD = 0.5 Å; Fig. 6F). The MspA_SBDs from the cryo-EM structure also closely aligned with the previously solved structure of full-length MspA, which had been determined using classic detergent-based solubilization methods. This comparison confirmed that the WRAPed MspA_SBDs retained both their fold and quaternary configuration (RMSD = 0.4 Å; Fig. 6G). The D8 dimerization observed in the cryo-EM structure is recapitulated in the crystal lattice of the purified native protein, where it is mediated entirely by the WRAPed MspA_SBD, suggesting that the higher-order assembly is an intrinsic feature of the native protein and not induced by the WRAP (fig. S12). A close-up comparison with the crystal structure further demonstrated that the key side-chain rotamer aspartate 91, which is essential for maintaining the constriction of the pore (38, 39), is preserved in the WRAPed complex (Fig. 6H). Together, these findings show that oligomeric proteins can be solubilized using WRAPs, and that the WRAP platform enables high-resolution structure determination of membrane proteins in solution.

## Conclusions

We introduce a method for solubilizing and solving structures of native transmembrane proteins in solution using genetically encoded, designed auxiliary domains. WRAPing requires neither detergents nor refolding, enabling expression in the *E. coli* cytoplasm with enhanced thermal stability while preserving native sequence, structure, and function. Unlike previous approaches (5–8), WRAPs are tailored to the membrane protein of interest, requiring no modification of the native sequence. Cryo-EM 2D class averages and 3D reconstructions of soluble WRAPed OmpA and GlpG show clear secondary structural features and preserved geometry, and rigid-body docking demonstrating strong agreement between the density and the design models, indicating that WRAP does not perturb the underlying protein structure. Although the reconstructions of OmpA (~60 kDa), TP (~49 kDa), and GlpG (~74 kDa) did not reach model-building resolution, this is consistent with known limitations and challenges associated with single-particle analysis of proteins smaller than ~100 kDa (42). The agreement between the 2D averages and moderate resolution maps to the rigidly docked computational design models indicates that the WRAP strategy functions as intended. This conclusion is further supported by a 2.9-Å high-resolution cryo-EM structure of WRAP-MspA_SBD, which demonstrated that this approach can support high-resolution cryo-EM structure determination of membrane proteins. WRAP design does not require experimentally determined structures, as illustrated by the solubilization of *Tp* outer membrane beta-barrels starting from AF2 models.

Like the SIMPLEx approach (9), WRAPs enable purification of membrane proteins directly from the *E. coli* soluble fraction, a key advantage over nanodiscs (43) and other amphipathic systems (44, 45), which require identifying optimal detergent conditions before reconstitution into those systems. Typical yields of WRAP-purified proteins range from 0.5 to 15 mg per liter (46–50) depending on the target, showing that WRAPs can match or exceed yields obtained through detergent-based extraction while substantially simplifying the workflow and reducing reagent costs. The WRAP and SIMPLEx approaches have complementary advantages: SIMPLEx involves fusion to a general carrier protein and so requires no custom design, and WRAPs instead require custom protein design (currently limited to proteins of ~500 amino acids or fewer) to create more precisely interacting, target-specific solutions, which in turn enables structural determination (51).

WRAPs could have broad utility across a wide range of applications. For vaccines, WRAPs allow the expression of complex, membrane-associated antigens in soluble form, as illustrated here for the *Tp* outer membrane antigens, facilitating their incorporation into effective immunogens. The sequences of our WRAPs are synthetic and lack substantial sequence homology to known human proteins. This could be useful in the context of vaccination, for which eliciting antibodies that target self-proteins [e.g., Apo-AI (9)] is undesirable. More generally, by enabling the generation of soluble versions of extremely difficult to characterize antigens such as the *Tp* OMPs, WRAPs should contribute to diagnosing and combating bacterial diseases that have been limited by the difficulty in producing and characterizing the major surface antigens. For nanopore-based devices, the WRAPing of membrane nanopores such as MspA could enable incorporation into more robust supportive solid-state systems beyond lipid membranes. For drug development, WRAPs could contribute to the generation of soluble, thermostable, and antigenically intact versions of critical membrane protein targets for high-throughput small-molecule and antibody drug screening and downstream structural studies of drug-target interactions.

In the case of small (i.e., <100 kDa) WRAPed membrane protein constructs, structure determination will likely benefit from further optimization of cryo-EM sample preparation, data collection, and processing strategies, particularly to address the preferred orientation and low signal-to-noise ratio that limited high-resolution refinement for many of the smaller constructs analyzed here. The size and stability of many WRAPed membrane proteins also make them strong candidates for x-ray crystallography. For biochemical characterization, the ability to purify targets without detergent could facilitate the identification of detergent-sensitive bound cofactors and, more generally, enable the measurement of binding interactions without possible perturbation by detergent. Continued developments in the deep learning design methodology for generating WRAPs are expected to reduce computational cost, improve prediction of successful solubilization, and lower the number of tested constructs. Such improvements could further advance the applications described here by expanding applicability to full-length GPCRs and enabling tighter, more conformationally restrictive WRAPing to facilitate high-resolution cryo-EM of smaller membrane proteins.

## Material and Methods

### Fold-conditioned helical WRAPs

Fold conditioning or secondary structure adjacency matrices, first introduced by Anand *et al.* (52) to guide diffusion-based protein backbone generation, allow to very specifically define the protein topology. The original RFdiffusion paper provides a detailed explanation of how secondary structure and block-adjacency features are used, including the shapes of these tensors and what each dimension represents (10). In brief, fold conditioning to guide RFdiffusion scaffold generation first requires a 2D block adjacency matrix that defines which blocks of secondary structures are in contact (e.g., every secondary structure element separated by loops is considered an individual block) and is described as an N_residue_ x N_residue_ 2D tensor. Secondary structure block contacts are depicted with a 1 and non-contacts with a 0. The other input is the secondary structure tensor, which is a 1D tensor of the size N_residue_ and guides the secondary structure of each residue position (0, helix; 1, beta-sheet; 2, loop; 3, mask). Adjacency matrix for symmetric helical hairpin oligomer (C8) was generated using fold-conditioned diffusion and connected into monomeric backbones with 16 helices (h16) using RFdiffusion (10). Additional details of the code used can be found in the provided scripts at https://github.com/davidekim/WRAPs, archived on Zenodo (53). The length and number of helices were determined based on the hydrophobic thickness and size of the transmembrane-spanning region of the target protein. The target was translated into the center of the monomeric WRAP (chain A) backbone and saved as chain B (membrane protein) into a single PDB using the PyMOL Molecular Graphics System [version 3.0, Schrödinger, LLC (13)], which was used as an input for partial diffusion (10). Any additional atoms or protein complexes were removed from the target PDB, and the clean version was used as an input. Careful consideration was given to the placement of each target within its WRAP to minimize the distance between the termini of the target and the WRAP that would subsequently be fused with a linker, determining the relative configuration of WRAP and the target in the gene construct. Partial diffusion was performed with “diffuser.partial_T” input values of 15 or 20 [the number of diffusion steps used in the model’s denoising trajectory (10)], generating ~20 partially diffused WRAP backbones for each target. Only the first 15 to 20 steps of the diffusion process were necessary to generate optimal backbone-to-backbone interactions between the WRAP and the target (rather than running the full 50 steps), reducing compute time by more than half. Two WRAP sequences per backbone were designed using soluble ProteinMPNN (8, 15), while keeping the target protein sequence fixed. The resulting designs were filtered based on AF2 (16) pAE_i and pLDDT_binder scores (see table S1 for more details on filter cutoffs for each design). We used this approach to design WRAPs for helical OmpA, GlpG, heliorhodopsin, aquaporin M, and TP0733.

### Pseudosymmetric helical WRAPs

For targets that were more challenging to express in *E. coli*, such as full-length CXCR4 (flCXCR4) and *Tp* OMPs lacking experimental structures, we implemented an expanded WRAP design strategy based on pseudosymmetric fold-conditioned diffusion to increase backbone diversity. Instead of deriving monomeric h16 backbones from symmetric oligomeric assemblies, we directly generated diverse monomeric WRAP backbones using pseudosymmetry constraints in RFdiffusion. Helix number, length, and connectivity were selected based on the estimated dimensions of the target transmembrane region. For TP0698 and TP0497, the monomeric WRAP scaffold was generated using pseudo-C6 (pc6) symmetry, consisting of two 18-residue helices and two 4-residue loops in each asymmetric unit. For TP0126, the monomeric wrap scaffold was generated using pseudo-C8 (pc8) symmetry, consisting of three 15-residue helices and two 4-residue loops in each asymmetric unit. For flCXCR4-WRAP, the monomeric WRAP scaffold was generated using pseudo-C7 (pc7) symmetry, consisting of three 24-residue or 20-residue helices and three four-residue loops in each asymmetric unit. Lastly, for WRAP-miniCXCR4, the monomeric WRAP scaffold was generated using pseudo-C7 (pc7) symmetry, consisting of three 16-residue helices and two four-residue loops in each asymmetric unit. Intra-and intercomponent connectivity constraints were specified to enable diverse helix-helix packing while preserving a defined topology. Fifty WRAP backbones were generated using this approach, after which each target was aligned in the center of the WRAP backbone using PyMOL and subjected to partial diffusion using diffuser.partial_T values of 15 and 20 for *Tp* OMPs and additional 25 and 30 for miniCXCR4 and full-lengthCXCR4. For each backbone and partial_T value, 10 partially diffused structures were generated. Sequence design was then performed using soluble ProteinMPNN with the target sequence fixed, sampling 10 sequences at sampling temperatures of 0.1 and 0.2 for each of the partially diffused structures. All designs were subsequently folded using AF2 and filtered using predicted structural and interface quality metrics, including per-residue confidence (pLDDT_binder), predicted aligned error at the binding interface (pAE_interaction), and RMSD of backbone Cα atoms between the predicted structure and the design model (binder Cα RMSD). All designs except full-lengthCXCR4 were further filtered using solvent-accessible surface area–weighted hydrophobicity score (SAP_score) (54) to penalize exposed hydrophobic surfaces and to reduce aggregation propensity. For full-lengthCXCR4, SAP_ratio was calculated in place of SAP_score because SAP_score is an extensive quantity that scales with protein size. For calculating SAP_ratio, SAP_scores were normalized by sequence length to obtain a per-residue metric, enabling comparison across constructs of different lengths generated for full-lengthCXCR4. All filtering thresholds and additional design criteria are reported in table S1. Finally, subsequent manual curation of the designs was performed to select structurally diverse backbones and maintain a manageable experimental scale. On the basis of these criteria, 48 TP0126, 24 TP0698, 24 TP0497, 49 flCXCR4, and 46 miniCXCR4 WRAPed designs were filtered and selected for experimental testing.

### Parametrically guided design of beta-barrel WRAPs

The pipeline used to design beta barrel WRAPs was adapted from a previous study (11) and is briefly described here. Parameter-defined C-alpha atom cylinders (56, 57) were made using strand count (*n*; 10 to 19), shear number (S, the register shift between the N and C terminal strands; 14 to 26), and strand length (6 to 11) parameters to match luminal radii (13 < *r* < 21) and length ranges (19 < l < 35) that would cover the hydrophobic lipid facing surface of native OmpA. Model 5 of the PDB NMR ensemble from 2GE4 (19) was used as the target structure and the transmembrane residues were predicted using DeepTMHMM (58). N and C backbone atoms were placed onto the C-alpha only cylinders to complete the backbone using BBQ [Backbone Building from Quadrilaterals (59)]. The resulting cylinder was placed onto the target using PyRosetta (14) by superimposing the center of masses of the top (outer membrane), bottom (inner membrane), and all transmembrane-spanning residues of the target with the center of masses of the top, bottom, and all residues of the cylinder. Two residue termini and three residue loops connecting each beta-strand pair in the placed cylinder were roughly built with the loop modeling mover in PyRosetta, and the completed barrel backbone was rotated around the central axis until the distance between the termini of the barrel and target to be fused was at the minimum (using PyRosetta). The resulting 230 two-chain complexes were used as the input for partial diffusion using diffuser.partial_T of 15 to generate ~2300 partially diffused backbones. Sequence design was then performed using soluble ProteinMPN (8, 15) FastRelax (59) followed by AF2 (16) validation. Because only five designs had AF2 pAE_interaction <15 with pLDDT_binder <75 (a success rate of 0 compared with the helical WRAP success rate of ~7% with pLDDT_binder >90), we took an iterative optimization approach for 265 partial diffusion outputs selected based on the quality of the barrel WRAP (interstrand hydrogen bonding covering at least 30% of each parameter based strand pair) (11). Each iteration consisted of 20 partial diffusion trials (including 10 trajectory time-point models for each diffusion run), Soluble ProteinMPNN (8, 15) sequence design of the 220 resulting partially diffused backbones, and AF2 (16) structure prediction. The AF2 model with the lowest pAE_interaction <15 was used as the input for the next iteration of the computational pipeline for up to 10 iterations. This optimization strategy was run separately for each of the 265 initial partial diffusion outputs for a total of ~437,000 AF2 models; 9900 designs had pLDDT_binder >90 (2.3% success). Six designs were selected and ordered based on manual inspection of 81 designs with pLDDT_binder >90, pAE_interaction <5.2, and barrel WRAP quality with at least 50% interstrand hydrogen bond coverage.

### Oligomeric WRAPs

Fold conditioning was recently used to develop binders to exposed beta-sheets through strand extension (60). We adapted these feature by padding the adjacency matrix with the desired number of residues. In the case of MspA_SBDs, two helices with 80 residues total (connected by a three-residue loop) were added to the N terminus of the MspA with truncated extracellular domains (https://github.com/davidekim/WRAPs). Next, each of the newly added secondary structure elements was guided to interact with the native secondary structure elements and with each other by creating an adjacency matrix (fig. S9, A and B). The final version of the adjacency matrix is shown in fig. S9B and was used to scaffold guide RFdiffusion with “diffuser.T” of 30 steps total (10), applying C8 symmetry. The remaining steps of the pipeline proceeded as described earlier, using soluble ProteinMPNN (8, 15) and AF3 (61) prediction with C8 symmetry. Eight designs with pLDDT >70 and minPAE_i <8 were selected for experimental testing. Our best design has one 40-residue helix divided into two shorter helices interacting with beta-sheets and the other 40-residue helix extending all the way to the constriction loops of MspA. In silico pipelines to generate WRAPs can be run on a standard desktop computer with a CUDA-enabled NVIDIA GPU with 8 to 16 GB of VRAM. Generally, run times can vary from 10 to 30 min on a T4 GPU to generate a WRAP design for a 200-residue target.

### Design ordering, gene construction, protein expression, and purification in *E. coli*

Single-gene fusions of WRAP designs and native membrane protein sequences (excluding signal sequences and intracellular domains) were constructed using a flexible GS linker (fig. S1B and table S2). In table S2, native membrane protein sequences are listed in italic font, WRAP sequences are underscored, and experimentally characterized designs are shown in bold. MspA and CXCR4 sequences contained additional truncations; the rationale for not using the full-length proteins is described in the main text. In an initial pilot experiment, six designs per target were ordered with either N-or C-terminal WRAP fusions based on the distance between the WRAP and target termini as predetermined during the design process. In addition, some constructs included an N-terminal maltose-binding protein (MBP) fused to the target through a GS linker containing a tobacco etch virus (TEV) protease site (fig. S1B) based on the initial hypothesis that a soluble N-terminal domain might be required to prevent membrane targeting (9). Adding a soluble decoy proved unnecessary, and both N- and Cterminal configurations could successfully yield soluble protein. However, as a general rule, we recommend initially placing the WRAP on the N terminus of membrane proteins that do not require a free N terminus for proper function. For Apo-AI fusions with TP proteins, genes encoding human Apo-AI (9) were ordered in exact N-or C-terminal configurations analogous to the WRAP designs without additional tags. For experimentally challenging targets (e.g., CXCR4 and TP OMPs), a larger number of designs were ordered to increase the likelihood of successful solubilization. The number of designs tested reflects a balance between the number of designs satisfying filtering metrics, design diversity, target difficulty, and practical considerations for experimental screening (our medium-throughput experimental pipeline can accommodate up to 96 designs per plate; see table S1 for additional details).

The constructs were synthesized as eBlock gene fragments by Integrated DNA Technologies (IDT) and cloned into the pET29b+ vector using Golden Gate assembly. These plasmids were then transformed into chemically competent BL21(DE3) E. coli cells (NEB) for protein expression. Each design was expressed overnight at 37°C in 1 ml/well of autoinduction medium, which consisted of Terrific Broth II (MP Biomedicals), 2 mM MgSO4, 1x5052 (25% v/w glycerol, 2.5% v/w D-glucose, and 10% w/v D-lactose monohydrate), and 50 μg/ml kanamycin in a 96-well round-bottom 1 ml/well plate placed on a platform shaker (1000 rpm). After expression, four 96-well plates were pooled together, and cells were lysed using 300 to 400 μl of BugBuster (Millipore) in the presence of DNAse and Pierce protease inhibitor tablets (Thermo Fisher Scientific). Subsequently, immobilized metal affinity chromatography (IMAC) purification was carried out in a 24-μm polyethylene frit 96-well plate (Agilent Technologies) with 75 μl/well Ni-NTA affinity resin (QIAGEN). After lysates were loaded onto the resin, proteins were washed three times using 100 mM NaCl, 25 mM Tris-HCl, and 30 mM imidazole at pH 8.0. Proteins were then eluted in 150 μl of 25 mM Tris-HCl, 500 mM NaCl, and 400 mM imidazole at pH 8.0 and further purified by either Akta Pure FPLC (GE Healthcare) or Agilent Tecnhologies high-performance liquid chromatography (HPLC) SEC using a Superdex 200 increase 10/300 GL or 5/150 GL column.

For large-scale expression, cultures were grown in 50 ml or 0.5 liters of the autoinduction medium in baffled flasks overnight at 37°C. Cells were lysed using a sonicator (50 ml) or a microfluidizer (0.5 liters) in the lysis buffer (100 mM NaCl and 20 mM Tris) supplemented with Pierce protease inhibitor tablets (Thermo Fisher Scientific). IMAC was performed as described above using a column with 0.5 (50 ml) or 2.5 ml (0.5 liters) of affinity resin and washing with 10 column-volumes and eluting with two column-volumns. Proteins were further purified by SEC as described above, and fractions corresponding to monodispersed peaks that were monomeric based on calibration standards were collected and stored at 4°C or frozen at −80°C for subsequent biophysical characterization experiments. WRAPs with N-terminal MBP fusions were incubated overnight at room temperature with TEV protease (1:100 protease to WRAP w/w ratio) and repurified using IMAC purification followed by SEC.

### CD

Purified designs were prepared at 0.2 to 0.4 mg/ml in the 20 mM Tris buffer (100 mM NaCl, pH 8.0) and for MspA_SBD, 20 mM phosphate buffer (100 mM sodium sulfate, pH 8.0). Wavelength scans from 200 to 260 nm were recorded at increasing temperatures ranging from 25° to 95°C, and temperature melts were recorded at 220 or 222 nm (217 nm for the beta-barrel WRAPed OmpA) in 5° increments from 25° to 95°C using a Jasco J-1500 CD spectrophotometer with a 1-mm path length cuvette.

### DSF and static light scattering

Protein unfolding and aggregation were monitored by measuring changes in intrinsic fluorescence and static light scattering (SLS), respectively, using the Uncle stability screening platform (Unchained Laboratories). The protein samples were heated from 25° to 93°C at 2° increments and 180-s incubation periods using protein concentrations of ~0.05 to 1.5 mg/ml. The fluorescence emission signal, excited at 266 nm, from 280 to 450 nm was collected at each temperature increment to monitor unfolding. Protein aggregation was also estimated from the SLS intensity measured using a 266-nm laser. Monarch RNase A (NEB) at 1 mg/ml in 20 mM Tris-HCl (150 mM NaCl, pH 8.0) was used for reference.

### Native MS

WRAP-OmpA and miniCXCR4-WRAP were buffer exchanged immediately before MS analysis into 0.5 M ammonium acetate (pH 6.9) and 0.1 M ammonium acetate (pH 6.9), respectively (Sigma), and loaded into nESI borosilicate capillaries (Thermo Fisher Scientific). For chitobiose-binding studies, 10 μM WRAP-OmpA was incubated with 1 mM N,N′-diacetylchitobiose (Sigma), and unbound ligand was removed by gel filtration using ZebaSpin columns (Thermo Fisher Scientific). For CXCR4-binding studies, 10 μM miniCXCR4-WRAP was incubated with 100 μM plerixafor (Sigma) at 37°C for 30 min in 0.5 M ammonium acetate. Mass spectra were acquired in positive ionization mode on a modified Waters Synapt G1 traveling-wave ion mobility mass spectrometer (MS Vision) using a capillary voltage of 1.5 kV and a sample cone voltage of 50 V. Chitobiose binding was confirmed by MS/MS of the 14+ charge state (m/z 4480, corresponding to one protein molecule with two chitobiose adducts) using a trap voltage of 50 V. Plerixafor (Selleckchem) binding was confirmed by MS/MS of the 17+, 16+, and 15+ charge states, using the predicted m/z values of 3920, 4165, and 4443, respectively, for the 1:1 complex as precursor ions. The source temperature was set to 30°C, and trap and transfer collision energies were maintained at 10 V. Argon was used as the trap gas at a flow rate of 3 ml/min, and nitrogen was used as the ion mobility gas at 30 ml/min. Data were analyzed using MassLynx 4.1 (Waters).

### Enzyme assays

For enzyme assays, 1 μM ActivX TAMRA-FP Serine Hydrolase Probe (Thermo Fisher Scientific) was incubated with 1 μM WRAPed enzyme in 20 mM HEPES with 50 mM NaCl buffer at pH 7.0. The reaction was collected at 0, 1, 5, 10, 15, 30, 60, and 120 min and quenched using the sample loading buffer. The samples were stored at −20°C until all of the time points were collected and loaded on the gel. FP-TAMRA was visualized using LI-COR imaging system with a 520-nm laser, and Coomassie blue was used to stain total protein loaded on the gel.

### BLI

BLI measurements were performed at 25°C using the Octet Red96 system (ForteBio, Sartorius) with streptavidin-coated biosensor tips. Measurements were performed in 1× HBS-EP+ buffer (Cytiva BR100669) supplemented with 0.05% (w/v) bovine serum albumin (BSA) to minimize nonspecific binding. Biotinylated human CXCL12 (Chemotactics B-CXCL12) was immobilized at 50 nM to a loading threshold of 0.5 nm. After a 60-s baseline, association and dissociation were monitored for 400 and 600 s, respectively. A reference sensor loaded with ligand alone was used for baseline subtraction. Steady-state equilibrium dissociation constants (KD) were determined by plotting equilibrium responses (R_eq_), obtained from the plateau of the association phase as a function of analyte concentration and fitting to a 1:1 binding model using Octet Data Analysis software (v12.0.2.3). Errors represent standard errors of the fitted parameter. Kinetic parameters (k_on_ and k_off_) were obtained from global fits to a 1:1 binding model, with errors representing standard errors from the fit. For thermal stability measurements, purified miniCXCR4-WRAP samples were heated to 55° or 95°C for 15 min, followed by equilibration at 25°C for 1 hour or 12 hours before assessing binding on BLI. All binding measurements were conducted at 25°C. For temporal stability, samples were incubated at room temperature for 7 days before measurement. In addition, binding was assessed for concentrated, non–heat-treated sample at 25°C. All measurements and fits are summarized in table S5 and fig. S8, respectively.

### ELISA

For ELISA, 1 μg/ml of protein was incubated with MaxiSorb plates in 100 μl of 100 mM NaCl with 25 mM Tris (pH 8.0) at room temperature for 60 min or overnight at 4°C. All subsequent incubations were performed at room temperature. Blocking was done with 3% BSA in 100 mM NaCl, 25 mM Tris, and 0.05% Tween at pH 8.0 (TBS-T) for 60 min. Primary polyclonal rabbit OmpA antibody (Antibody Research Corporation) was serially diluted in blocking buffer at 1:3 starting at a concentration of 10 μg/ml. For the WRAPed TPs, immunized rabbit sera was serially diluted in blocking buffer 1:2 from an initial dilution of 1:20. Primary incubation (100 μl/ml) was done for 1 hour at room temperature. After incubation, plates were washed three times with TBS-T. Secondary antibody (goat anti-rabbit HRP, Invitrogen 31460) was added in 1:2000 dilution at 100 μl/well and incubated for 30 to 60 min. After washing the plates three times with TBS-T, 100 μl TMB substrate (Seracare, 5120-0083) was added and incubated for no more than 10 min before quenching with 100 μl of 1 M HCl. Then, absorbance was recorded at 450 nm.

### Immune Rabbit Sera

Immune rabbit serum was obtained from long-term (≥80 days) infected animals. Animal care was provided in accordance with the Guide for the Care and Use of Laboratory Animals from the National Institutes of Health, and all experimental procedures were conducted under protocol 4243-01 (PI: Lorenzo Giacani), which was approved by the University of Washington institutional animal care and use committee. Animals were purchased from the Western Oregon Rabbit Company (Philomath, OR) and housed at the Animal Use and Care Facility (ARCF) at the University of Washington. Infection was performed by intradermal inoculation of adult male New Zealand White rabbits (n = 6) with live 1 × 107 treponemes total (Nichols strain) evenly distributed across 10 sites on the animals’ shaved backs. Before infection, all animals were tested with both the Venereal Disease Research Laboratory (VDRL) test and the Treponema pallidum particle agglutination (TPPA) tests and found to be nonreactive. After infection, immune status was monitored periodically by performing VDRL and TPPA and tested using a pan-proteomic array carrying all Tp proteome as previously described (62), which confirmed the development of humoral immunity to most annotated Tp OMPs. At the end of the experiments, euthanasia was performed consistent with the recommendations of the Panel on Euthanasia of the American Veterinary Association. Sera were absorbed with 140 μl of *E. coli* lysate overnight at 4°C to remove nonspecific binding.

### Cryo-EM sample preparation

To prepare grids for freezing, grids were glow discharged on a PELCO easiGlow (Ted Pella). Then, R2/2 C-flat holey carbon grids (EMS CFT-223C-100) were glow discharged under vacuum of 0.39 mBar at 15 mA for 25 s. Quantifoil R2/2 holey carbon grids with a continuous 2-nm thin carbon film (EMS Q3100CR2-2nm) were glow discharged under vacuum of 0.39 mBar at 5 mA for 16 s. Next, 2 μl of protein for OmpA, GlpG, TP0126, TP0698, and MspA (at concentrations between 0.5 and 1 mg/ml) was applied to prepared C-flat R2/2 thick holey carbon grids (EMS CFT-223C-100). Vitrification was performed using a Vitrobot MkIV (ThermoFisher Scientific) at 22°C and 100% humidity, with a wait time of 7.5 s, a blot time of 6.5 to 7.5 s, and a blot force of 0, before being immediately plunge-frozen into liquid ethane. For MspA only, additional data were collected with Quantifoil R2/2 holey carbon grids with a continuous 2-nm thin carbon film (EMS Q3100CR2-2nm), and 3 μl of protein (at concentrations of 0.5 and 1 mg/ml) was applied to the grid. Vitrification was performed using a Vitrobot MkIV (ThermoFisher Scientific) at 22°C and 100% humidity, with a wait time of 7.5 s, a blot time of 0.5 s, and a blot force of 0 or −1 before being immediately plunge-frozen into liquid ethane. After plunge-freezing, grids were clipped following standard protocols before being loaded into a 300-kV Titan Krios (ThermoFisher Scientific) for imaging.

### Cryo-EM data collection

Data collection was automated using SerialEM (63, 64) to control a 300-kV Titan Krios (ThermoFisher Scientific) equipped with a K3 direct detector (Gatan) operating in correlated double-sampling mode and a BioQuantum energy filter (Gatan). Cryo-EM data were collected using either counting mode (for OmpA, GlpG, and MspA) or super-resolution mode (for TP0126 and TP0698), with random defocus ranges spanning −0.8 to −1.8 μm and image shift acquisition using four or five shots per hole. A total of ~5000 movies per sample were collected, with the exception of MspA, for which 12,234 movies were acquired. To improve angular sampling, MspA data were collected across multiple tilt angles (0°, 15°, 25°, and 30°). The calculated pixel sizes were 0.84 Å/pixel (OmpA and GlpG), 0.83 Å/pixel (WRAP_MspA-SBDs), and 0.42 Å/pixel (TP0126 and TP0698), with a total dose of ~50 e–/Å2.

### Cryo-EM data processing and structural characterization of OmpA, GlpG, TP0126, and TP0698

All data processing was conducted in CryoSPARC (65). Alignment of movie frames was performed using Patch Motion with an estimated B-factor of 500 Å2 and a maximum alignment resolution set to 5 Å. Defocus and astigmatism values were estimated using the Patch contrast transfer function (CTF). Initial particle picking was reference-free using Blob Picker, with particles extracted using a box size at least twice the estimated particle diameter. This was followed by a round of 2D classification and subsequent template picking using the best 2D class averages low-pass filtered to 20 Å. Particles were then repicked with Template Picker, manually inspected, and re-extracted for a second round of reference-free 2D classification.

During this step, particles were sorted into at least 200 to 250 classes, with a maximum alignment resolution typically set to 4 Å over the course of 75 initial iterations and 25 final iterations. The best classes, which revealed clearly visible secondary structural elements, were used for 3D ab initio determination with the C1 symmetry operator. Batch sizes were increased from the default value to between 300 and 500, and the initial and final resolution values were adjusted to 15 and 12 Å, respectively. This was followed by either a round of 3D heterogeneous refinement using C1 symmetry or nonuniform refinement using C1 symmetry.

For OmpA and GlpG, for which class averages agreed well with the design model and the structures were already known, a low-pass-filtered map of the computationally designed WRAPed protein was used as an initial model for refinement protocols instead of the output ab initio maps. Final resolution estimates were obtained for four of the five samples. TP0126 was excluded because the resulting maps were low quality (presumably due to poor angular sampling), and only 2D class averages were analyzed. The resolutions were as follows: helix OmpA-WRAP, 4.8 Å; barrel OmpA-WRAP, 5.6 Å; GlpG-WRAP, 4.7 Å; and TP0698-WRAP, 4.0 Å. Given the small nature of these particles and the lower resolution of these maps, design models were rigid-body docked into the corresponding density in UCSF ChimeraX (66) to confirm proper folding, assembly, and agreement with the computational design models. No atomic modeling or flexible fitting of the computational design models was performed for any of these samples.

### Cryo-EM data processing and structural characterization (WRAP-MspA_SBDs)

All data processing was conducted in CryoSPARC v4.6.0, and standard settings were used unless otherwise noted. Alignment of movie frames was performed using Patch Motion Correction with an estimated Defocus and astigmatism values were estimated using Patch CTF estimation. A total of 12,234 movies (2,138 movies with thin carbon grids and 10,096 holey carbon grids) collected from three grids were denoised using Micrograph Denoiser in CryoSPARC. Initial particle picking was done on a small subset of 1777 micrographs using a reference-free Blob Picker tuned to a minimum particle diameter of 70 Å and a maximum particle diameter of 90 Å. A total of 2,166,553 initial picks were inspected with Micrograph Junk Detector and Inspect Particle Picks to remove false-positive picks before particle extraction of 1,757,856 particles with a box size of 350 pixels. 2D classification was used with 200 classes and a circular mark diameter of 120 Å. Most of the classes seen were only top views of WRAP-MspA_SBDs octamers and smaller population of heptamers and nonagons (fig. S6B). One class observed was a side view of a WRAP-MspA_SBDs octamer that had dimerized with another WRAP-MspA_SBDs octamer. This dimerized class was used as a template to pick on all 12,234 micrographs using Template Picker with particle diameter 120 Å, denoised micrographs true, CTFs to filter the templates false, and maximum number of local maxima to consider 1000. After Inspect Picks, 1,957,111 particles were extracted with a box size of 350 pixels for 2D classification with 300 classes, circular mask diameter 120 Å, number of online-EM iterations 75, and number of final full iterations 25. Seven classes with 81,371 particles were selected showing side and tilted views of the octameric WRAP-MspA_SBDs dimer (Fig. 5D). Ab Initio reconstructions were generated with two volumes and C8 symmetry applied. The best volume was used for a Non Uniform Refinement with C8 symmetry and number of extra final passes 5. A structure of the octameric WRAP-MspA_SBDs dimer resolved 3.77 Å; however, the one side of the dimeric complex had weaker density, suggesting the presence of WRAP_MspA-SBDs monomers in the dataset.

To remove this, we also solved a low-resolution map of the octameric WRAP-MspA_SBDs monomer. Nine 2D classes of octameric WRAP-MspA_SBDs monomers with top and slightly tilted views totaling 254,863 particles were used to generate an Ab Initio with C8 symmetry applied and two classes used. Using the best map, a Non Uniform Refinement job was run with symmetry set to C8 and the number of final passes set to 5. A low-resolution map resolved to 6.59 Å with preferred orientation issues; however, the shape roughly resembles the WRAP-MspA_SBDs monomer and could be used for further heterogeneous refinement.

Using both the octameric WRAP-MspA_SBDs monomer and dimer maps, a Heterogeneous Refinement job was run with 86,323 particles from the octameric WRAP-MspA_SBDs dimeric 2D classes. A total of 67,001 particles generated an octameric WRAP-MspA_SBDs dimeric structure and 14,370 particles generated an octameric WRAP-MspA_SBDs monomeric structure. Using the 67,001 particles and the previously solved map of the 3.77-Å octameric WRAP-MspA_SBDs dimer as an input for Non-Uniform Refinement, a 3.22-Å map was generated with C8 symmetry applied, number of final extra passes 5, and optimize per-particle defocus true.

The map was further improved with additional CTF and motion correction refinement. Exposure Group Utilities was used to divide the micrographs into 37 groups, followed by Global CTF Refinement with number of iterations 2 and Fit spherical aberration true. Non-Uniform Refinement was used to assess the Global CTF Refinement job. The resulting Non-Uniform Refinement job increasing the resolution to 3.18 Å with settings C8 symmetry applied, number of extra final passes 5, and optimize per-particle defocus true. The 3.18-Å map and a Reference Motion Correction job were used to improve the motion correction of all 67,001 particles. Two Non-Uniform Refinement jobs were run of the final motion-corrected particles, with D8 and C1 symmetry applied and number of final extra passes 5 and optimize per-particle defocus true. The resolution of the final D8 map was estimated at 2.95 Å, and the final C1 map was resolved to an estimated 3.34 Å, as evaluated by gold-standard Fourier shell correlation (GSFSC) using a CC1/2 cutoff of 0.143. Both maps were sharpened with DeepEMhancer (67) and assessed further with Local Resolution and Orientation Diagnostics jobs.

For each of the asymmetric and symmetric models, the computationally predicted model was first rigidly docked into the highest-resolution sharpened global refinement map and relaxed using ISOLDE (68) in UCSF ChimeraX (66) with default parameters. Chains and residues were manually positioned as needed to fit into density. The map and model were then imported into Phenix (69), where real-space refinement was run with 1 macrocycle and secondary structure restraint turned off. Afterward, Coot (70) was used to manually refine regions of suboptimal density. ISOLDE (68), Phenix (69), and Coot (70) were then iteratively used to further refine the model. The wwPDB validation service (71) was used periodically during refinement to assess the model’s metrics.

## Supplementary Material

Supplementary Material

## Figures and Tables

**Fig. 1.**
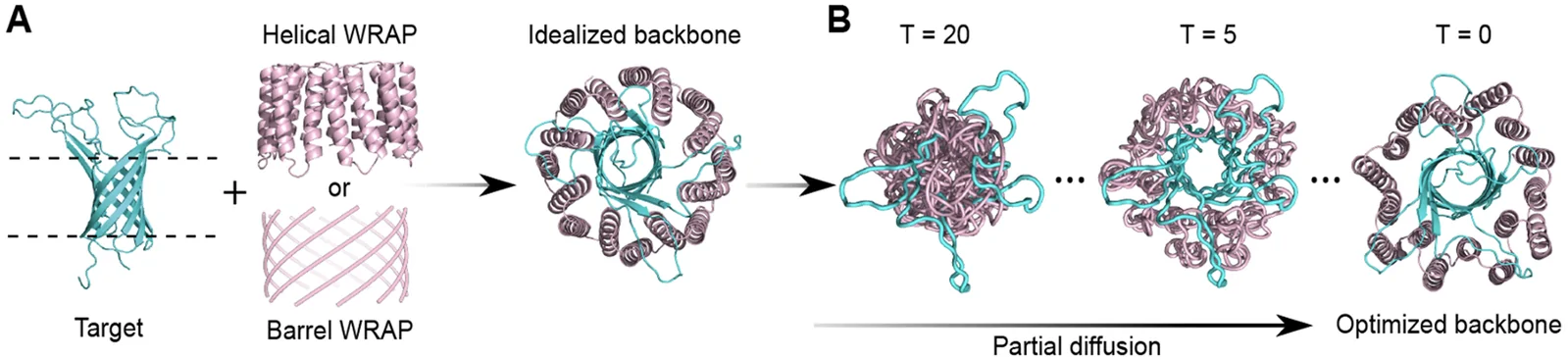
Pipeline for de novo design of WRAPs. (**A**) Protein placement and backbone integration. Target membrane proteins (e.g., *E. coli* OmpA, cyan) were centrally positioned within WRAP backbones (pink) generated through fold-conditioned RFdiffusion (for helical structures) or parametric design (for beta-barrel structures). An idealized backbone, comprising WRAP as chain A and the target protein as chain B, was chosen as the starting point for subsequent refinement steps. (**B**) Backbone refinement. Partial diffusion over 20 time steps (T) was applied to optimize shape complementarity with the target, followed by sequence design using SolubleMPNN and AF2 structure prediction.

**Fig. 2.**
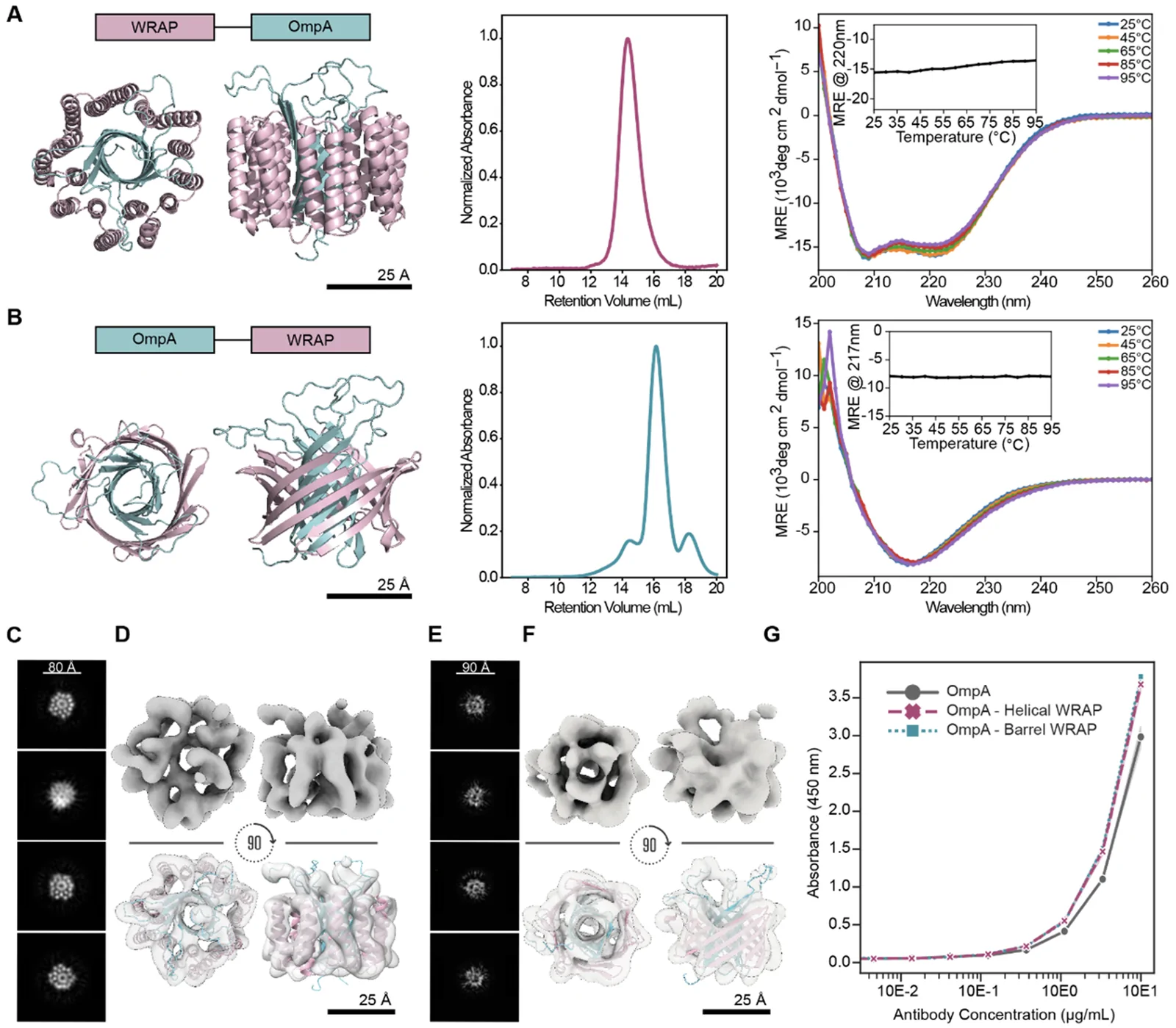
Solubilization of OmpA using helical and beta-barrel WRAPs **(A** and **B)** Left: Design models showcasing (in pink) helical-(A) and beta barrel-(B) WRAPed OmpA [cyan, PDB:2GE4 (19)] designs. Middle: SEC traces of purified protein from soluble fraction. Right: CD data validating the thermal stability of WRAPed OmpA. (**C** and **D**) Representative 2D class averages (C) and Cryo-EM 3D data for helical WRAP-OmpA (D) (61.2 kDa). (**E** and **F**) Representative 2D class averages (E) and cryo-EM 3D data for barrel WRAP-OmpA (F) (41.4 kDa) WRAP-OmpA. The computational designs rigid-body docked into the corresponding density maps showcasing high agreement to the designs (D and F). (**G**) ELISA results confirming the binding of polyclonal OmpA antibody to the helical (pink) and barrel (blue) WRAPed OmpA, in comparison to detergent-purified and amphipol reconstituted OmpA (gray).

**Fig. 3.**
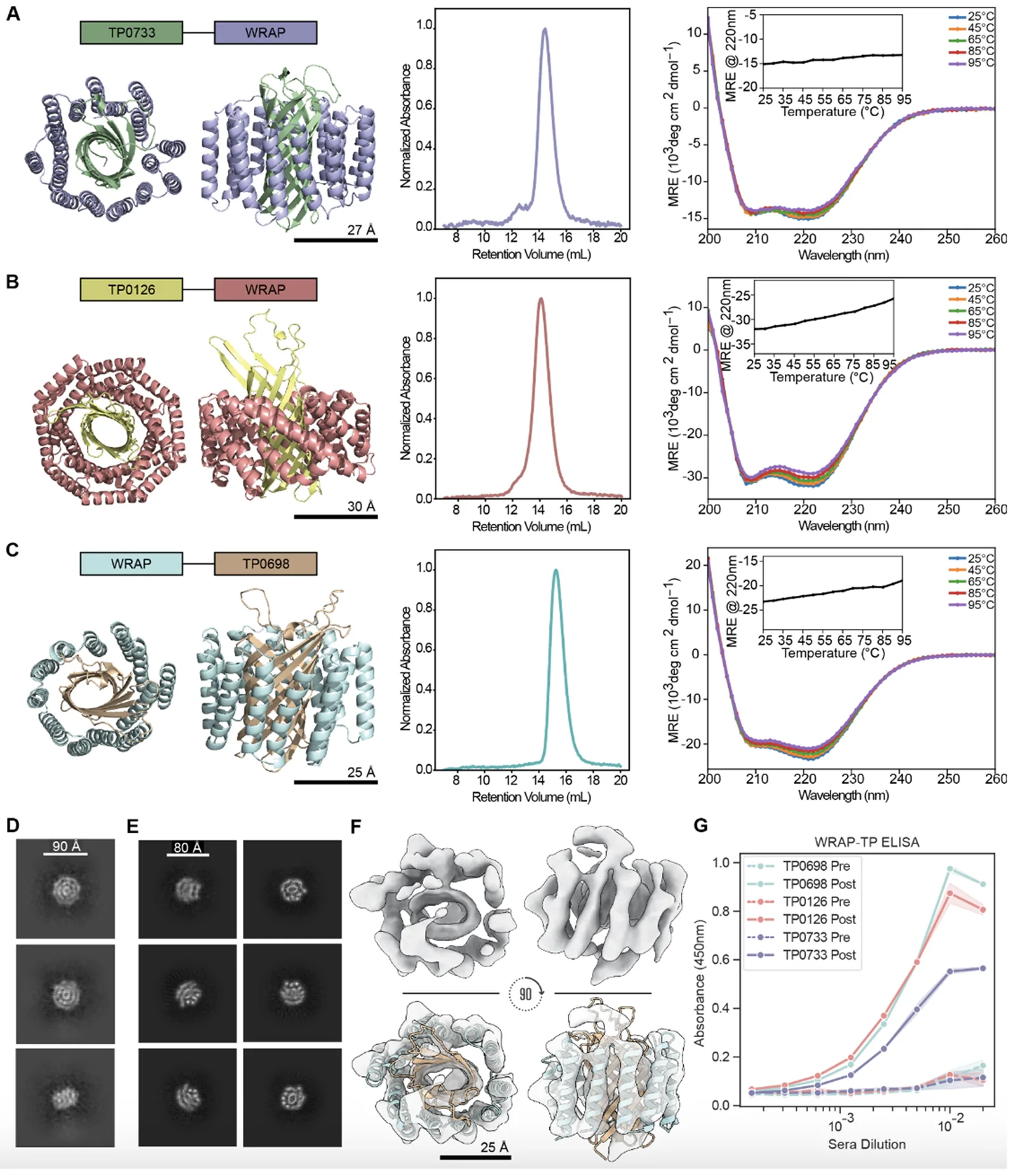
Solubilization of *Tp* OMPs. **(A** to **C)** Left: Design models of TP0733 (A), TP0126 (B), and TP0698-WRAP (C). Middle: SEC traces of purified WRAPed TPs. Right: corresponding CD spectra demonstrating thermostability. (**D**) Representative 2D class averages of TP0126-WRAP particles (72.1 kDa). (**E**) Representative 2D class averages of WRAP-TP0698 particles (49.4 kDa). (**F**) 3D cryo-EM density map of WRAP-TP0698 with the corresponding computational design rigid body docked into the map, highlighting agreement in overall size and shape between the experimental density and design model. (**G**) ELISA results showing response against WRAPed TP0698, TP0126, and TP0733 with pooled serum from rabbits infected with live *Tp* (Post) compared with the same unvaccinated animals (Pre).

**Fig. 4.**
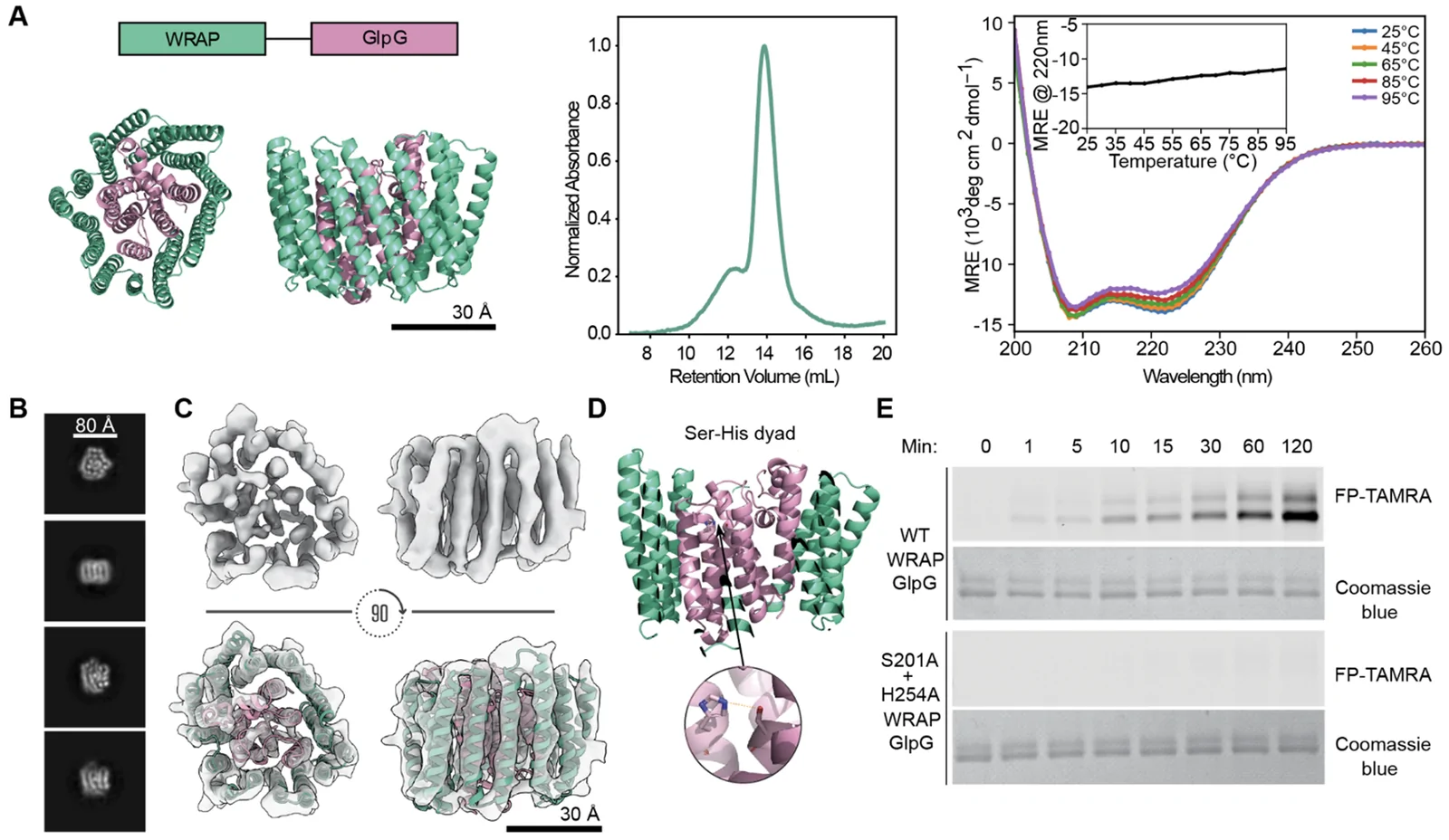
WRAPed intramembrane protease GlpG maintains activity. **(A)** Left: Design model of WRAP (green)-GlpG [magenta, PDB ID: 2IC8 (28)]. Middle: SEC trace of protein purified from the soluble fraction of E. coli lysates. Right: CD data showing that WRAP-GlpG is thermostable. (**B**) Representative 2D class averages of WRAP-GlpG particles (74.3 kDa). (**C**) Cryo-EM 3D density map and computational design model rigid body docked into the density map, revealing high agreement. (**D**) Shown is the catalytic serine-histidine dyad of WRAPed GlpG. (**E**) Time-dependent FP-TAMRA active-probe labeling of WT WRAP-GlpG compared with the catalytic mutant. Coomassie blue staining shows the total protein content in each reaction. Double-band migration was sometimes observed on SDS gels, but GlpG proteins run as a predominantly single band on the native gel (fig. S15).

**Fig. 5.**
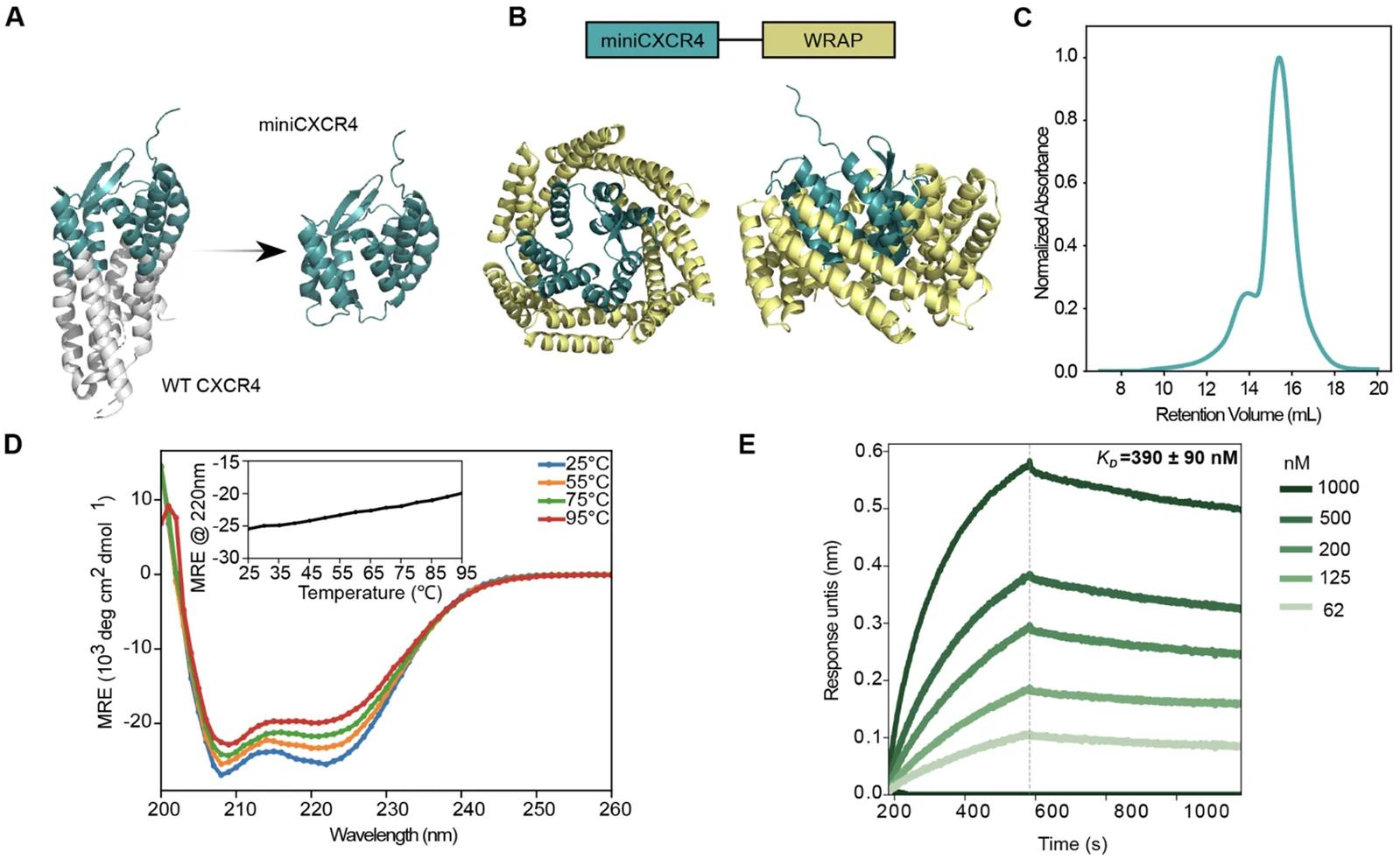
miniCXCR4-WRAP binds to the native ligand CXCL12. **(A)** Wild-type CXCR4 [PDB ID: 4RWS (32)] was truncated right below the ligand-binding pocket (deep teal). (**B**) miniCXCR4 (deep teal)–WRAP (yellow) design model. (**C**) SEC trace of purified protein from the soluble fraction of E. coli lysates. (**D**) CD spectra demonstrating that miniCXCR4-WRAP is thermostable. (**E**) BLI data showing binding of miniCXCR4-WRAP to CXCL12 with K_D_ = 390 ± 90 nM.

**Fig. 6.**
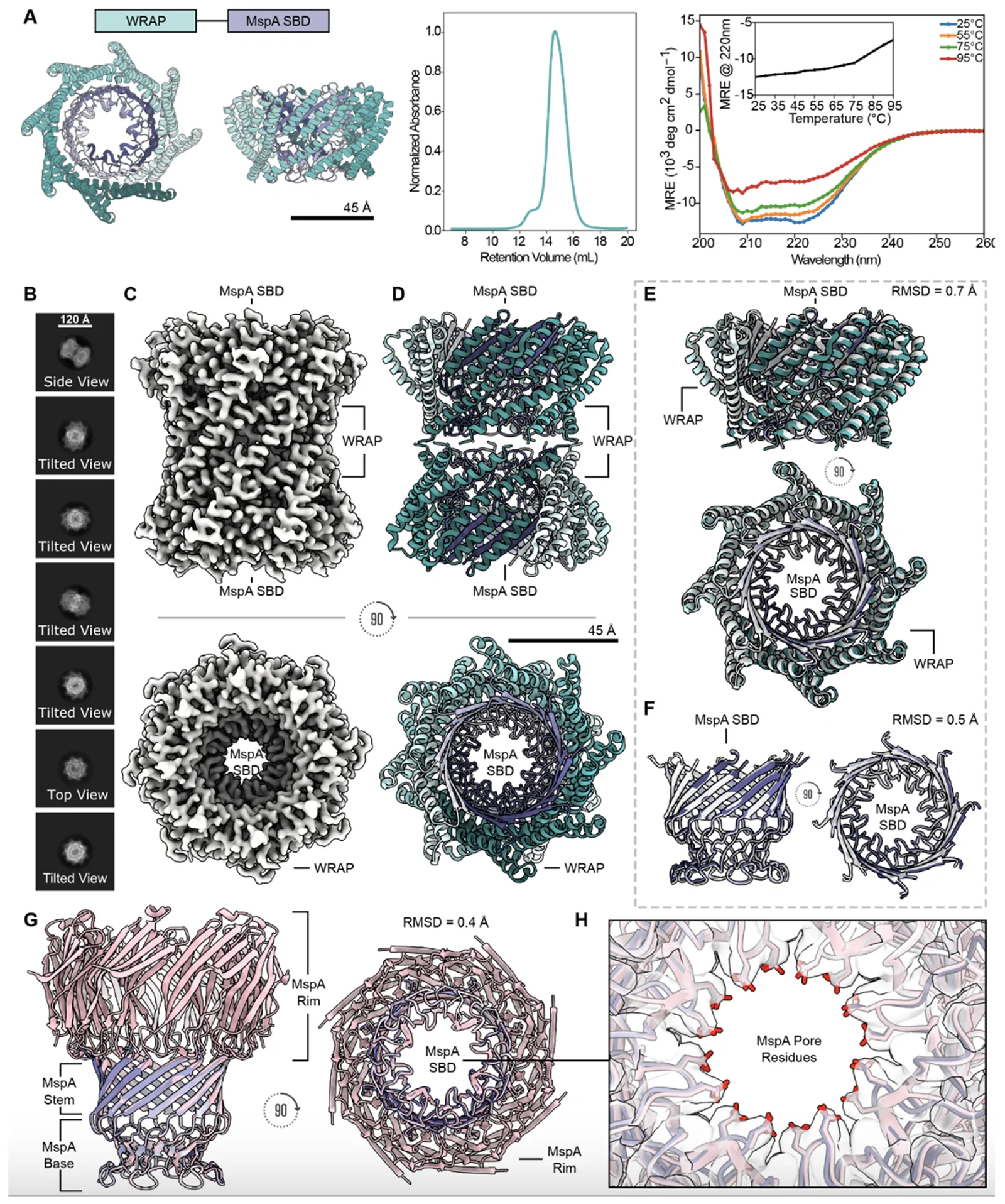
High-resolution cryo-EM structure of the WRAP-MspA_SBD. **(A)** Left: WRAP-MspA_SBD [MspA PDB ID: 1UUN (39)] design model consisting of the WRAP oligomerization domain fused to the TMD of MspA. Middle: SEC trace showing a slightly broad but well-defined peak consistent with assembled WRAP-MspA_SBD complexes. Right: CD spectroscopy confirming that WRAP-MspA_SBD is folded and thermally stable, maintaining the secondary structure up to 75°C. (**B**) Representative 2D class averages of WRAP-MspA_SBD particles reveal clearly defined secondary structure in top, side, and tilted views. The particles exhibit C8 symmetry along the major axis and dimerization along the minor axis between C8 oligomers. (**C**) Cryo-EM density map of WRAP-MspA_SBD (136.7 kDa) at 2.9-Å resolution refined with D8 symmetry and displayed along two orthogonal view axes. (**D**) Atomic model built into the cryo-EM density shown in (C), displayed along two orthogonal view axes. (**E**) Structural alignment of the C8 component of the cryo-EM structure (teal-purple) with the corresponding computational design model (gray), with views shown along two orthogonal axes (RMSD = 0.7 Å). (**F**) Comparison of only the MspA TMD from the cryo-EM structure and design model, shown along two orthogonal axes, revealing close agreement (RMSD = 0.5 Å). (**G**) Overlay of the WRAP-MspA_SBD cryo-EM structure (with the WRAP domain hidden), in purple and the previously reported crystal structure of full-length MspA [PDB ID: 1UUN (39)] in pink shown along two orthogonal axes to illustrate preservation of the native transmembrane architecture (RMSD = 0.4 Å). (b) Close-up view of the DNA-sensing region of the MspA TMD, highlighting preservation of key side-chain rotamers involved in function.
