# Supplementary material for: Membrane protein solubilization and structure determination using de novo–designed proteins
Source: Science. Author manuscript; Available in PMC 2026 Nov 3. (PMC13730216; doi:10.1126/science.adr3817)
Supplement: Supplementary file 1 [file NIHMS2216622-supplement-Supplementary_Material.pdf]

Supplementary Materials for

Membrane protein solubilization and structure determination using de novo-designed proteins

Authors: Ljubica Mihaljević[1,7]†, David E. Kim[1,7]†, Pooja D. Bandawane[1]†, Helen E. Eisenach[1], Andrew J. Borst[1], Alexis Courbet[1,7,8], Connor Weidle[1], Kenneth D. Carr[1], Everton Bettin[2,3,4], Qiushi Liu[9], Aldo T. Trejos[12,13], Sagardip Majumder[1], Surabhi Kokane[14], Alexander Stevens[14], Edin Muratspahić[1], Thomas Schlichthaerle[1], Marc Expòsit[1], Xinting Li[1], Mila Lamb[1], Analisa Nicole Azcárraga Murray[1], Rashmi Ravichandran[1], Elizabeth C. Williams[1], Shuyuan Hu[1], Lynda Stuart[1], Linda Grillová[10], Nicholas R. Thomson[10,11], Michael Landreh[14,15], Pengxiang Chang[9], Lorenzo Giacani[12,13], Melissa J. Caimano[2,3,4], Kelly L. Hawley[2,3,5,6], Neil P. King[1], David Baker[1,7,*]

Corresponding author: dabaker@uw.edu

The PDF file includes:

Figures S1 to S15
Tables S1 to S5
References 72-76

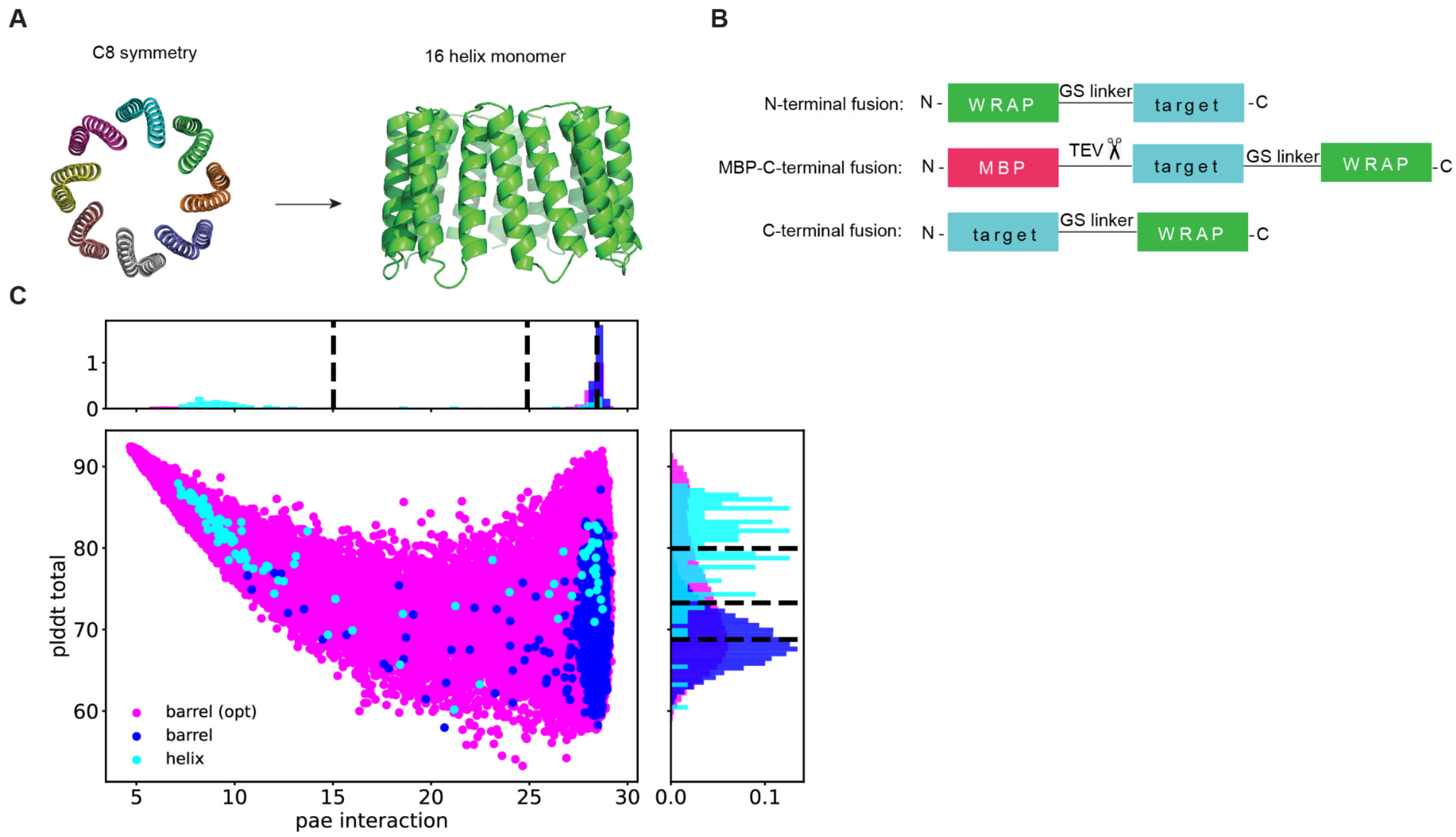

Fig. S1. (A) Fold-conditioned RFdiffusion was used to generate symmetric C8 oligomers, which were then connected into a single monomeric 16-helical chain using RFdiffusion. The resulting chain was used to place the target protein in the center using PyMOL and the complex was optimized using partial diffusion. **(B)** Evaluation of several single-gene WRAP–membrane protein fusion strategies demonstrated that all three configurations can successfully produce soluble membrane protein targets. **(C)** Significantly more sampling was required to generate barrel WRAPs (blue) with AF2 pLDDT total and PAE interaction scores that satisfied the selection criteria compared to helix WRAPs (cyan). An iterative optimization strategy (see Materials and Methods in manuscript) was used to generate barrel WRAPs (magenta) with optimal AF2 scores. Normalized distributions of PAE interaction and pLDDT total scores are shown in the top and right panels, respectively; mean values are indicated with broken lines.

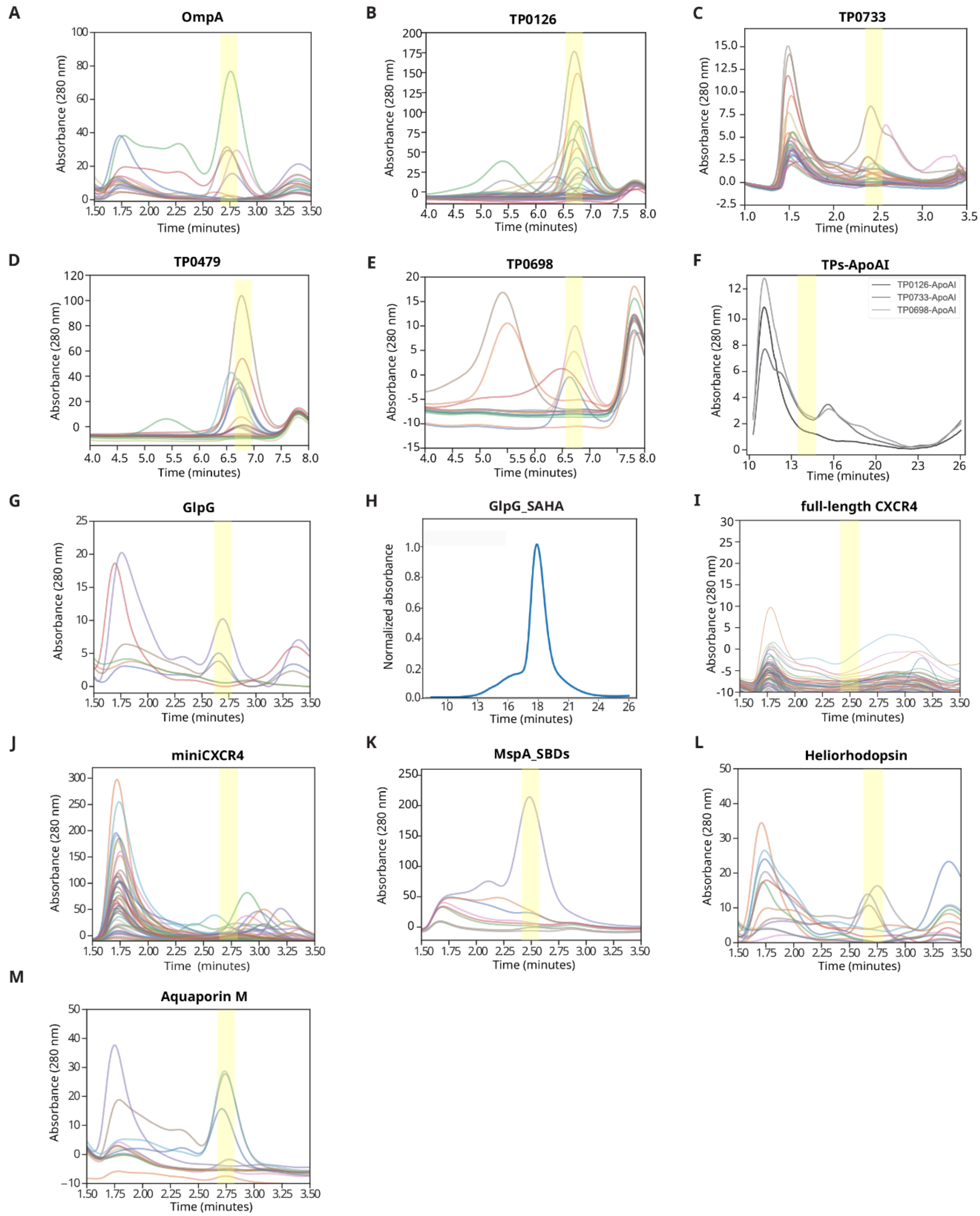

Fig. S2. Eluted proteins from soluble fraction. Expression screen from a 4 mL 96-well purification of WRAPed designs using HPLC (with success rates in brackets) **(A)** OmpA (5 out of 6 for helical and 1 out of 6 for barrel WRAP), **(B)** TP0126 (19 out of 58), **(C)** TP0733 (3 out of 10), **(D)** TP0479, (4 out of 24) **(E)** TP0698 (7 out of 29). **(F)** SEC traces of TP0126, TP0733

and TP0698 with ApoAI **(G)** GlpG HPLC trace (3 out of 6). **(H)** SEC trace of S201A+H254A GlpG catalytic mutant. HPLC traces of **(I)** full-length CXCR4 (0 out of 49) **(J)** miniCXCR4 (2 out of 46)**,** (**K**) MspA_SBDs (1 out of 8), (**L**) Heliorhodopsin (PDB: 6IS6 (*72*) 3 out of 6) and (**M**) Aquaporin M (PDB: 2EVU (*73*)) 3 out of 6). These purification profiles (except H) are from a polyclonal small-scale expression screen of proteins of different sizes and the yellow line approximately indicates the expected elution based on molecular weight. Designs with the highest yield, minimal aggregation and correct predicted elution size were selected for further characterization.

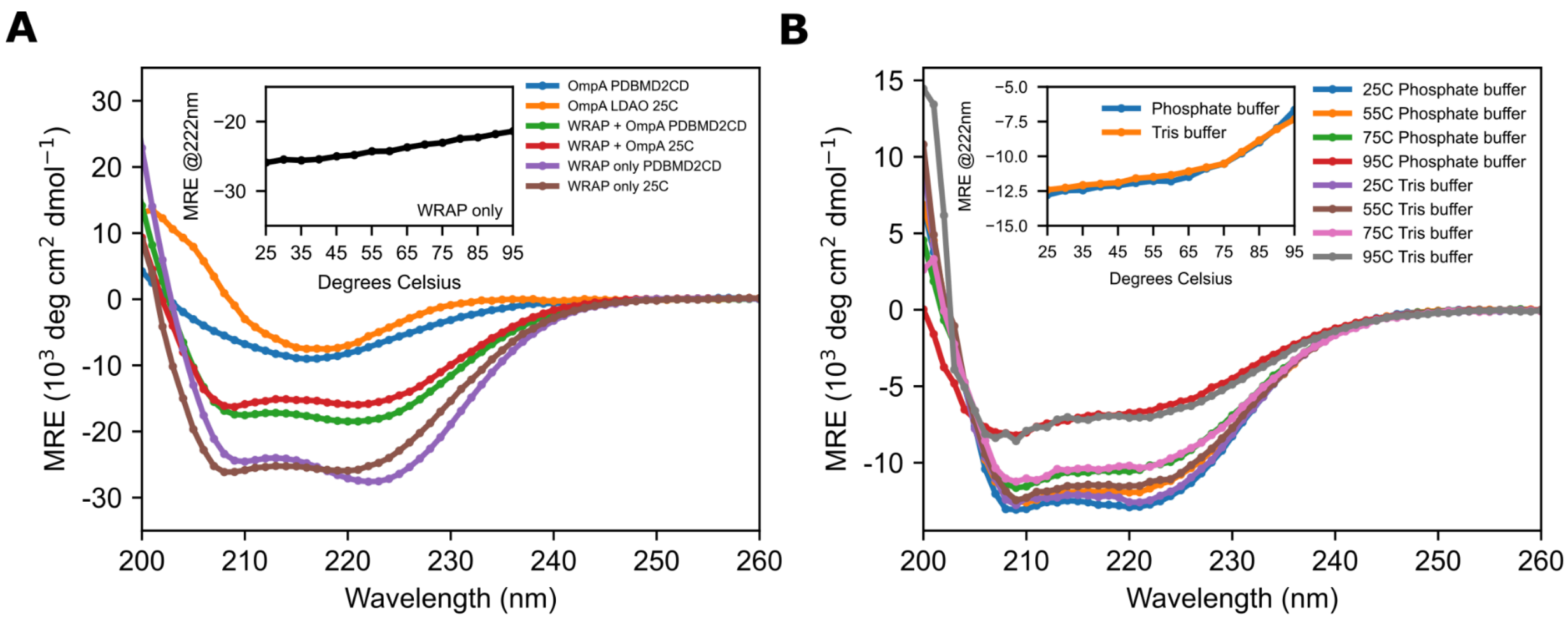

Fig. S3. CD spectra and thermal denaturation profiles. (A) The far-UV CD spectra of OmpA in LDAO (lauryldimethylamine N-oxide) detergent, helical WRAPed OmpA, and helical 'WRAP only' correspond with PDBMD2CD (*74*) predicted spectra. The OmpA helical 'WRAP only' maintains helical structure at 25-95°C as indicated by its α-helical CD spectra with minima at ~208 and 222 nm and temperature melt profile (inset). The CD spectra of the WRAPed OmpA complex is consistent with the PDBMD2CD predicted spectra representing the combined contribution of secondary structure from OmpA (beta-sheet) and the WRAP only (α-helix). **(B)** WRAPed MspA_SBD in Tris and phosphate buffers. Because Tris–HCl exhibits reduced buffering capacity and stability at elevated temperatures, CD measurements were additionally performed in 20 mM phosphate buffer (100 mM sodium sulfate, pH 8.0). The far-UV CD spectra and the temperature-dependent CD signal at 222 nm (inset) show close agreement between the two buffer conditions. The temperature melt profiles are also consistent with the DSF and SLS thermal melt data for both buffer conditions (Figure S4D and E).

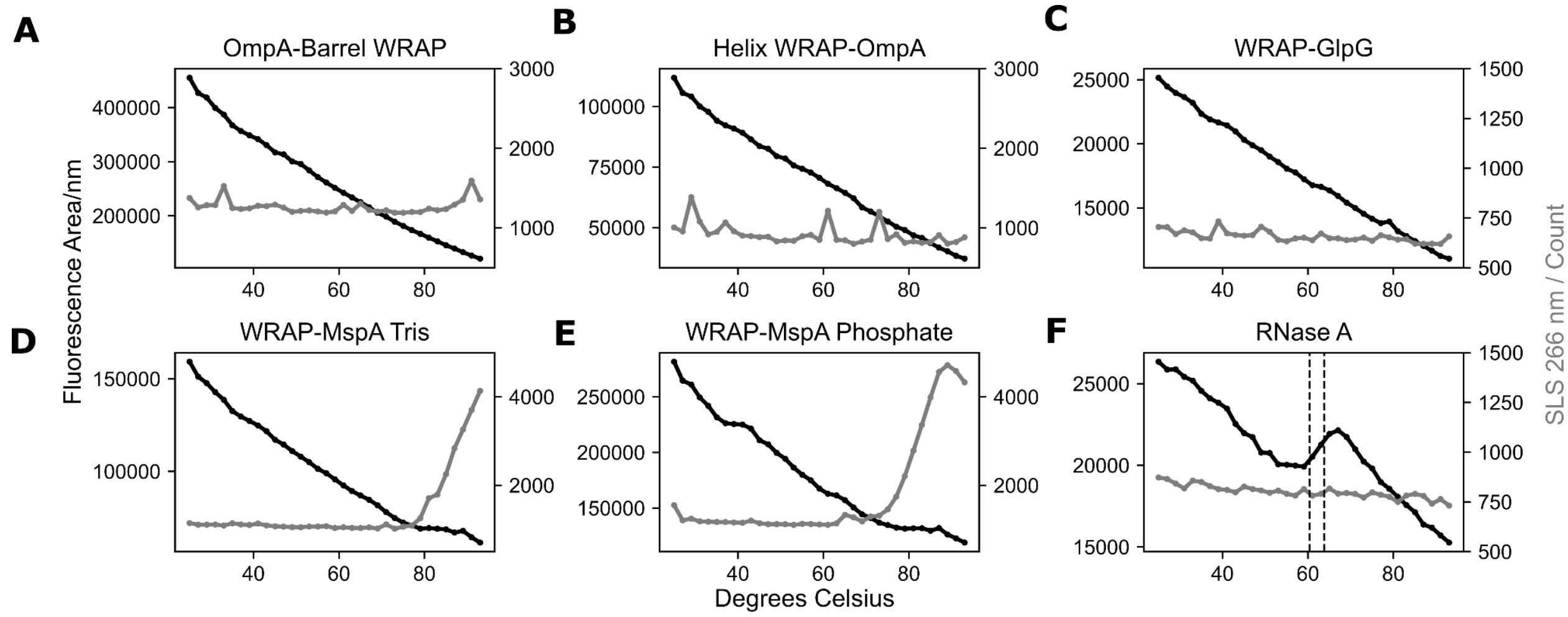

Fig. S4. Thermal stability of representative WRAPed targets measured by DSF and SLS. OmpA with **(A)** β-barrel and **(B)** α-helical WRAP, and **(C)** WRAPed GlpG show minimal changes to the baseline fluorescence emission and SLS intensity across 25–93°C, indicating thermal stability and an absence of aggregation over the experimental timescale. WRAPed MspA_SBD in **(D)** Tris and **(E)** phosphate buffers begins to unfold and aggregate at ~75°C, as indicated by increased SLS intensity and corresponding fluorescence transitions. These results are consistent with the CD spectra and thermal melt data presented in the main text. For reference, RNase A **(F)** was also analyzed, and the observed fluorescence transition temperature agrees with previously reported melting temperatures (60.4 and 63.8°C; dashed vertical lines; (*75*, *76*)).

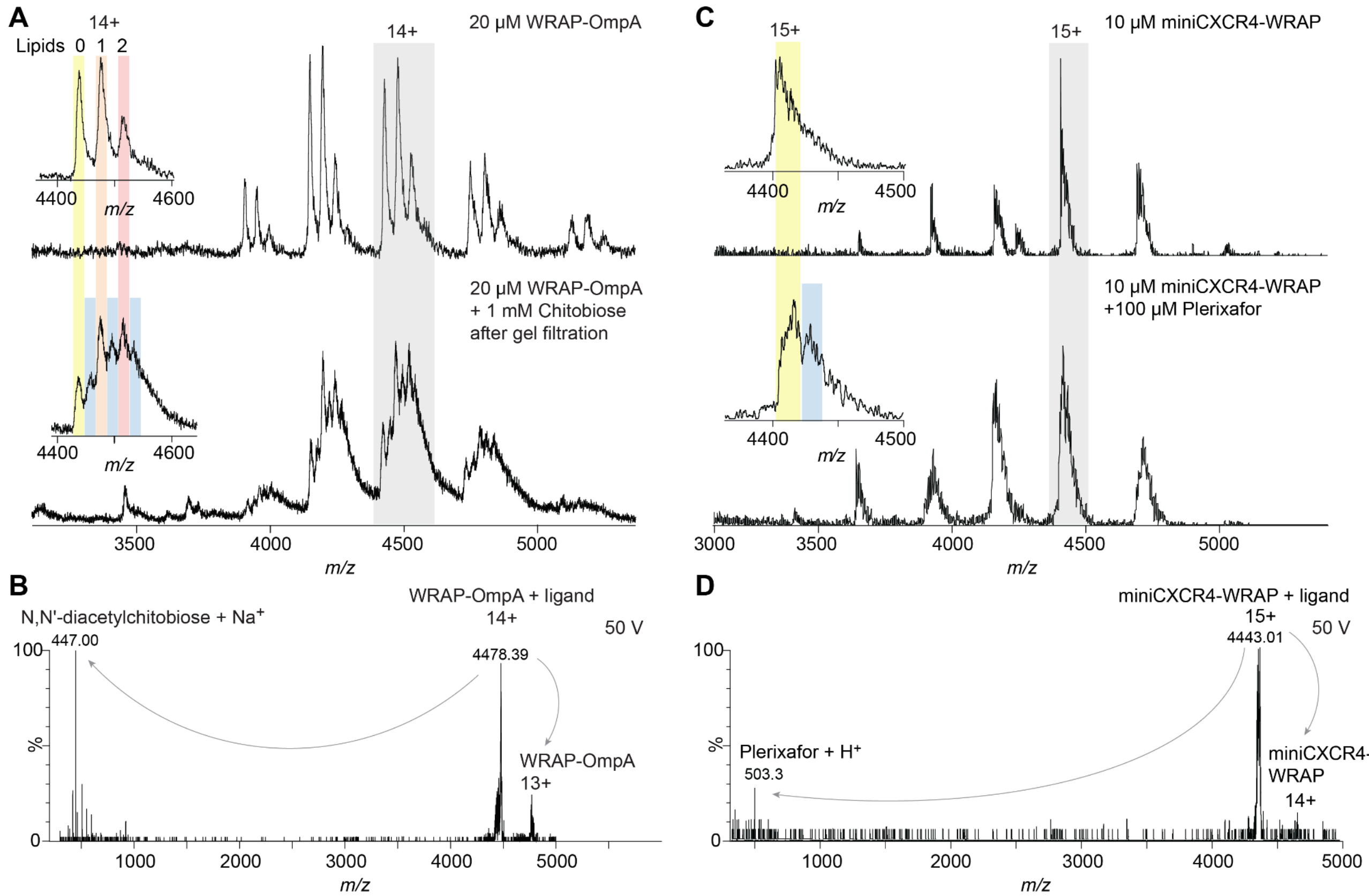

Fig. S5. Native mass spectrometry reveals ligand binding to WRAPed membrane proteins. **(A)** Native mass spectra of WRAP–OmpA show co-purified membrane lipids (top) and binding of up to three chitobiose molecules (bottom, highlighted in blue). **(B)** MS/MS analysis of the 14^+ charge state of the complex results in release of the non-covalently bound chitobiose ligand. **(C)** Native mass spectra of miniCXCR4-WRAP show additional peaks upon addition of plerixafor, consistent with formation of a 1:1 protein–drug complex (highlighted in blue). **(D)** Drug binding was confirmed by MS/MS analysis of the 15^+ charge state of the complex.

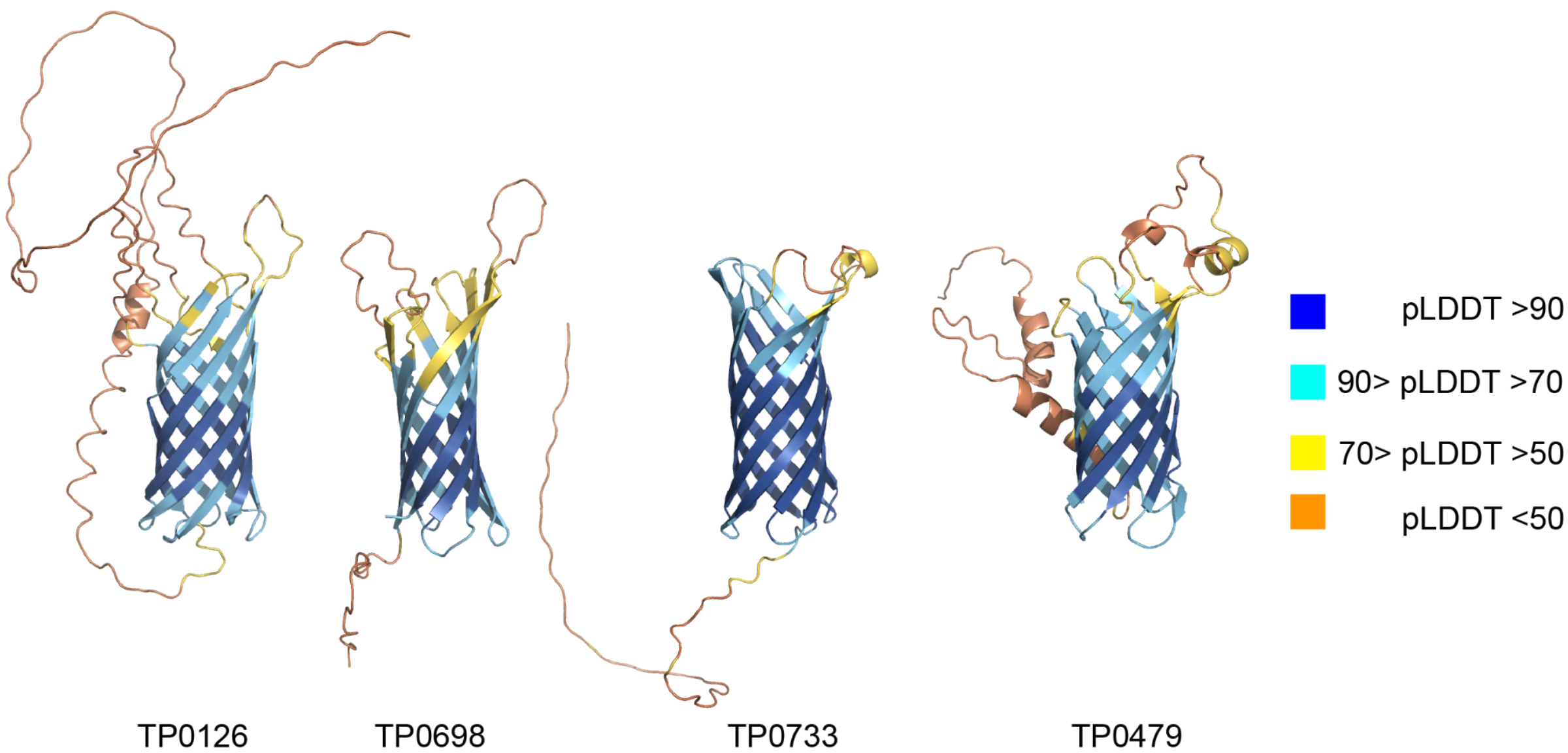

Fig. S6. AF2-predicted TP OMPs. TP0126, TP0698, TP0733 and TP0479 models colored by AF2 confidence sore (pLDDT), with high confidence in dark blue and low in orange. Intracellular unstructured loops have been removed (see Table S2 for the exact sequence) due to poor prediction and to reduce the size and complexity during the design.

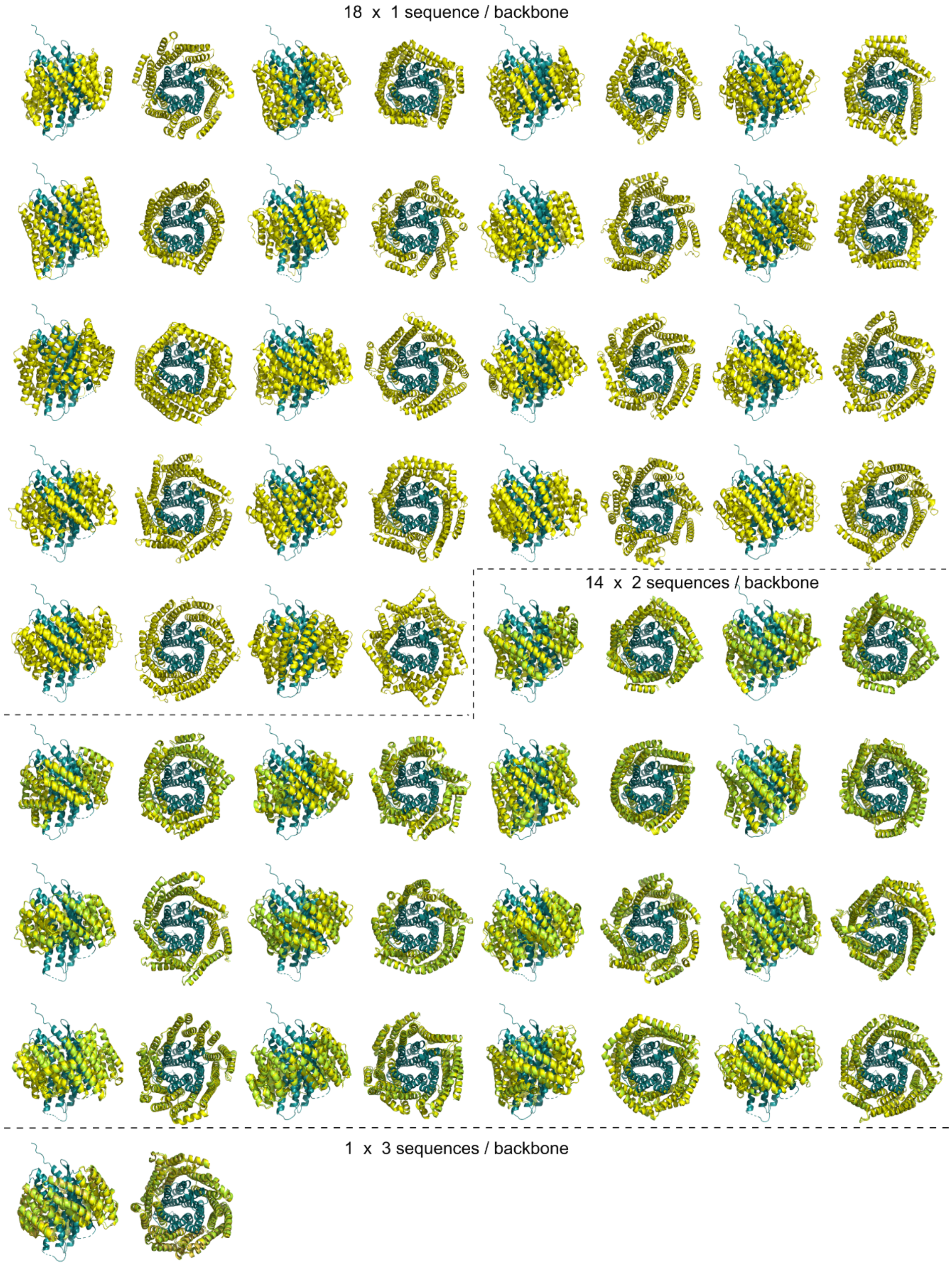

Fig. S7. Backbone diversity of selected full length CXCR4 WRAPs. A total of 49 designs with 33 unique backbone architectures (side and top view) were selected, each annotated with the number of sequences designed for the given backbone (×1 or ×2 or ×3).

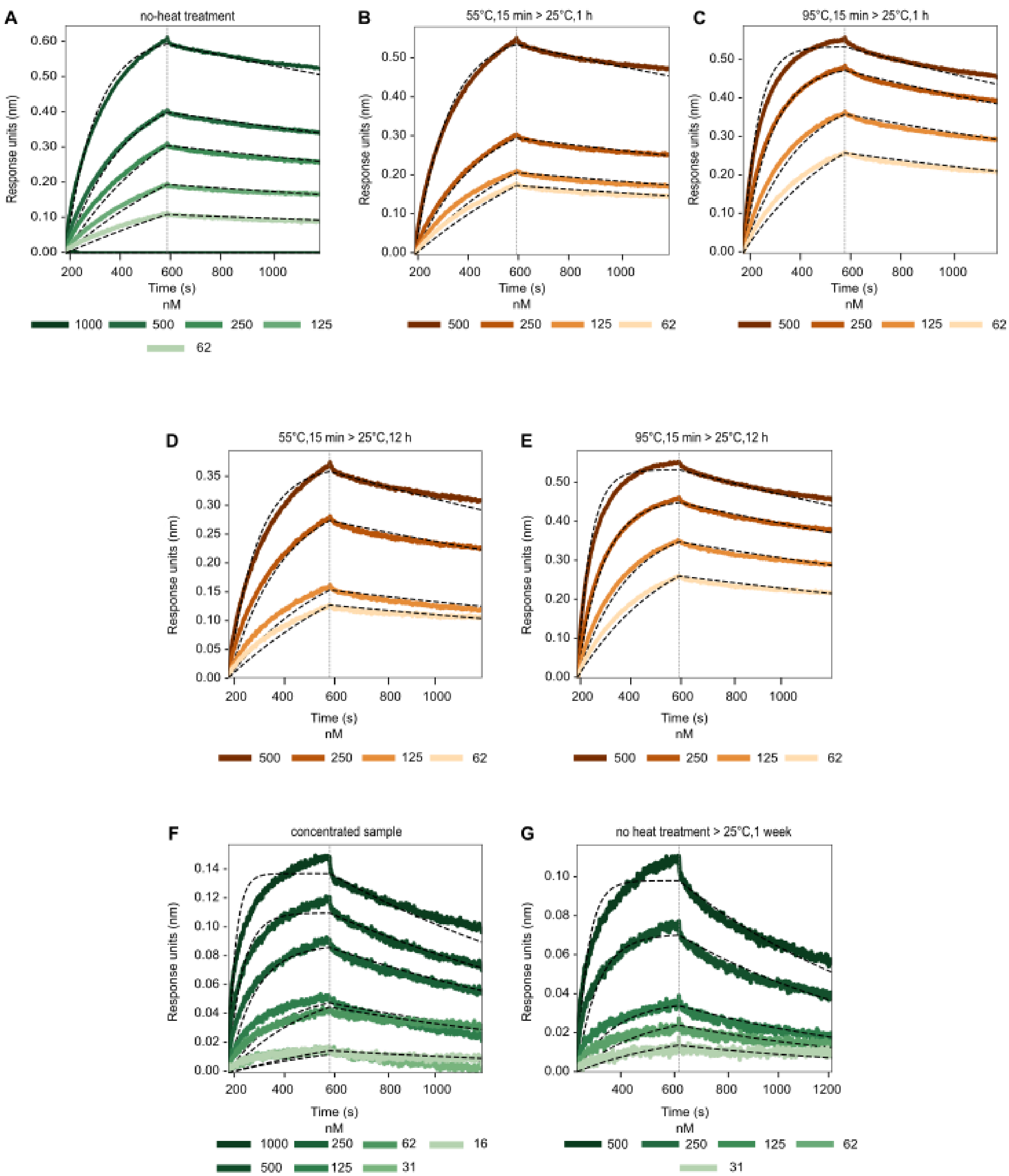

Fig. S8. Bio-layer interferometry (BLI) analysis of CXCL12 binding to miniCXCR4-WRAP performed at 25°C after different thermal treatment and incubation conditions. Steady-state equilibrium dissociation constants (K_D) were obtained from equilibrium binding responses for following conditions (**A**) no pre-treatment, K_D of 390±90 nM , (**B**) heating at 55°C for 15 minutes followed by incubation at 25°C for 1 hour, K_D of 230±84 nM (**C**) heating at 95°C for 15 minutes followed by incubation at 25°C for 1 hour, K_D of 53±20 nM. (**D**) heating at 55°C

for 15 minutes followed by longer incubation at 25°C for 12 hours, K_D of 290±51 nM. (**E**) heating at 95°C for 15 minutes followed by longer incubation at 25°C for 12 hours, K_D of 67±15 nM. (**F**) Concentrated WRAP-miniCXCR4 sample without heat treatment, K_D of 280±36 nM (**G**) No heat treatment followed by incubation at 25°C for 1 week K_D of 530±110 nM. Kinetic parameters were derived from global fits to a 1:1 binding model, yielding following K_D, 31.9±0.38 nM, 18.2±0.23 nM, 9.71±0.11 nM, 20.1±0.29 nM, 8.24±0.08 nM, 20.3±0.25 nM, and 24.6±0.29 nM for conditions **A–G**, respectively (detailed in Table S5). Because kinetic and steady-state estimates were not fully concordant, steady-state K_D values are reported as the primary measure of binding affinity.

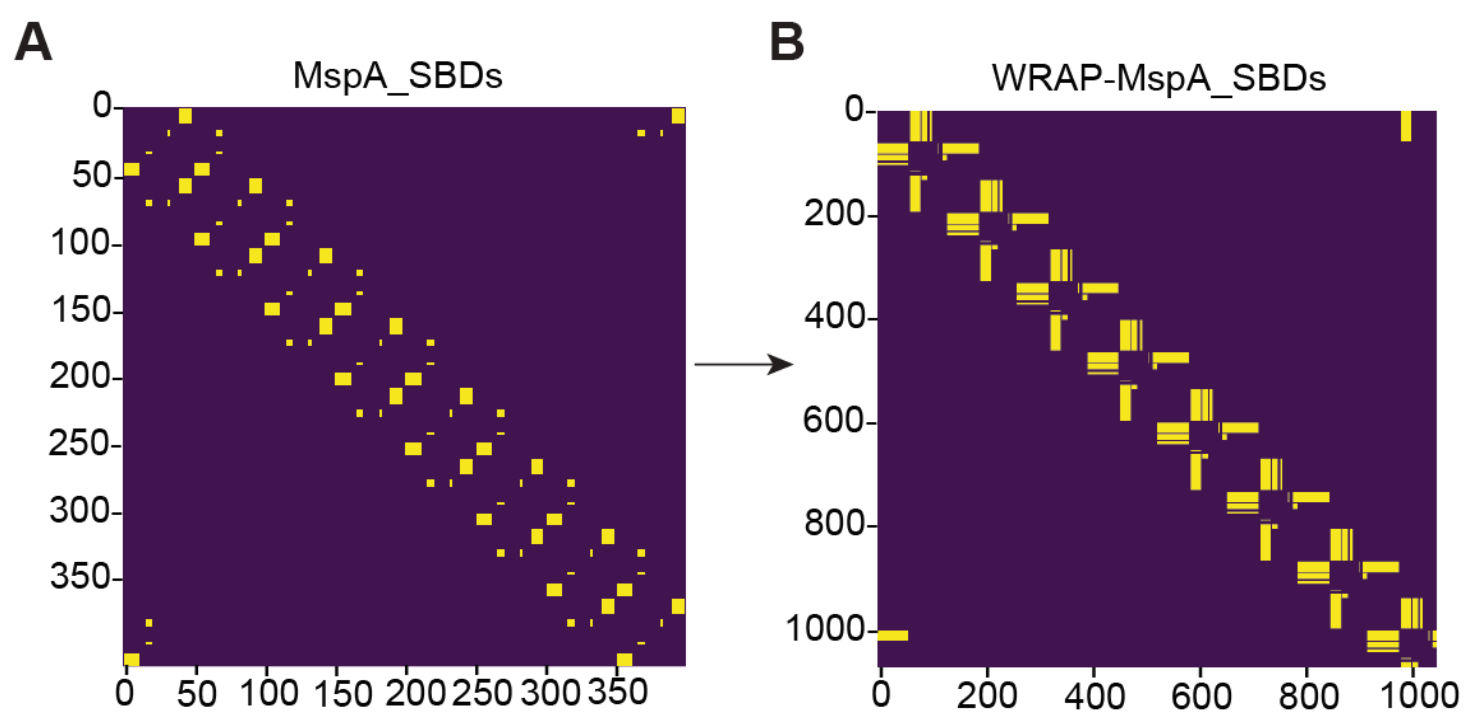

Fig. S9. MspA adjacency matrix of (A) Native MspA without N- and C-terminus (MspA_SBDs) and **(B)** after adding WRAP helices to MspA_SBDs.

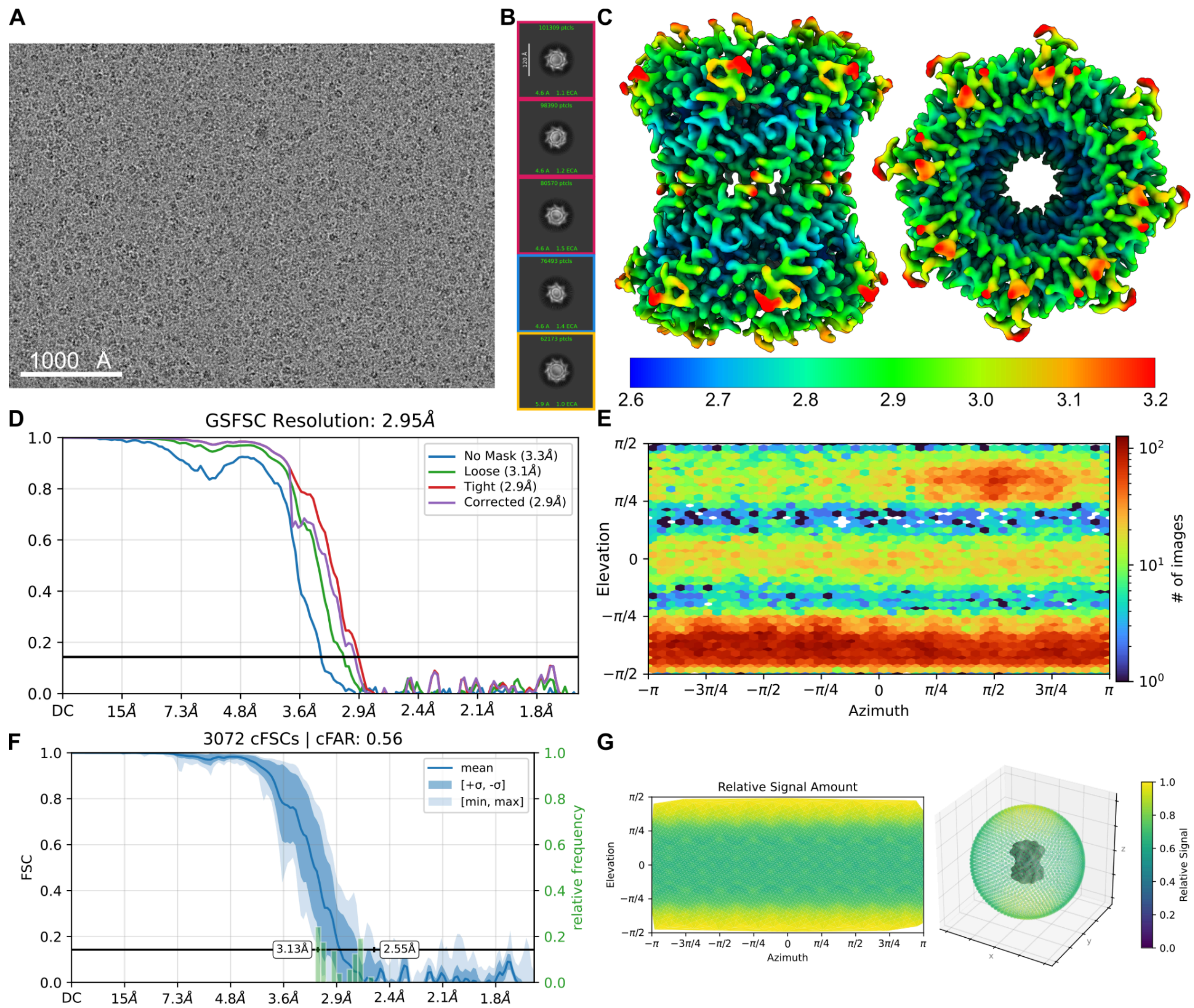

Fig. S10. Cryo-EM analysis of WRAP-MspA_SBDs refined with D8 enforced symmetry. (**A**) Raw Cryo-EM micrograph showing WRAP-MspA_SBDs particles with a 1000 Å scale bar. (**B**) The top 5 classes from an initial 2D classification job containing C8 (pink), C7 (blue), C9 (orange) oligomeric classes. (**C**) Local resolution estimation of the final map sharpened with deepEMhancer. Colors range from 2.6 Å (blue) to 3.2 Å (red). (**D**) Gold-standard fourier shell correlation (GSFSC) plot showing an estimated resolution of 2.95 Å at a cutoff of 0.143. (**E**) View angle distribution plot. (**F**) Conical FSC area ratio plot (**G**) Relative signal plot showing full angular sampling with minimal anisotropy.

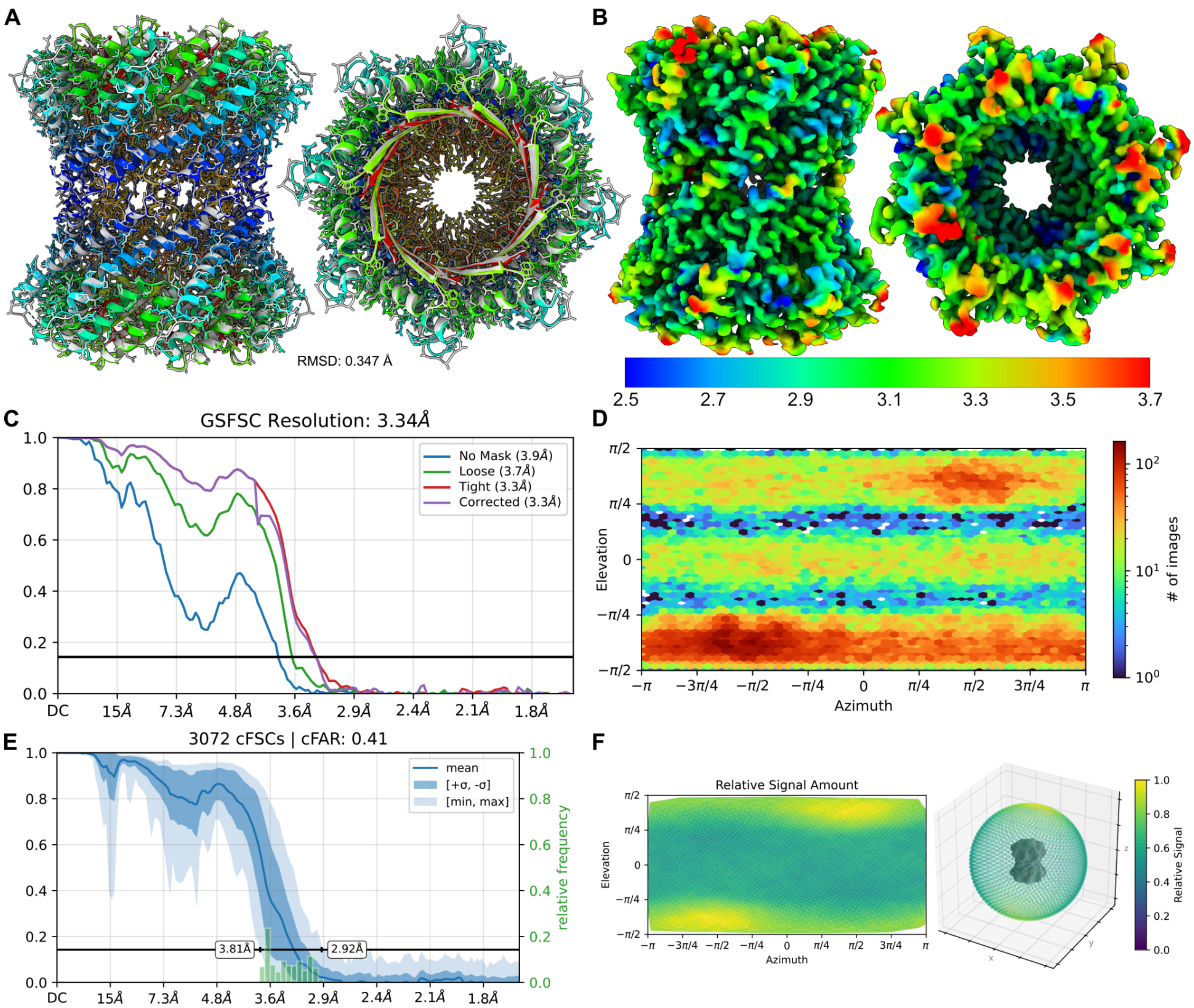

Fig. S11. Cryo-EM analysis of WRAP-MspA_SBDs refined asymmetrically. (**A**) Comparison of Cryo-EM structures determined with and without symmetry operators enforced during 3D map refinement. Asymmetric model (rainbow) and symmetric model (light gray) (RMSD = 0.347 Å). (**B**) Local resolution estimation of the final map sharpened with deepEMhancer. Colors range from 2.5 Å (blue) to 3.7 Å (red). (**C**) GSFSC plot showing an estimated resolution of 3.34 Å at a cutoff of 0.143. (**D**) View angle distribution plot. (**E**) Conical FSC area ratio plot (**F**) Relative signal plot showing full angular sampling with minimal anisotropy.

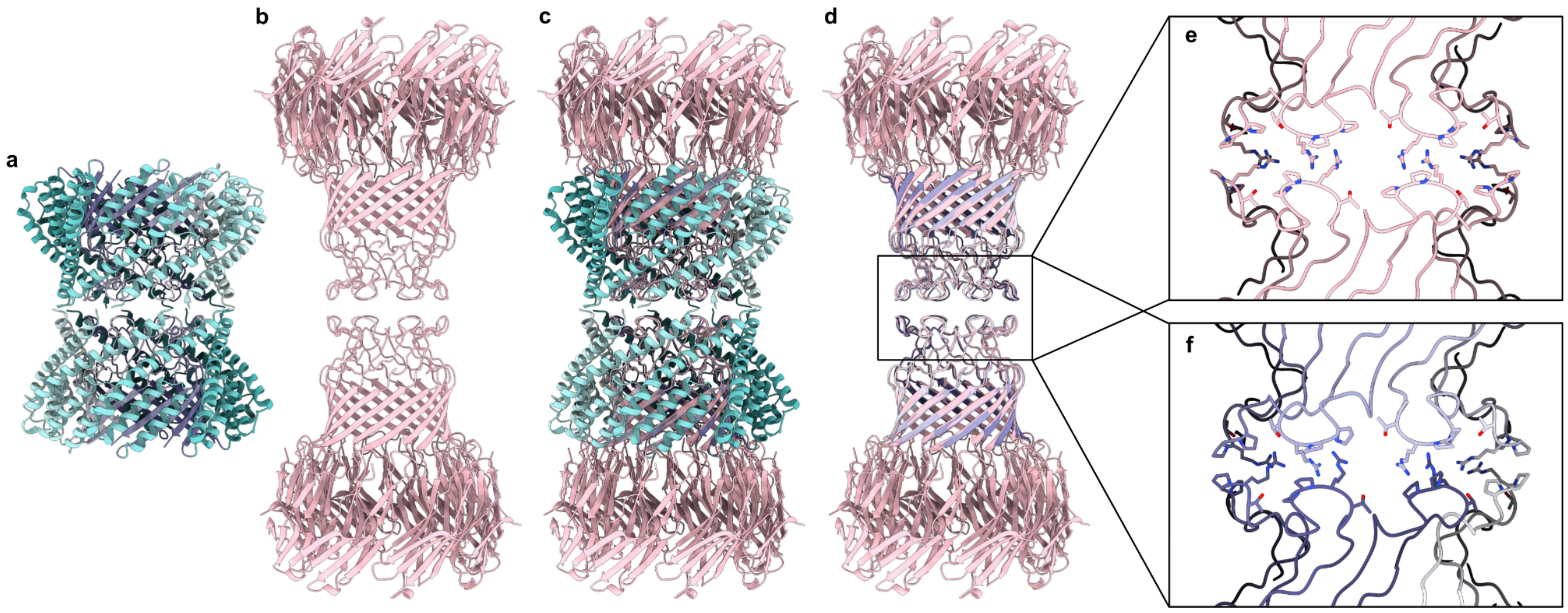

Fig. S12. Dimerization of C8 assemblies is conserved between WRAP-MspA_SBDs and native full-length MspA. (A) Cryo-EM structure of WRAP-MspA_SBDs showing dimerization between two C8 oligomers. **(B)** Crystal structure of full-length native MspA (PDB ID: 1UUN (*38*)) showing a nearly identical C8 dimerization interface, observed along a crystal packing interface. **(C)** Overlay of the cryo-EM structure (teal-purple) and crystal structure (pink) reveals that dimerization occurs at the same interface in both structures. **(D)** Focused overlay of only the MspA_SBDs region from the WRAP-MspA_SBDs cryo-EM structure and the corresponding region in the crystal structure, highlighting near-identical alignment and reinforcing that the dimerization interface is mediated through the native transmembrane domain**. (E–F)** Comparison of interfacial residues involved in dimerization across both structures demonstrates that the interaction is preserved and primarily mediated by the native MspA SBD residues, not the WRAP domain.

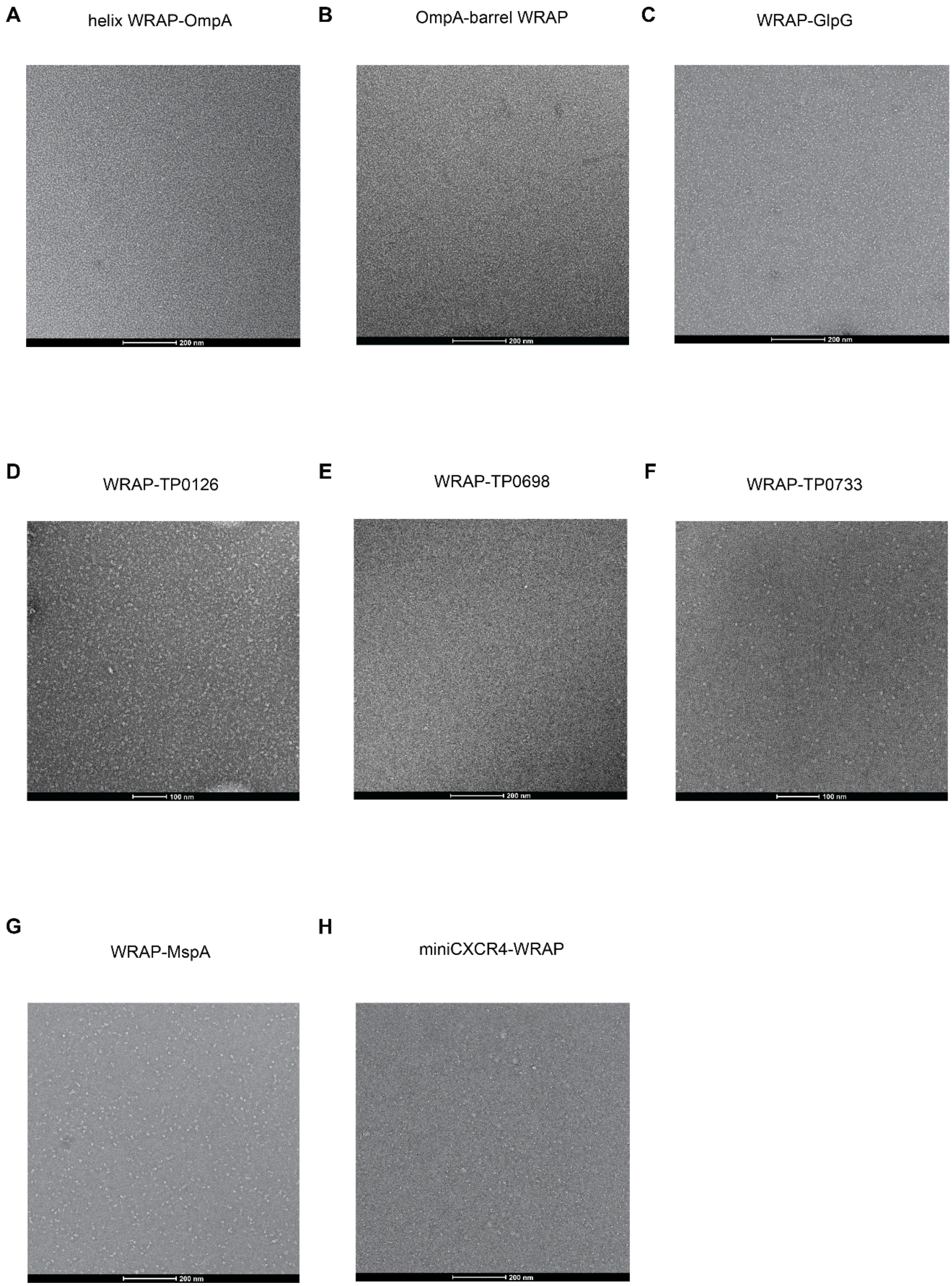
A
helix WRAP-OmpA
B
OmpA-barrel WRAP
C
WRAP-GlpG
D
WRAP-TP0126
E
WRAP-TP0698
F
WRAP-TP0733
G
WRAP-MspA
H
miniCXCR4-WRAP

Fig. S13. Negative stain Electron Microscopy micrographs of **(A)** helix-WRAP-OmpA, **(B)** Barrel-WRAP-OmpA, **(C)** WRAP-GlpG, **(D)** WRAP-TP0126, **(E)** WRAP-TP0698, **(F)** WRAP-TP0733, **(G)** WRAP-MspA and **(H)** miniCXCR4-WRAP.

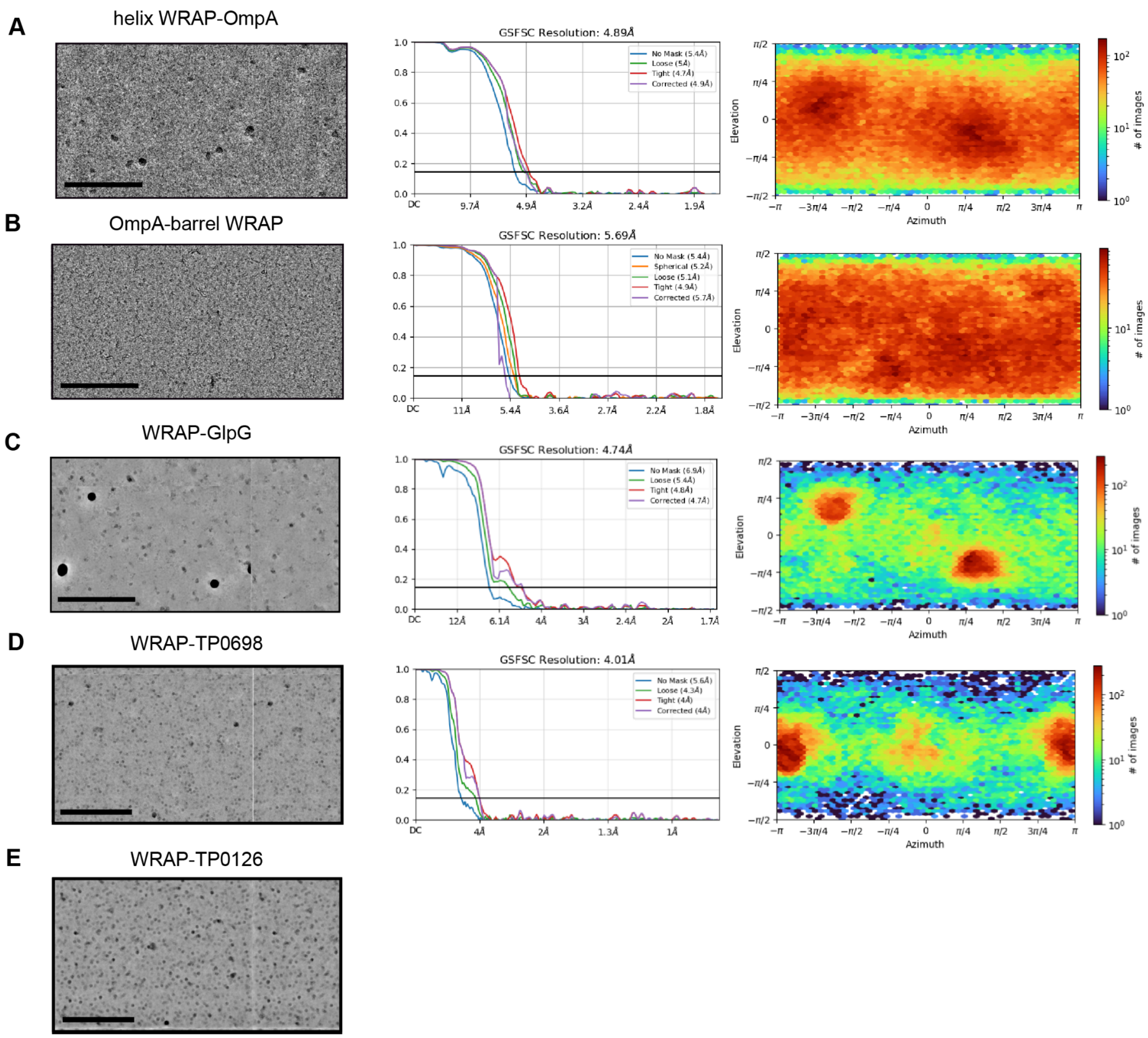

Fig. S14. Additional cryo-EM data collection and processing parameters for smaller WRAP–IMP samples. (A–E) Representative cryo-EM data for five WRAP–IMP constructs: **(A)** helix WRAP–OmpA, **(B)** OmpA–barrel WRAP, **(C)** WRAP–GlpG (denoised), **(D)** WRAP–TP0698 (denoised), and **(E)** WRAP–TP0126 (denoised). For each sample, panels are shown from left to right as follows: representative raw cryo-EM micrographs, gold-standard global Fourier shell correlation (FSC) curves with reported resolutions determined using the FSC = 0.143 criterion, and corresponding particle orientational distribution plots derived from the final reconstructions.

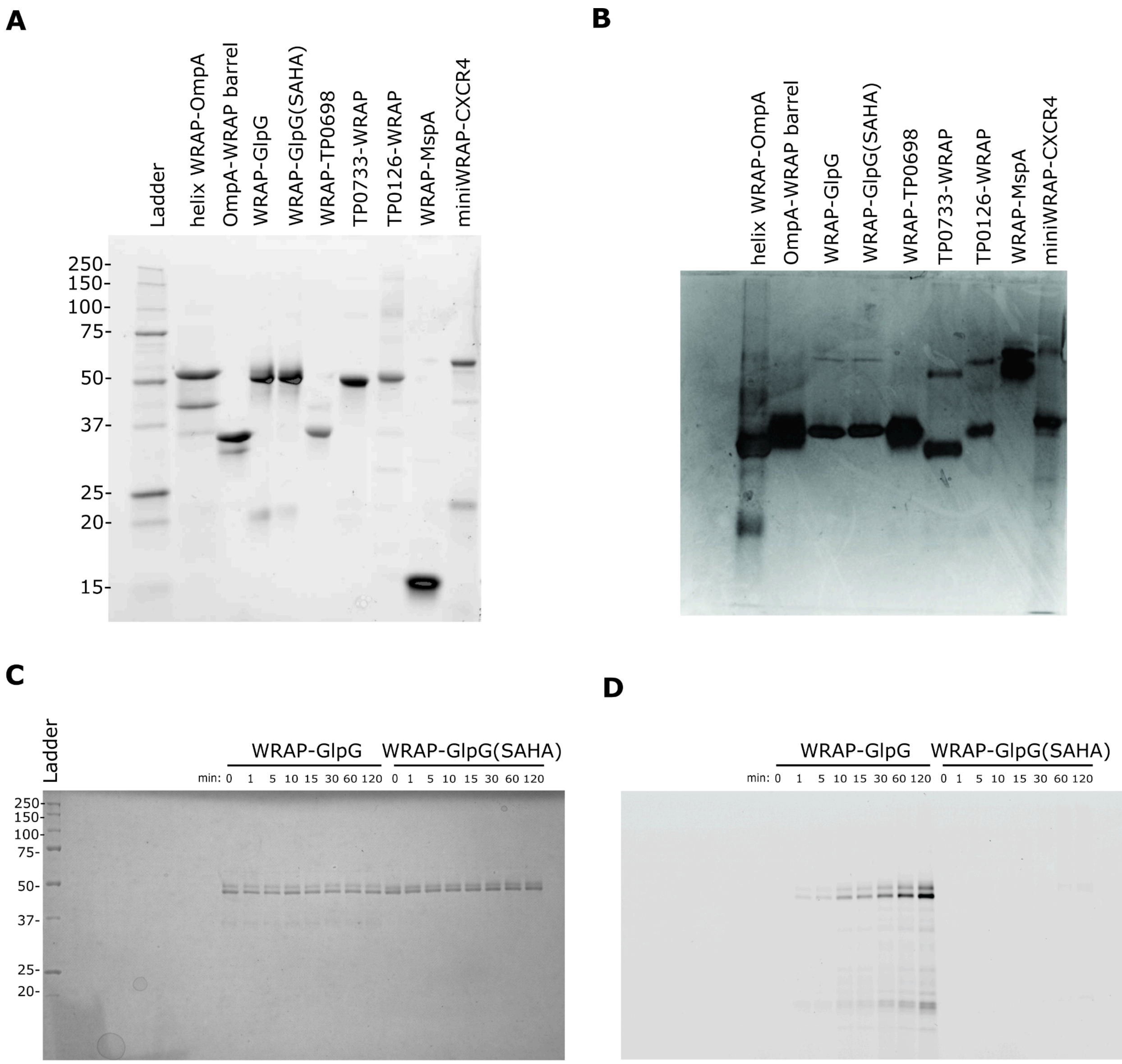

Fig. S15. Gel analysis of purified WRAP proteins. (A) SDS–PAGE gel of purified proteins (No boiling or reducing agent was used, and WRAP proteins migrated faster than their expected molecular weight, likely due to their compact structure and incomplete denaturation). **(B)** Native PAGE of purified proteins. **(C)** Uncropped SDS–PAGE gel of WRAP-GlpG WT and the catalytic mutant (SAHA) from Figure 4. **(D)** Uncropped SDS-PAGE gel of time-dependent FP-TAMRA active probe labeling of WT WRAP-GlpG and catalytic mutant from Figure 4.

Typical detergent-purified or refolded IMP yields from *E. coli* are: OmpA 0.5 mg (*44*) to 3–5 mg/L (*45*); GlpG 1–2.5 mg/L (*46*); MspA ~2.6 mg/L (*47*); hCXCR4 0.05–0.1 mg/L (*48*); and *Tp* OMPs ~0.5 mg/L (*49*). Using WRAPs, we obtained ~3.3 mg/L for OmpA, ~0.675 mg/L for GlpG, ~15 mg/L for MspA_SBDs, 0.1–0.5 mg/L for miniCXCR4, and ~1.5–3 mg/L for *Tp*OMPs. Yields were markedly higher for some proteins (MspA, *Tp* OMPs) and similar or slightly lower for others (OmpA, GlpG). Because some constructs are truncated (MspA_SBDs, miniCXCR4), they are not directly comparable to full-length detergent-purified proteins.

Table S1. Summary of WRAP design considerations, target difficulty, and experimental outcomes.

Target protein	PDB ID	Origin species	WRAP fusion	Truncations / mutations	Expression / challenge score*	Score cutoffs	Successfully solubilized designs/ Ordered WRAPs	Key design challenges / notes
OmpA (helical WRAP)	2GE4	*Escherichia Coli*	N-ter	None	Low	pAE_i<7 plDDT> 91	5 / 6	Robust expression; pilot-scale sufficient
OmpA (barrel WRAP)	2GE4	*Escherichia Coli*	C-ter	None	Low	pAE_i<4.2 plDDT>91	1 / 6	Barrel geometry has high computation demands to achieve similar scores to helical WRAPs
GlpG (helical WRAP)	2IC8	*Escherichia Coli*	N-ter	None	Moderate	pAE_i<7 plDDT>90	3 / 6	Low yields even when purified from detergent, but pilot screen successful
GlpG (S201A+ H254A)	NA	*Escherichia Coli*	N-ter	S201A + H254A	Moderate	NA	NA	Inactive mutant included as a control
Full-lengt hCXCR4 (helical WRAP)	4RWS	*Homo sapiens*	N and C-ter	None	High	pAE_i≤7 plDDT≥85 sap_ratio ≤ 0.588 binder_ca_rmsd ≤ 6.3	0/49	Poor expression in *E. coli*, with predominant truncation and accumulation in the insoluble fraction; no detectable soluble protein, likely due to long hydrophobic

								helices and intrinsic instability of full-length GPCRs.
mini CXCR4 (helical WRAP)	4RWS	*Homo sapiens*	N and C-ter	Δ46-91, Δ122-163,Δ207-251,Δ291-303	High	pAE_i≤5 plDDT≥90 sap_score ≤ 201 binder_ca_rmsd ≤ 2.0	2 / 46	Difficult expression in *E.coli* due to long hydrophobic helices, intrinsic instability and requirement for disulfide formation; expanded computational and experimental sampling
TP0126 (helical WRAP)	AF2	*Treponema pallidum*	C-ter	None	High	pAE_i≤6.3 plDDT≥90 sap_score≤300 binder_ca_rmsd ≤ 1.5	19 / 48	No structure available and challenging expression in *E.coli*
TP0733 (helical WRAP)	AF2	*Treponema pallidum*	C-ter	None	High	pAE_i<5 plDDT>85 binder_ca_rmsd < 5.0	3 / 10	No structure available and challenging expression in *E.coli*
TP0479 (helical WRAP)	AF2	*Treponema pallidum*	C-ter	None	High	pAE_i≤6.3 plDDT≥88 sap_score≤300 binder_ca_rmsd ≤ 1.5	4 / 24	No structure available and challenging expression in *E.coli*
TP0698 (helical WRAP)	AF2	*Treponema pallidum*	N-ter	None	High	pAE_i≤6.3 plDDT≥88 sap_score≤300 binder_ca_rmsd ≤1.5	7 / 24	No structure available and challenging expression in *E.coli*
MspA_SBDs (helical WRAP)	1UUN	*Mycobaterium smegmats*	N-ter	Δ1-70,Δ122-184	Moderate	pAE_i<8 plDDT>70	1 / 8	Protein is very large. All designs that pass the plDDT cutoff ordered
Heliorho-dopsin (helical WRAP)	6IS6	*Thermophilus archaeon*	N-ter	None	Low	pAE_i<5 plDDT> 92	3 / 6	Pilot screen successful

Aquaporin M (helical WRAP)	2EVU	*Methanobacter marburgensis*	N-ter	None	Low	pAE_i<4 plDDT> 92	3 / 6	Pilot screen successful

* *Expression/challenge score is a qualitative assessment based on prior reported expression yields, structure availability and pilot-scale outcomes.*

Table S2. WRAP sequences that expressed. Native membrane protein sequences are shown in italic and WRAP sequences are underscored, with characterized sequences in bold.

Design ID	Target Protein	AA Sequence	Average MW (Da)
PB0002	TP0126	(M)*LMAGRSTIRLSAMGAVPLFQVDWCNSGRGDDRNANAQTNGHKYIYPAFSAALGFEHFVCRGLSLGIDASVQYHCSYPNNTYSPTTPYYYLAIPVALTAGYTVAFWRIRLPLTVGAGFNYQHYYTSTYYGLVLKAAAGCYFQLTEHWSLGVSATYSGVPRSCEKIIEEDRQQTNTRTAQFIAAGVDVRYHL*(GGGSGGGSGS)RGWRLLALFAELMAALIAQPAPEELAARNAEINAEIREVLADEAALADAINAFLTLVVLAYQNAPLEELVAATRRLREAAAGLFADEPVARALAEAGLTLIALFLAGAPNEEILATLEEYVKRIRELAKGEEGVVTAMLTMMTLNVLAWVGAPQEEMDEVFEKMMAEMREVFKDDPLLLHLFSMFLVTAWHVFAGGDLERALAELQALRDKAIALAPEVAWAVTALYLFFALVAMRRAGRPRAEQEALAAELRAEARALLPEQLEHADTAAAMLDAMVALLLGEPVETLRARLTALYAAMAAEMEDPLERELVAAMARFVAAGLAGASREELLALLDEIIAILRRLA(GSGHHWGSTHHHHHH)	61378.2142
PB0024	TP0733	(M)*LMPIRPGDQLLKIGIVAGCQLYIAGGNGTNGSSSSGTNGNGNGKLLGGGGFHLGYEYFFTKNFSLGGQVSFECYRTTGSNYYFSVPITVNPTYTFAVGRWRIPLSLGVGLNIQSYLSKKAPGLIAEASAGLYYQYTPDWSIGGIVAYTQLGDIASSPDKCRAVGLATIDFGVRYHF*(GGGSGGGSGS)VAADFWGALLDAMILAYSGGPNAEIRAAIDRAIAAAEELFADDPHLKQVFVSMLEIMLYIAEGAPMEKITAAFERLADALAAIFAHDPALAALVRHFLRIVVLFITGGKNEELLEAVERLVEALEAAFAKIPEFGVLMQAVVKVLVMIYVGEPKEKIEKVFEEYIKKAYEVRTLPQAVFDMMVSMTKLVYLLLLNEPKEKVEKALDDLIDAVKAFYADDPVLGALMTAYLELFKLHALEAPQEEILETYERLVAEVRELVKDKPAEYQAFAAFMEMIMARFLGLDKAELTKLIKERTAAMKEVLEDKALADYFEQFFLALLHFENLEPVEEVRAHVRAAVEALRKLL(GSGHHWGSTHHHHHH)	60223.3154
**PB0027**	**TP0733**	**(M)*LMPIRPGDQLLKIGIVAGCQLYIAGGNGTNGSSSSGTNGNGNGKLLGGGGFHLGYEYFFTKNFSLGGQVSFECYRTTGSNYYFSVPITVNPTYTFAVGRWRIPLSLGVGLNIQSYLSKKAPGLIAEASAGLYYQYTPDWSIGGIVAYTQLGDIASSPDKCRAVGLATIDFGVRYHF*(GGGSGGGSG)KDVAEFLRHVLRAMALIAAGGDLAEIRREIEEAIEYVNRALADAVLSRFFLTSILEFWLDIVEGKSLEEIKARIDALYEEALAKDPERKDELEALKYIFLAAALFYTGGPVEEIVEYLEKAKEALEKIFADNEKAKKFATAIMDLAIKMVEKAPVEELLEAFTKAFDILIEMAKDDEELRALIEATRTLTEMMLKGADVATIEATIREQVARLKEVAKEHGALYLMMMLTIVEGVMTAAGADEATLRELTDELREAALEAAKVAPLAVRLFLTARDVTEAALRGASWAELDKRLDEA**	60011.4409

		**EKELQKETGEDDLIKFFVYFLRAGLLIILGGDVAEVEKDIDKAVEALEKIL(GSGHHWGSTHHHHHH)**	
PB0031	TP0733	(M)*LMPIRPGDQLLKIGIVAGCQLYIAGGNGTNGSSSSGTNGNGNGKLLGGGGFHLGYEYFFTKNFSLGGQVSFECYRTTGSNYYFSVPITVNPTYTFAVGRWRIPLSLGVGLNIQSYLSKKAPGLIAEASAGLYYQYTPDWSIGGIVAYTQLGDIASSPDKCRAVGLATIDFGVRYHF*(GGGSGGGSGG)KIVDLVLAIAELLLDIITGAPREVLERSRDRALELLREVAKDNVAGVTFLSFILELMVLLRTGAPVEEITALIDETIKRLETESPWDKKAIDVLTGALELLKLVIEGAPNEELRKQAEKLAKAVLDLTKDKPPEIQGMAIVAATAIIMVTETTLEEIEKLIEEAKKKIEEVLKDDEKLKNMYIKFLDLILEAMKGAPPEKIAELVLEATRLFAEYYSDDPAVRDFALALAELLALLLRGADRAELDAARERLAEAFARVTAPNGVTKELALLFLDFMVEMALGGEFERLRELIRQFYDVALATLPDKVGVAALALFGLLVAGIATRAPAEELRREFAEFAARLRAQA(GSGHHWGSTHHHHHH)	59606.4579
PB0036	TP0126	(M)*LMAGRSTIRLSAMGAVPLFQVDWCNSGRGDDRNANAQTNGHKYIYPAFSAALGFEHFVCRGLSLGIDASVQYHCSYPNNTYSPTTPYYYLAIPVALTAGYTVAFWRIRLPLTVGAGFNYQHYYTSTYYGLVLKAAAGCYFQLTEHWSLGVSATYSGVPRSCEKIIEEDRQQTNTRTAQFIAAGVDVRYHL*(GGGSGGGSG)MKTLRDFIFETLKDDKEVVERIEKLWREAVQALEEGKLDEAERLIALYFTLSLLFVMLKLLRERAADEPGLVERIEELERTVKEIAKEENIEEKAEELLLIALEAAFLLARIYAREVAADDKEALERIDRLERAVDEVRESGDVDRAILMLAVATFLLTALFFLIESRDIIENELGKLPVIKENLEETLRLVEEGNLEGALKQAMLTIALIVAVLSLVALRRRVAAEAGAEPLYDWLLARMDRALEEPDVERKTRELTLLAMLAGAVLMLHELAPLLREHSDLLPYLEELLAEAVRLLEAGDVEGAERKLTLLIVLLVALVLYVVVKELVGESSDLVEYLDQLLEKLKEALEKGDEDGSLRLATALLLLGITIFLYDLARRDIADDKEVLELIDRMVERLKELLDKGDLDGFIRQALLLNLFLT(GSGHHWGSTHHHHHH)	71697.2277
PB0040	TP0126	(M)*LMAGRSTIRLSAMGAVPLFQVDWCNSGRGDDRNANAQTNGHKYIYPAFSAALGFEHFVCRGLSLGIDASVQYHCSYPNNTYSPTTPYYYLAIPVALTAGYTVAFWRIRLPLTVGAGFNYQHYYTSTYYGLVLKAAAGCYFQLTEHWSLGVSATYSGVPRSCEKIIEEDRQQTNTRTAQFIAAGVDVRYHL*(GGGSGGGSG)MLEVRRRIDRILRERFGFLPVAEELEARARELLRQGDLEGALRTLLLRVTLRLGAAVMDLLLPRVLAAHGIEETVDRLLRQTREAFEAGDYEGGARLLVLLVALLWAADMLELVREWIKDDKELLETLERLFRRTLEALREGDLEGAVRLLALLVAMLLVALGLKLLKERYGEKSKVIEMLEKLLEELLELIEEGRVEEALRRAVTLAMLIISTIVIEALRELAHLLPDLVPLLERTTKRIEELLKEGKLEEATELALLTATLGMLRLLLVELRRLFGEEVRLLPQLQALLDQLAAAIEAGDLEGSRLLAATLTAALLAVLLLLVVRREAADDEEVQRLVRRLEDLLAEAFQSGDLEALLRVAATVLLIALALYWFLRYERSAGDQDPDIELVRALRDELARAVEQGDLEGALRLAQLLLAAVL(GSGHHWGSTHHHHHH)	71297.3073

PB0043	TP0126	(M)*LMAGRSTIRLSAMGAVPLFQVDWCNSGRGDDRNANAQTNGHKYIYPAFSAALGFEHFVCRGLSLGIDASVQYHCSYPNNTYSPTTPYYYLAIPVALTAGYTVAFWRIRLPLTVGAGFNYQHYYTSTYYGLVLKAAAGCYFQLTEHWSLGVSATYSGVPRSCEKIIEEDRQQTNTRTAQFIAAGVDVRYHL*(GGGSGGGSG)MQAEYVAFVRAALDETGARPLLDRLLAEALAALEAGNLDEAEYLLAQAAFLRLATLLLTLLKELLADDKETVELLERLFEEYLEALEKGDLEKSTILGGILLFMMALALIYHYAKTVFSEHKDLIEKLEEVLEKAREAVELEDLKKRLDLLTLYLLYAMLLLAAAALFHLTPEDERTHELAKRLVEEVEEAIEKGDLDKGLLLASEALIKLVTLLVLAALARLEAQETGREEEVRALRDEFLEAIEAGTWLETARRVLARAAVRLVRHLIRRLREAVPDLSPEVERLLRQAEELIERLEEGDEENLELLTAALVVLVVAAVLLWVVEREIADKLPRYPEIREAVRLLLDRAEDADLEERLRYYVAVLLLLMLAVFEHVFLPATRALFGAGPLVDLLLERLREALRRGDVLGATRLALLAILAVA(GSGHHWGSTHHHHHH)	71374.9126
PB0044	TP0126	(M)*LMAGRSTIRLSAMGAVPLFQVDWCNSGRGDDRNANAQTNGHKYIYPAFSAALGFEHFVCRGLSLGIDASVQYHCSYPNNTYSPTTPYYYLAIPVALTAGYTVAFWRIRLPLTVGAGFNYQHYYTSTYYGLVLKAAAGCYFQLTEHWSLGVSATYSGVPRSCEKIIEEDRQQTNTRTAQFIAAGVDVRYHL*(GGGSGGGSG)MQEEYVAFVREALTATGAEPLLRRLLAEAEAALEAGRLDEAERLLAQFVLLRLSTMVLHLLKELLADDKETVAALERLFQEYLKALEAGDERNATLLGGILLYFMAIALIHHVLRTEFAEDKDLIERAERVLEKALEALELDDLEETLDLLVLYLLIAQLILAAAFLRKLAPDDERLHELADRLVEEVEKAVEKGELEEGLRLAAEALQRLATLAVLHALERLEAQRTGRAAESRALLDEVLEAIERGTWRETLARALVEAAVRLIRHLIRELREAVPDLSPLQQQLLADAEAAIKRLEAGDEENAMLLMALAVVALVTAALLYVVEREIKDKEPRYEEVMAAVERLLARAQGADLETQLRYYVAALLLLMALVFRHVFADATVELFGAEPLLERLWRLLLEALERGDVEEATRLALLLSAAVA(GSGHHWGSTHHHHHH)	71052.1825
PB0047	TP0126	(M)*LMAGRSTIRLSAMGAVPLFQVDWCNSGRGDDRNANAQTNGHKYIYPAFSAALGFEHFVCRGLSLGIDASVQYHCSYPNNTYSPTTPYYYLAIPVALTAGYTVAFWRIRLPLTVGAGFNYQHYYTSTYYGLVLKAAAGCYFQLTEHWSLGVSATYSGVPRSCEKIIEEDRQQTNTRTAQFIAAGVDVRYHL*(GGGSGGGSG)MAELRERLRRTAREEFGAQPLLDELWARFREALARGDLDEATLLLALRFTLELLTAVIALLGPLIEETSGLFEKVRELFEKMLEALRKGDLKEALKHGMLTLLLIMLALAYRLAREEAKDDEELLRRLEEALELALERIEKGEVEKTLVYLTLLTFLFVNLYLLRRMARELRIDPDDYALLRARLRELLERLLEDPDLEGLYELLREIAKVTLAIVTLALAELLRRVRGREELADRLVAEVRRALEEDAPLEEWRRLLARALLEANRLMLEELLALDPSIAPLAELYEEVLKRIEELERKGDLEGYLKAVTLATLILITAAVYSAARRLAADDEELMADLDRLRDWFLRNFPSASIDEQIRLATMALLFFMALIFYHRFRDALADDEERVATIDRLLELTKELIDRGDIDGAIRTAMQLMLLIA(GSGHHWGSTHHHHHH)	72600.3895
PB0049	TP0126	(M)*LMAGRSTIRLSAMGAVPLFQVDWCNSGRGDDRNANAQTNGHKYIYPAFSAALGFEHFVCRGLSLGIDASVQYHCSYPNNTYSPTTPYYYLAIPVALTAGYTVAFWRIRLPLTVGAGFNYQHYYTSTYYGLVLKAAAGCYFQLTEHWSLGVSATYSGVPRSCEKIIEEDRQQTNTRTAQFIAAGVDVRYHL*(GGGSGGGSG)VRDYRAYVRAALEADPEAKEVVDKLLKKAKEYLEKGDIEGAELYIAKLFLVELALVLTRLLEERFGEEEELLEKIRELYRKM	71044.3791

		LKELKEGNEKEANKYGALLLALMALTIAYHYAREALQDDAEGLAALDEQRARALALLEAGDFEGFLLQAANAIFLAGLLFLAKRLAEEYADDEELVAALERLRALALEALAEPDLLALLRRLSALLMLLVALAVAAGLLRDVRQDEAGLREMLALVERTLDAMRAGDWEDALAEVMAAVMLLMNDFMLELLLELIADDEELKARLERNYAKLRAAIPTASLDEQMRLLTLQTVLLVAMAAVYVVRKRYAEESDLFKYLDEVLEKLEEAIEAGDYDGALRLATLLLLLAIAIYFYGLLQELRDEGSPLFEIVDKLLELAKEALEKGEIDEAIRLLLLASVAAA(GSGHHWGSTHHHHHH)	
PB0051	TP0126	(M)*LMAGRSTIRLSAMGAVPLFQVDWCNSGRGDDRNANAQTNGHKYIYPAFSAALGFEHFVCRGLSLGIDASVQYHCSYPNNTYSPTTPYYYLAIPVALTAGYTVAFWRIRLPLTVGAGFNYQHYYTSTYYGLVLKAAAGCYFQLTEHWSLGVSATYSGVPRSCEKIIEEDRQQTNTRTAQFIAAGVDVRYHL*(GGGSGGGSG)VREYRAYLQAVIDRDPALKEVVDALTAKALAALEAGDLDTLEFYIALRFLVELSAALLELLRERLGEEEPLLERAEELYRQMLEALKAGDLKEALKYGGLLTALMAVILARHYARRTLQDDEEGLAALDRLEEEALELLDRGDFEGFLLQAMILTFFAGLLFLLELLRRRYAEDERVVRLLEELRELALEALKEEDLLKRLRTLSALLILTTALAAAAGLLLEVEEDEEAFEEFLELVEETLEAIRAGDWEESLARVLNDVMLRMADFMLRELERLMAEDKELVERLRENAARLRAALPTADLLGQQRLLALLVVLLVAMAVYYVVRRRVGEESPDLELADRLLEELDRALEEGDYDEALRLATALLLLSLAIYFRALARELRDLGDPSFAILEALWERAREAFEAGDVDRLIRLLLLASLVAA(GSGHHWGSTHHHHHH)	71718.7893
PB0056	TP0126	(M)*LMAGRSTIRLSAMGAVPLFQVDWCNSGRGDDRNANAQTNGHKYIYPAFSAALGFEHFVCRGLSLGIDASVQYHCSYPNNTYSPTTPYYYLAIPVALTAGYTVAFWRIRLPLTVGAGFNYQHYYTSTYYGLVLKAAAGCYFQLTEHWSLGVSATYSGVPRSCEKIIEEDRQQTNTRTAQFIAAGVDVRYHL*(GGGSGGGSG)VAEFRDFVDAVLAKHPELKAYVDELLARAERALEEGDLETALLLLVQRVALELAAVYYEILRPLVAPHEELLARLEELWAELLAAVAAGDEERTLELLGKLLLLLAVAVAMVLADRLASDDKEGLAALDELTARFLELLDEGDLEGAARTAALLLLLAMITLALVFLRRLFGEESDSIELMEEIYREALEALRTLDDLEAIRRLLLLAMAALVILNIEMLGKLAADDPELQKQVEEIREEFKRLWEEGKLEELVARGLARVTLLMTHIILEELLRLFPEYAERLERLAELARELLEAPEDEDLEELMKRVVLLTVAAIALAVLIAIRREFKEVTPEDRLLEERLEELLELIERGEVEEGLRRGALLLLALIALFFYRLRERTVGERDPRLPLARELLEKLAELLREGRVEEALLYAQALLALVL(GSGHHWGSTHHHHHH)	71625.2871
PB0060	TP0126	(M)*LMAGRSTIRLSAMGAVPLFQVDWCNSGRGDDRNANAQTNGHKYIYPAFSAALGFEHFVCRGLSLGIDASVQYHCSYPNNTYSPTTPYYYLAIPVALTAGYTVAFWRIRLPLTVGAGFNYQHYYTSTYYGLVLKAAAGCYFQLTEHWSLGVSATYSGVPRSCEKIIEEDRQQTNTRTAQFIAAGVDVRYHL*(GGGSGGGSG)VDGLLARARRLTADHADAQVHVERLVARAAEAVAAGDEDTATFLTLLAVVFAQSVVFADRVLERAKDDADLVATLRALLEALRAALDAGDLAGAVRYLGLLTFLIAAAEFYLVVREVTPEDKERLARLDRLLAEALAALRAGDEDEAVELLTELVLLMIITLALHMIRQVLGDDEEARELVEMLVEKAEEALELPDRRERIRMLLLVAVLLIQVLALGMLRQVMADDEEALARIDETLERVKELLEEGRYEEAARYALVVTMLLAVRFMLRELEPLLKEERPLLEKVRELLEETEELLEEGELEEALLV	70813.0311

		AVTAVVLLITAALLTVLRREAKDDKELQEMVERIRELVLEWLEEEDVEELLRHLALAAQLLLALLLLLQYRRVEADAGGAAEELLAAIREAVELLEAGDLAGARRALQRVLALLA(GSGHHWGSTHHHHHH)	
PB0063	TP0126	(M)*LMAGRSTIRLSAMGAVPLFQVDWCNSGRGDDRNANAQTNGHKYIYPAFSAALGFEHFVCRGLSLGIDASVQYHCSYPNNTYSPTTPYYYLAIPVALTAGYTVAFWRIRLPLTVGAGFNYQHYYTSTYYGLVLKAAAGCYFQLTEHWSLGVSATYSGVPRSCEKIIEEDRQQTNTRTAQFIAAGVDVRYHL*(GGGSGGGSG)ESLLRVRAEEELADDEELRRMIEYLLEKAEEARAAGQEDEAREYLVAAITAYLAGLVLRRLLALEADDEELREEVEELLEEAEEYMEKGDVRGMLAVLMEAVVLAVTRVLERLAREVLADDEVFREILERLVERAEEAKKEPDLFKRLDDLSRIYMMIGMVLMMHLIAERLVERLGLAELVDRIRRELLEAAEGDDLDALLRRIAVHTALLLGLLAAHELLERIEGDEEIAEIAREIAARLVELLAEPDAEEHSTELVLLILIIALIMILLLVRELLPEDKELHEKLKEKIKEILKLVEEGKLEEAILAAIRYIALGLAYIVAEVLARLAEEDEELVERAEELREMILELLDSEDYEESLVKATAILLLNIAALFLLRQEELGYSSLPAYARHRAELREYYEGLKAGKWEELLPGLLLLAVELA(GSGHHWGSTHHHHHH)	72003.2268
PB0066	TP0126	(M)*LMAGRSTIRLSAMGAVPLFQVDWCNSGRGDDRNANAQTNGHKYIYPAFSAALGFEHFVCRGLSLGIDASVQYHCSYPNNTYSPTTPYYYLAIPVALTAGYTVAFWRIRLPLTVGAGFNYQHYYTSTYYGLVLKAAAGCYFQLTEHWSLGVSATYSGVPRSCEKIIEEDRQQTNTRTAQFIAAGVDVRYHL*(GGGSGGGSG)QSLLKVRAEKYLKDDKDMQEMINKLLEKAEEARKKGQEDEYREYLVAAITALLAGEVIRLLLALLADDEELREEVEELLEQAKEYMEKGDVKGALLVLMLAVLLLADAVLERLAREVLADNATAMAILDILVEKRREALKEPDLEKRFENTALLYIMIGQLLMFHLIAERLAEELGLGELLDRQLAALLEVMEGDDLVAALRAMAHRTSLALNLLAAYEIKKRLKGDEELREIAEKIAEELVALLREPDIEAHSDRLVLLTLMIALMMIYLITKELLPEDTELHEKLKKKIKEIWELVEEGKLVEAIKAATRYIALGMAYLVAHVVAELAADDPDLVERARELRDRILELLNSEDYDKSLIEATAILLLLIAEMFLLVQERLGYSELPEYAEFRAELRELYEGILEGRVEELLPRLLLLAVRLA(GSGHHWGSTHHHHHH)	71894.7257
PB0068	TP0126	(M)*LMAGRSTIRLSAMGAVPLFQVDWCNSGRGDDRNANAQTNGHKYIYPAFSAALGFEHFVCRGLSLGIDASVQYHCSYPNNTYSPTTPYYYLAIPVALTAGYTVAFWRIRLPLTVGAGFNYQHYYTSTYYGLVLKAAAGCYFQLTEHWSLGVSATYSGVPRSCEKIIEEDRQQTNTRTAQFIAAGVDVRYHL*(GGGSGGGS)GFDVLADVLIAQAEATSGIEPLVRKLLEEAKEALKKGDLEEAIFLLTLVVTLVYASQETARLLAEATEDTELLAALRRTLEEMVKALEEGKLEEALKLGALLLALQMLLRMLLYARRLHADDPEMQAVLDEVRARVLEALKSGDLEHQILTLMTATFRVGAEIMRRELAEVLEEDPEAKEAIEKLWAKADEAIEAGKVEESLLLLSQLIGLMVLTVALRVLRELLERHGFDPSVIDRLYEELLEATKSGDLEKFRRTAAYVTAAAAALMALLLGIELFRDDEELVAELRERIAELEEVIRAGEKERGFRLALLITLYVIARMAARVAREVLKDNKTVHRLVEALLRRLEEAIRRGDVEGAMRLAALLLMTIVAVVLAKKRTEEFPELAPLMEELLRLVEQLLEAVEAGDVEEAELLARLLLALTV(GSGHHWGSTHHHHHH)	70984.6317

PB0070	TP0126	(M)*LMAGRSTIRLSAMGAVPLFQVDWCNSGRGDDRNANAQTNGHKYIYPAFSAALGFEHFVCRGLSLGIDASVQYHCSYPNNTYSPTTPYYYLAIPVALTAGYTVAFWRIRLPLTVGAGFNYQHYYTSTYYGLVLKAAAGCYFQLTEHWSLGVSATYSGVPRSCEKIIEEDRQQTNTRTAQFIAAGVDVRYHL*(GGGSGGGSG)FDTLAEVLIARARAVSGAEPLIERILQEAKAALDAGDLETAIFLLTLAVTLVRALERVAELKARATEYKELLAFLEETAEKMVEALKQGDLQTALKLGLLLLALMALLEMLLYARELLADDERARERLEEVLRVVLEAQKSGDLEQQIVTLMAATFRVAAEIARRLFADALAEDPEVAAALEKLEAQADEAKARGDLEGTLLLLSALLGLMVLTLALRELRRLLEEAGEDPAVVDRLLARLLEATESGNLEEFLRTMAWVTAAVAALMALLLAIELFADDEELVAELRERLERLVEAIRAGREEEGFRLALLITLYVIARMVARVARELLADDELAHELIDLLLERLDAALAAGDFDGAMRLAALLIETAVALVLAHARTRVHPELAPLMEVLRALLAELARALEAGDFEGAALLARLLLAVTA(GSGHHWGSTHHHHHH)	69872.2237
PB0072	TP0126	(M)*LMAGRSTIRLSAMGAVPLFQVDWCNSGRGDDRNANAQTNGHKYIYPAFSAALGFEHFVCRGLSLGIDASVQYHCSYPNNTYSPTTPYYYLAIPVALTAGYTVAFWRIRLPLTVGAGFNYQHYYTSTYYGLVLKAAAGCYFQLTEHWSLGVSATYSGVPRSCEKIIEEDRQQTNTRTAQFIAAGVDVRYHL*(GGGSGGGSG)LELRARVLVERAAKTSGAEPLVERLLQQARQALEEGNLEEAIYLLTLVVTLVDALEEVERLLERATEYTELLQAARETRDQMLAALEAGKEQEALRLGALLLALLALIRMLLYAKELLSDDPEAQEVLARVQARALEALKSGDIDEQILTLMALTFEIALEIALREFDAELEEDPEAKKAIEELRAQADALRAAGDIEGYLLALSQALGLIVITLALRKLKELLEEAGFDPAVIDELKKRLLEALKSGDLVERLREMTYVTLAVAALLAILLAIELSRDDEALVAELRQQYEELVEVIRAGKKEEGFELALLITLYVLARMAVLVARRVLADDEVVHRFLDLLMARADELIAAGDVDGAKRLLAILLETVVAVVLARRRTEVHPELAPLMDELLAIVEEALERLEAGDLDGLRLLVRFLLALTA(GSGHHWGSTHHHHHH)	70542.8574
**PB0073**	**TP0126**	**(M)*LMAGRSTIRLSAMGAVPLFQVDWCNSGRGDDRNANAQTNGHKYIYPAFSAALGFEHFVCRGLSLGIDASVQYHCSYPNNTYSPTTPYYYLAIPVALTAGYTVAFWRIRLPLTVGAGFNYQHYYTSTYYGLVLKAAAGCYFQLTEHWSLGVSATYSGVPRSCEKIIEEDRQQTNTRTAQFIAAGVDVRYHL*(GGGSGGGSG)MALLRERLVRRARETSGIEPLVEKLLDEAERALAEGDLERAIFLLTLAVTLVRSSEEIARLKEEATDLTELNAAAEELFQQMVQALEEGRLQEALELGGLLLALLMFIRMLLVAKERHSDDEEMKEVLNRVIERALKALESGDRTYQLLTLMLNTFIVAAKMMEREFRAELEADPEVRAALERLKKELEELLKEGKVEDALRAASRLIGLMVLSLALRALRALLREQGRDPGVIDLLRERLLAATESGDEDELDRTMTYVTAMVAALMALLLGMELTADDEALVARLRERYDELVAAIRAGDEEGGFELALLITMDTIARMAALVAREVLRDDEVVHRLVDALMARAEELIARGDLEGALRTLALLLRLVVAMVLARALAEVYPELAPLLEELRALAQRILAAVEAGDLEGAETLARLALAVTA(GSGHHWGSTHHHHHH)**	70627.9595

**PB0073_rigid linker**	**TP0126**	**(M)ALLRERLVRRARETSGIEPLVEKLLDEAERALAEGDLERAIFLLTLAVTLVRSSEEIARLKEEATDLTELNAAAEELFQQMVQALEEGRLQEALELGGLLLALLMFIRMLLVAKERHSDDEEMKEVLNRVIERALKALESGDRTYQLLTLMLNTFIVAAKMMEREFRAELEADPEVRAALERLKKELEELLKEGKVEDALRAASRLIGLMVLSLALRALRALLREQGRDPGVIDLLRERLLAATESGDEDELDRTMTYVTAMVAALMALLLGMELTADDEALVARLRERYDELVAAIRAGDEEGGFELALLITMDTIARMAALVAREVLRDDEVVHRLVDALMARAEELIARGDLEGALRTLALLLRLVVAMVLARALAEVYPELAPLLEELRALAQRILAAVEAGDLEGAETLARLALAVTAEAAAKEAAAKEAAAKEAAAKAGG*LAGRSTIRLSAMGAVPLFQVDWCNSGRGDDRNANAQTNGHKYIYPAFSAALGFEHFVCRGLSLGIDASVQYHCSYPNNTYSPTTPYYYLAIPVALTAGYTVAFWRIRLPLTVGAGFNYQHYYTSTYYGLVLKAAAGCYFQLTEHWSLGVSATYSGVPRSCEKIIEEDRQQTNTRTAQFIAAGVDVRYHLG*(GSHHWGSTLEHHHHHH)**	**72101.87**
PB0076	TP0126	(M)*LMAGRSTIRLSAMGAVPLFQVDWCNSGRGDDRNANAQTNGHKYIYPAFSAALGFEHFVCRGLSLGIDASVQYHCSYPNNTYSPTTPYYYLAIPVALTAGYTVAFWRIRLPLTVGAGFNYQHYYTSTYYGLVLKAAAGCYFQLTEHWSLGVSATYSGVPRSCEKIIEEDRQQTNTRTAQFIAAGVDVRYHL*(GGGSGGGSG)MEILKEILVKRAKETSGIDPLIEKILDKAEQALEEGDLETAIYLLTLAVALVKASEEVKRILEEATWLKELNAFLEETLQKMVEALEKGDLKEALKYGLLLLALIAFLRMLIYARQRHSDDPEMQKVLDEVIKDALEALKSGDLDYLILTLMRNTFEIGAEMLRREAAAALDADPRVKEVIDRLWEKAEEAKKKGDVEESLRLLARLIGTMVLSLALQVLRELLERHGFDPSVVDRLYEEFLAAMESGDLEKQDREMTYVTLMVAALMAILLAIELFRDDEELVKRLREMYERLVEAIRKGEKEEGFDLALLITMYVLALMARRVAREVLADNEEVHELLDLLMERLEEAIEEGDLEGALELAAILLMTVVAMVLAHARTQQYPELAPLMELLLEIVREIKERLEAGDLEGARLLVEFLLAVTA(GSGHHWGSTHHHHHH)	71794.5231
PB0078	TP0126	(M)*LMAGRSTIRLSAMGAVPLFQVDWCNSGRGDDRNANAQTNGHKYIYPAFSAALGFEHFVCRGLSLGIDASVQYHCSYPNNTYSPTTPYYYLAIPVALTAGYTVAFWRIRLPLTVGAGFNYQHYYTSTYYGLVLKAAAGCYFQLTEHWSLGVSATYSGVPRSCEKIIEEDRQQTNTRTAQFIAAGVDVRYHL*(GGGSGGGSG)FALLRELAARDLADDETALALAERLLAEAEAAAAAGDEDAYREHLLQLAVVRLAAQVLRLLRERILEEIDGEELIDQAYEALEAILAGEDVTLNLAKLVLNVLLLVLELVIHEFAPQLEALGGALEALQELLEKAKETLKSKEKEKGLEEALLILANAALLLMTALALQALVEVSGDEDLAAKVLERAFEILKEKDLRKMTRDLLHLAVATILLLALELLIKHYADTSDRVEEMRELREKIKKAIEEGEIEKVLAEALIKLILIMVEFMLDEMRPFLRDGSRLVEHVEELLEQTEKLLKEGKIEEAMKYAVIATVLLVVLAAHLALLEVYADDEATRALLRELEAVLEARLELPDVEALLLAATTALLALVVLAWAGELRRRYSDLPALAPWVDSLRARLREILRLPDLREALARVQELLLEVL(GSGHHWGSTHHHHHH)	70879.5371

PB0079	TP0126	(M)*LMAGRSTIRLSAMGAVPLFQVDWCNSGRGDDRNANAQTNGHKYIYPAFSAALGFEHFVCRGLSLGIDASVQYHCSYPNNTYSPTTPYYYLAIPVALTAGYTVAFWRIRLPLTVGAGFNYQHYYTSTYYGLVLKAAAGCYFQLTEHWSLGVSATYSGVPRSCEKIIEEDRQQTNTRTAQFIAAGVDVRYHL*(GGGSGGGSG)LELKRELARRELADDELFLAAVEKLLDKAEEALEAGDKDTAREYLLQYAVLLLAGQVLRLLRERILEEIDGEELVERAYEAVRRILEGGNVRLNLARLVLTVLLLVLRLIIHDFKEELEKLGGVLELVEELLEEAEETLKSPEEEEGLERATLILAIAQQVIMIALSLYEIEKVSGEKELVELVKKKLFEILKLEDLRKALRELTYLAIATILLLAAVLLIKHYEDTRSDVEEMKELKEEIKEAIKKGEIKKVLPEALVRLMRIMIRVMLDELKPLLEDGSPLVEHVEKLLEETEKLLEEGKLEEAMKYSVIATVTLVVLALLIALRRLFADDEELVELLREIEEVLVERMKEEDVDYLLKAVTSALLALVILAWAAVQERVYADIPGLGPWRREVLERIREILEEEDEDKRLERLQELLLEVL(GSGHHWGSTHHHHHH)	72403.3408
PB0080	TP0479	(M)SGEALEWFEKQLKEMVEYLTKKYPIIDFQVRLLFWYLLLALRIHEGASREEIKEALDEVIEKVKELNPAAVRLAELIAETLLLLLDALEARDLEAIKERFFAILDRIAEIYGTSEAMVADMKERLDLALKLMESGTVEAALEAMRAQLDKLVAQVPQAAPLADAMLAVAETLAARLDEDPVAVVRAVVDAWAAALRAAYPEHAPLVEFVRALVDILLLIARGASLEELLAALEEFEKKARELLPDLKVAIDLTYLLLRTFLELLFR(GGGGSGGGGSG)*VFAAEVFVSPKVGQVGAHPWGKEPAPRTDVLLYTPTLGLAVGVIAHNGFAFTTELDAGLAFLMFRAQFLVGWAIRSGKNFAFIPSLGVDILSTQDDKLVGIPINLDFLIFFTKHVGLDFTFGSGVNVPLTKNLKKGANGNGQDLTWSNIWAKYCCTSLVMVKIGPVFRI*(GSGSHHWGSTHHHHHH)	50810.8794
PB0092	TP0479	(M)SGRALERLRELGERVVELAEEILPVLAHLFRWAVELLLGFLRAHEAGSIELLGEMLRELAAKAAPTDGAVAGVLELNAIVVDGIIEMEKKGIKENFLEGIRKILDRLTAIIPELAPMMERTLEIAERMLKAMETGDVDVVVETMQEIVREMLRRAGRPEEAAELAEAAVEAAAAMLKGDPAAEMERLAEHWARLLDLLLPEFPETAEIARLMLELTFRMATEDPLATAEEYLTRIREAYARMDPRLAGVVEVARALVLGFLEITLE(GGGGSGGGGSG)*VFAAEVFVSPKVGQVGAHPWGKEPAPRTDVLLYTPTLGLAVGVIAHNGFAFTTELDAGLAFLMFRAQFLVGWAIRSGKNFAFIPSLGVDILSTQDDKLVGIPINLDFLIFFTKHVGLDFTFGSGVNVPLTKNLKKGANGNGQDLTWSNIWAKYCCTSLVMVKIGPVFRI*(GSGSHHWGSTHHHHHH)	50474.3711
PB0094	TP0479	(M)SGAELAALEELMERVVEAAEKISPALAHLFRHAGRLLLAFLRAHRAGSVAALADDLAAAAAEAAPTDGALAGVLRMQRIVVERVVEMLEKGAEKTFLEQVRRTLEELTALIPETKPMMDRILEIAERMLEAARTGDVDVLLATMQEIVREMLDEVGAPEELAALAEALLAHVAAILEGDPAAEMARVAEAWARLLAALLPQFPQTAEIARLQIALTFDLATKDPLATAREHLGRIREAYARLDPELAGVVEVTELLVTGFLEVLLL(GGGGSGGGGSG)*VFAAEVFVSPKVGQVGAHPWGKEPAPRTDVLLYTPTLGLAVGVIAHNGFAFTTELDAGLAFLMFRAQFLVGWAIRSGKNFAFIPSLGVDILSTQDDKLVGIPINLDFLIFFTKHVGLDFTFGSGVNVPLTKNLKKGANGNGQDLTWSNIWAKYCCTSLVMVKIGPVFRI*(GSGSHHWGSTHHHHHH)	49644.2546

PB0103	TP0479	(M)SGSLLELAEQLLAEMVEEVTEKYPLFRLLAEILALGFLAILLLTDPSKKAEVEETVQKLIDLLEKQLKGAEGLVALVRIQFETALRVIEGPPLPGLLEEAERTLERIAELFPESAEVMARMLRVMREAVEIGKDATDEAVREMMRLIVEEVTADSPRRDEALEDMLRLVDLLLENRDADPVEFVRRAVELFLEIMRRLVPGMEELLEIGELLVEVTILLAAGAPAEEVLAAVRRLAETMIAFDENLAGAAFMLERLVRAFLELLEA(GGGGSGGGGSG)*VFAAEVFVSPKVGQVGAHPWGKEPAPRTDVLLYTPTLGLAVGVIAHNGFAFTTELDAGLAFLMFRAQFLVGWAIRSGKNFAFIPSLGVDILSTQDDKLVGIPINLDFLIFFTKHVGLDFTFGSGVNVPLTKNLKKGANGNGQDLTWSNIWAKYCCTSLVMVKIGPVFRI*(GSGSHHWGSTHHHHHH)	50703.7071
PB0104	TP0698	(M)SGALVDRVRAMFERVAAKLEELAPGREGTVEALRLMGEQAARAMEGDALAGLFEAWRALMDYATDQIPGSRPVMEALLDLVQTLVETLRAGDVEGARAAVDEFAARLVELFPGAERAARVLRALFDLMLDLFAGGFFSELEARVQALFDLMAELEPERRAEIERLAADLLAALRAVLAGDIDGLVDHIRAMVERLVAEYPEGAVMTPFAELVTDLIRLAAAGAPLEELAAVVRRRLDELAAAVTTGRHVVELTRTLLLEMLELGLA(GGGGSGGGGSG)*ALELFLSPKIGITSVYQFGSNGGSDGTSSGKGVSFDRLIGRVDLGLILVNGLTISASAESSLTNVFVRAQALIGYAVRVGGLRAIVSSGVNICGDSCATSEGKSSAWYSKLLYSVPLNLEVQYYLTSFAGVAVAASTAVGVRDFNFKEFTLPLSLTIGPTFRV*(GSGSHHWGSTHHHHHH)	48462.2815
**PB0110**	**TP0698**	**(M)SGAIYETIRNLLNELFDRLAEIRPERKEEVEIQRVIMLKILDIVLAETEEERKKLIEELLKELAKRSKAAAVLAELVVTFMLRLVEALRRRDRAAFEAAVEEFAEELVKLLKDAEPAAEALRRMLRALAALYFDRDVDAFVAHLRELADVLAKHSEEAEEIRYYIDLLIETLRRMLAAGDADAMVDILRETALKIVDEHEEAKFGRELVERMMAAFRAAVEARTLAAVEAFTDTMVDAFRDIMNVPESSAEAIRAMFDTLLALARA(GGGGSGGGGSG)*ALELFLSPKIGITSVYQFGSNGGSDGTSSGKGVSFDRLIGRVDLGLILVNGLTISASAESSLTNVFVRAQALIGYAVRVGGLRAIVSSGVNICGDSCATSEGKSSAWYSKLLYSVPLNLEVQYYLTSFAGVAVAASTAVGVRDFNFKEFTLPLSLTIGPTFRV*(GSGSHHWGSTHHHHHH)**	49907.0004
PB0113	TP0698	(M)SGAIVETIRATLDRLFDRLAEIRPERREQVEIDRVLMLYVLDIVLAETDEEQERLLEELIEELKKKSEAAALAARLVVSLMLALRRALRAGDEAAFRAAVEAWAAELVELLGDAEPAARALEAMLRALAALYFRGDVDAFVAEMRRYADILAEHSEEAEEIRRSIDLLLDTLRRMLAAGDADEMLAILEETVMTIIDEEEEAVFGRALAEETMAAYRAAVAARTLDAVAAFADTLVEAFTRYYGVPAESAEAIRALFEALLELARA(GGGGSGGGGSG)*ALELFLSPKIGITSVYQFGSNGGSDGTSSGKGVSFDRLIGRVDLGLILVNGLTISASAESSLTNVFVRAQALIGYAVRVGGLRAIVSSGVNICGDSCATSEGKSSAWYSKLLYSVPLNLEVQYYLTSFAGVAVAASTAVGVRDFNFKEFTLPLSLTIGPTFRV*(GSGSHHWGSTHHHHHH)	49131.6477
PB0119	TP0698	(M)SGARAVLAEWARTVRKLAEALEALGYELEARNVRVFYDALQAVLEDPSKAIDVAVKRLEEIVEELKKEGREAEAYLAEKLLRLVKAVSLPPKEFMARTAEVLRELSELFRSKGFTRGAELLDRFAEIVEAGAEGVEEMVRAMAEFARWLAEEHLKEGNEAAAEALRRFAAVLEAIFEGADALLAATRATIAALLAEARKAGSEIMAPVLDLMAEALELAFAGDPAFLEAVARMLETLAEIARSLGAERAYLALEMMRDMVRALLA(GGGGSGGGGSG)*ALELFLSPKIGITSVYQFGSNGGSDGTSSGKGVSFDR*	48721.5555

		LIGRVDLGLILVNGLTISASAESSLTNVFVRAQALIGYAVRVGGLRAIVSSGVNICGDSCATSEGKSSAWYSKLLYSVPLNLEVQYYLTSFAGVAVAASTAVGVRDFNFKEFTLPLSLTIGPTFRV(GSGSHHWGSTHHHHHH)	
PB0120	TP0698	(M)SGARAVLAAWAAALRELEEAFRALGEELRARNVEVFAAALEAVLRDPSRAIPVAVEKLEEIAKWLREQGKEAETYLMEKQLELVAAVSLPPEEFYRRVAEVLEEMAELFKEKGFERGAWLLERLAEIVRAGAEGVEAMVRALAEFARELAEEHRELGDEAGAEALDRFAEVLAALFEGDEALLAKTREVIDALLAEARKAGSEEMAPVLDLMAEALELAFAGDPAFLDAVRRMLEALAALAERRGAELGRLALRTMARQVAALAAA(GGGGSGGGGSG)*ALELFLSPKIGITSVYQFGSNGGSDGTSSGKGVSFDRLIGRVDLGLILVNGLTISASAESSLTNVFVRAQALIGYAVRVGGLRAIVSSGVNICGDSCATSEGKSSAWYSKLLYSVPLNLEVQYYLTSFAGVAVAASTAVGVRDFNFKEFTLPLSLTIGPTFRV*(GSGSHHWGSTHHHHHH)	48682.2248
PB0124	TP0698	(M)SGMEEILQTFADLLEELAELHRKKGDEFTAKQLLYFAEIVKALLEGEEAALATAVRLYEELVEELRKKGFIPEALMMEAAREMFRAAGEGPEAIFAAVAASLDKLRDYFKSLGFSEAAKLVDRLAEIVRALAEGIEAAVQRFIELARWLLDQAKEKGDEKTAEDLERLLQILEALLEDPEKALELFREAIERTLKDVEEAGDEIGREFAKAVAEAAELFGGPVEEILAVVAERLEKAAELLKERGDEMGAAAVESMARLVRTMLAS(GGGGSGGGGSG)*ALELFLSPKIGITSVYQFGSNGGSDGTSSGKGVSFDRLIGRVDLGLILVNGLTISASAESSLTNVFVRAQALIGYAVRVGGLRAIVSSGVNICGDSCATSEGKSSAWYSKLLYSVPLNLEVQYYLTSFAGVAVAASTAVGVRDFNFKEFTLPLSLTIGPTFRV*(GSGSHHWGSTHHHHHH)	49088.6679
OmpA_F2	OmpA	(M)SGSARAELFLHLLRLLRDGVAGAPREVIAAEREKAREALVALASGDPQGVKLVQAWFDLLDTMGASTPMETILKKVQAVVDALAELIPDNPAVVDFVYYTMSLMAQALSGGPLEEMKELAEKALAALEELLADDPVAQLLVVLTYRIILAAVMGAPLEELEKLYHELVEELDKVEDQFDADQLLFAAFVLIGLGRAVGEPLEKLEELYERYMSRVRELLADDRATVLFNEIFLLSMLYAGDEEKAEELAKRLDELESALREELADDPVRRLSAEIVTTLIRYLTLPVPPETAVRRLTELRERLEEAMEDPIGAVLLSAFVEMAIAYVGGLPPEELAARLAELVERLEAAADYQ(GGGGSGGGGSGGGGSGGGGS)*APKDNTWYTGAKLGFSQYHDTGFINNNGPTHENQLGAGAFGGYQVNPYVGFEMGYDFLGRMPYKGSVENGAYKAQGVQLTAKLGYPITDDLDIYTRLGGMVFRADTKSNVYGKNHDTGVSPVFAGGVEYAITPEIATRLEYQFTNNIGDAHTIGTRPDNGMLSLGVSYRFGQGEAA*(GSGSHHWGSTHHHHHH)	61261.9935
OmpA_F3	OmpA	(M)SGDNVKKLLQALRDLAVAFLRGEPVEKIAEYRAKAKEYLLALLADNPEGAAVVEAWDHLLDLIGAGAPLEELRAALERLRAALKALLKDQPDEAEVIYYMVEVYVLALTGAPRAELEAARDKALAAMEKMVKGDADTKAAVVELARAILLTMIGAPIEEIVEALKKAVEYLKKVTRKTKDYSYGLTLQLLTTYHLVAGKEVEEALKALDELVEELKALLPEKARLIELVRLQQEILLLSVYGAEAEELEKLLERVRRLLRELVERGDRAGTLLAVGVLTLAAAALQLPEEEVEELLAELERILREVVPDELAVEALTNFFRALLYAVQGKPAEEVRDVVERYFDAVIEKDYQ(GGGGSGGGGSGGGGSGGGGS)*APKDNTWYTGAKLGFSQYHDTGFINNNGPTHENQLGAGAFGGYQVNPYVGFEMGYDFLGRMPYKGSVENGAYKAQGVQLTAKLGYPITDDLDIYTRLGGMVFRADTKSNVYGKNHDTGVSPVFAGGVEYAITPEIATRLEYQFTNNIGDAHTIGTRPDNGMLSLGVSYRFGQGEAA*(GSGS	61377.4762

		HHWGSTHHHHHH)	
**OmpA_F4**	**OmpA**	**(M)SGELEQKAVETIAEMAIQMMLGGPSEEIRELWDKFIALIEEITAATPVETALARAMAELVRNLALGKPIDEIEKSAREFAAKLKELYGDRTAEAEAILAIVAAVKAGIEGKPKEEIAELLARYRDAVAKILADDPARQAVMTAVLDLIAALLLGEPAEEVRARLAELEALIDRLFDQLTPEELMFAAYTLLLAALAIGLPAEELLALLERLVARLREALADDPEALHIAELHLRVLVLTQLGKPIEEILELIKEILEEYKEELGEPTASFLVHALSLLLAVAISSRDTWELAKPLIDELLKGALEKLEDENVANYIKAFADLMKAAIEGKPIEELAELFRKMTEYLIKIIDYQ(GGGGSGGGGSGGGGSGGGGS)*APKDNTWYTGAKLGFSQYHDTGFINNNGPTHENQLGAGAFGGYQVNPYVGFEMGYDFLGRMPYKGSVENGAYKAQGVQLTAKLGYPITDDLDIYTRLGGMVFRADTKSNVYGKNHDTGVSPVFAGGVEYAITPEIATRLEYQFTNNIGDAHTIGTRPDNGMLSLGVSYRFGQGEAA*(GSGSHHWGSTHHHHHH)**	61206.432
OmpA_F5	OmpA	(M)SGMDKVELFVLIMKYMAYSALGADQEELDKIRKEIEEKLEKIIKEDPKNGTKAKIVVTMILALAEGVPVEELRKELDKLLEEYEKLDPENKAELAVFREAGEVVLAFREGAPDERLRELVLRLFRTIVEEAIKDPVLKEVMLAIVELYELMMRGAPLKEVEKKLEEVVDLLEENVDKLSPLQLAALVMFTVMLLLGMTLPEEVRERLLKRLEEILKRGLADDPALGRFVLTWMELLRLSQEGAPLEELEEVLERLLEALKELLPDRRDVAEAYYHFLRLGVAVAAGRPVAELAALIDAAEAAVRRAVEDENKAAFFAALLAALRLWIEGAPKEEIKAEFEKAAEALLAYYDYQ(GGGGSGGGGSGGGGSGGGGS)*APKDNTWYTGAKLGFSQYHDTGFINNNGPTHENQLGAGAFGGYQVNPYVGFEMGYDFLGRMPYKGSVENGAYKAQGVQLTAKLGYPITDDLDIYTRLGGMVFRADTKSNVYGKNHDTGVSPVFAGGVEYAITPEIATRLEYQFTNNIGDAHTIGTRPDNGMLSLGVSYRFGQGEAA*(GSGSHHWGSTHHHHHH)	62302.8412
OmpA_F6	OmpA	(M)SGEKRLEILELMLEIFLLGAIGADPEEIRKARERLKELVLELAKDNEKVRIATRLALRALEVAAQPIPLEELEKEFEKLKKEYYEEIEEHDHAFDLFLYLLMALRAARLGGDKKLIDEYYEKAKKSLKEILKDDPKAKELALLFLEALYLIWKKAPIEEIVEALRKATEVLEEMQAEYPDSALMAAAMLRLVIAMLSEKDLAALEEEFERVIREVRERLPEDALLLEFFFRLLLLSAKIEAGASPEEVAKLKEELLALYERIAAEGSWAQLAMASFLLMGLAIAEGEPLERLRALLDELLSRLRARAPDESAVEILEAILELAILAAAGEPNEVIRAAFERHLARLREIADYQ(GGGGSGGGGSGGGGSGGGGS)*APKDNTWYTGAKLGFSQYHDTGFINNNGPTHENQLGAGAFGGYQVNPYVGFEMGYDFLGRMPYKGSVENGAYKAQGVQLTAKLGYPITDDLDIYTRLGGMVFRADTKSNVYGKNHDTGVSPVFAGGVEYAITPEIATRLEYQFTNNIGDAHTIGTRPDNGMLSLGVSYRFGQGEAA*(GSGSHHWGSTHHHHHH)	62491.9537
**MBP_barrel _OmpA_H7**	**OmpA**	**(M)SGKIEEGKLVIWINGDKGYNGLAEVGKKFEKDTGIKVTVEHPDKLEEKFPQVAATGDGPDIIFWAHDRFGGYAQSGLLAEITPDKAFQDKLYPFTWDAVRYNGKLIAYPIAVEALSLIYNKDLLPNPPKTWEEIPALDKELKAKGKSALMFNLQEPYFTWPLIAADGGYAFKYENGKYDIKDVGVDNAGAKAGLTFLVDLIKNKHMNADTDYSIAEAAFNKGETAMTINGPWAWSNIDTSKVNYGVTVLPTFKGQPSKPFVGVLSAGINAASPNKELAKEFLENYLLTDEGLEAVNKDKPLGAVALKSYEEELAKDPRIAATMENAQKGEIMPNIPQMSA**	**83582.3794**

		**FWYAVRTAVINAASGRQTVDEALKDAQTRITKENLYFQSKIEEG*APKDNTWYTGAKLGFSQYHDTGFINNNGPTHENQLGAGAFGGYQVNPYVGFEMGYDFLGRMPYKGSVENGAYKAQGVQLTAKLGYPITDDLDIYTRLGGMVFRADTKSNVYGKNHDTGVSPVFAGGVEYAITPEIATRLEYQFTNNIGDAHTIGTRPDNGMLSLGVSYRFGQGEAA*(LLKVYGGGGSGGGGS)DVGAAAVEVEIWEDGRLLKRTRARLRVEQVDGELVTTLEFSDGTRLTTRSRMEGEIEGNTIRARFELEADGRRAVVEMEATYEQNEDGTVKLDVRIEHDGKVRELKLSFKQEHVDGKVKITIYNEDTGKTFTTTVSELELDAATGSVRVVAATEGGPEVRLEARVKPLSEV(GSGSHHWGSTHHHHHH)**	
**Barrel_OmpA_H7**	**OmpA**	***MSGAPKDNTWYTGAKLGFSQYHDTGFINNNGPTHENQLGAGAFGGYQVNPYVGFEMGYDFLGRMPYKGSVENGAYKAQGVQLTAKLGYPITDDLDIYTRLGGMVFRADTKSNVYGKNHDTGVSPVFAGGVEYAITPEIATRLEYQFTNNIGDAHTIGTRPDNGMLSLGVSYRFGQGEAA*(LLKVYGGGGSGGGGS)DVGAAAVEVEIWEDGRLLKRTRARLRVEQVDGELVTTLEFSDGTRLTTRSRMEGEIEGNTIRARFELEADGRRAVVEMEATYEQNEDGTVKLDVRIEHDGKVRELKLSFKQEHVDGKVKITIYNEDTGKTFTTTVSELELDAATGSVRVVAATEGGPEVRLEARVKPLSEV(GSGSHHWGSTHHHHHH)**	**41454.93**
GlpG_D1	GlpG	(M)ALEGLLADLELILDIFRLLALATIGAPKEELLKRFRELIKRVREAITAQLAEKGPEAEPLREAVEATLDAMERFLQAVVDGAPPEELDRLARELVERLLLTLMLAALLGSPENREIAEILLEVILKIIEAERVVRERQDPEVIEEEVEELIKELKEAAKKLDELSKDPKARGFAIAARLLVLLLLLLVLEAAGAPQEELQAAADEILQYFEDTADELVATQPAERQPLARALRRAVVALLRTALLEALGAPTEEVIAALDETVALLDEARAVAEEQLPPVQAALLSTLLVLVRTLARVYRLELEGAPREERLEVMLEALEEIQELLAEALRAEGAPEELVEQTRRVFELAIEATREMMAAGDRVVALERLAELLAAVERLLEAELRETGRLDDARAAFLAAIARQGLEFEAALAKGAPREELERLLEELLAELRAKRAELAALLRELDYQ(GGGGSGGGGSGGGGSGGGGS)*ERAGPVTWVMMIACVVVFIAMQILGDQEVMLWLAWPFDPTLKFEFWRYFTHALMHFSLMHILFNLLWWWYLGGAVEKRLGSGKLIVITLISALLSGYVQQKFSGPWFGGLSGVVYALMGYVWLRGERDPQSGIYLQRGLIIFALIWIVAGWFDLFGMSMANGAHIAGLAVGLAMAFVDSLNA*(GSGSHHWGSTHHHHHH)	73642.50
**GlpG_D4**	**GlpG**	**(M)SGRERLDRLYLEVLELRAEVLELVQEGAPAEELVRAAERLVDATLELLDLEAEEAEDLMERATLLTMRAVVLAMRLFLAVGVGAPEEEIERLVEELERVIEETEKLLEEIKGGVESLLTALALKILLSLVKSLVLTLLGAPAEEIKEFTDKQLKELLELLRETIEKAREAGAQGELFEARVLLTGLEVMKKLRETGSVLPSDELVFELVSGILRALLETAARVTGAEEEMRRLYEEAEERLRRGLAEIASLPAEEADEAFALLLAQVLRDVLVAILELVDKRAAELVRALLDFIIALAETKLEVLKEKDEEEAKKKALEGLEKVEELAKKLIELRFKDSPILENLKNAITYATELFKALVEGAELEEIEALFDKLAEYMDKAVELEVKSLPPVEAARLRHTARAGLALLRAAAAARAGRREEAEREALREFNRLVLEAARETLELERRADYQ(GGGGSGGGGSGGGGSGGGGS)*ERAGPVTWVMMIACVVVFIAMQILGDQEVMLWLAWPFDPTLKFEFWRYFTHALMHFSLMHILFNLLWWWYLGGAVEKRLGSGKLIVITLISALLSGYVQQKFSGPWFGGLSGVVYALMGYVWLRGERDPQS***	74368.9128

		GIYLQRGLIIFALIWIVAGWFDLFGMSMANGAHIAGLAVGLAMAFVDSLNA**(GSGSHHWGSTHHHHHH)**	
**GlpG_SAHA**	**GlpG_double catalytic mutant**	**(M)SGRERLDRLYLEVLELRAEVLELVQEGAPAEELVRAAERLVDATLELLDLEAEEAEDLMERATLLTMRAVVLAMRLFLAVGVGAPEEEIERLVEELERVIEETEKLLEEIKGGVESLLTALALKILLSLVKSLVLTLLGAPAEEIKEFTDKQLKELLELLRETIEKAREAGAQGELFEARVLLTGLEVMKKLRETGSVLPSDELVFELVSGILRALLETAARVTGAEEEMRRLYEEAEERLRRGLAEIASLPAEEADEAFALLLAQVLRDVLVAILELVDKRAAELVRALLDFIIALAETKLEVLKEKDEEEAKKKALEGLEKVEELAKKLIELRFKDSPILENLKNAITYATELFKALVEGAELEEIEALFDKLAEYMDKAVELEVKSLPPVEAARLRHTARAGLALLRAAAAARAGRREEAEREALREFNRLVLEAARETLELERRADYQ(GGGGSGGGGSGGGGSGGGGS)*****ERAGPVTWVMMIACVVVFIAMQILGDQEVMLWLAWPFDPTLKFEFWRYFTHALMHFSLMHILFNLLWWWYLGGAVEKRLGSGKLIVITLISALLSGYVQQKFSGPWFGGLAGVVYALMGYVWLRGERDPQSGIYLQRGLIIFALIWIVAGWFDLFGMSMANGAAIAGLAVGLAMAFVDSLNA*****(GSGSHHWGSTHHHHHH)**	74287.18
GlpG_D5	GlpG	(M)SGEERLLELQDETLLLMFEALVKVFVGAPQEELLELMKRLVELGDELMELSLETLPELEREISRIIIAYLRAVMVLILGQVEGRPREELARELQRLLDEAERALERALELADEGERLRIRLIGTLFITAIRSLLLVVEEAPEEELQKLAEELLEELRRTREEAVELAERLGDFTGTLETLLLASVTELVILATLGEPLEKLKKTYEEIVRQLDRVFDLWAAAAPEARRPEVALARDAVLLILDMMMTAEEAKASGKLVEALEKIRDDWEALLDRGAELHEGLLGEALRLLREIVRAGLTMELLKTKKAPKEEVIKKAKELLKRLLEAAERYLKAAGVPEEKRKKYMEYLKRVAELALEMLETEDEEKKKEARLKLLEIMVEVLEENVEEAPPEERPELLLLATWIKAGYKLEKAEIDGASQEEKKKIAEEVLKEIRETLERVRELQRERDYQ(GGGGSGGGGSGGGGSGGGGS)*ERAGPVTWVMMIACVVVFIAMQILGDQEVMLWLAWPFDPTLKFEFWRYFTHALMHFSLMHILFNLLWWWYLGGAVEKRLGSGKLIVITLISALLSGYVQQKFSGPWFGGLSGVVYALMGYVWLRGERDPQSGIYLQRGLIIFALIWIVAGWFDLFGMSMANGAHIAGLAVGLAMAFVDSLNA*(GSGSHHWGSTHHHHHH)	75733.8395
**MspA_SBDs**	**MspA_SBDs**	**(M)SGTNEQNIQAVMDFFDLVGEIRRRRAERLREEIERLREREKEDGELARGIILLGEAMYYAGSHGPVEGLYYTLKKLQEAQKVLTE*****WSLGVGINFSYTTPNILIDDGDITRPPFGLNSVITPNLFPGVSISADLGN*****(GSGSHHWGSTHHHHHH)**	**17091.13**
N-D7	miniCXCR4	(M)GGSLLEETEKLAEELGKKEELKKLKEKLKKLYEEAKKAKEPAKVLDIYAEAVRLIAKEYIKLKYEKIEKDESVSDFVKQLAKTQLEALKKLEEEKDEEKKNLLIKLMTNASALLKLKERVKELSKRRPEVTLAVELLDKAEKLFEEALKEKDEKKKTELLNKALEYLTLAELYLELLEALEAYEGDPLYDEMLSYVKKIVELLVKYYEEKLDEDEVLKEVNELMLKLQILEARVLARRAADDPERQAKYEKILEYLEELLKATDEEEKNRLLLEAMMLLAELELEEWIEKWREKYKDDEEILKLLDEIEELMEKAEEAEDEDELTKILKQANFLASEAVLRISLREYEEILKDDEEAYKEAKELIKELKEALKKGDGEKALVNALKIQLLVLYL(GSGGGSGGGS)*SMKEPCFREENANFNKIFLPTIYGQKGRPFWAVDAVANWYFGNFLCKAVHVIYTVNLYRGRPGALLLTIPDFIFANVSEADDRYICDRFYPNDLWVV*	66468.3465

		VFQFQHIMVLSDRGWLPYYIGISIDSFILLEIIKQGCEFENTVHKWISITEAL(G SGSHHWGSTHHHHHH)	
**C-D7**	**miniCXCR4**	**(M)*SMKEPCFREENANFNKIFLPTIYGQKGRPFWAVDAVANWYFGNFLCKAVHVIYTVNLYRGRPGALLLTIPDFIFANVSEADDRYICDRFYPNDLWVVVVFQFQHIMVLSDRGWLPYYIGISIDSFILLEIIKQGCEFENTVHKWISITEAL*(GGGSGGGSGS)LLEETEKLAEELGKKEELKKLKEKLKKLYEEAKKAKEPAKVLDIYAEAVRLIAKEYIKLKYEKIEKDESVSDFVKQLAKTQLEALKKLEEEKDEEKKNLLIKLMTNASALLKLKERVKELSKRRPEVTLAVELLDKAEKLFEEALKEKDEKKKTELLNKALEYLTLAELYLELLEALEAYEGDPLYDEMLSYVKKIVELLVKYYEEKLDEDEVLKEVNELMLKLQILEARVLARRAADDPERQAKYEKILEYLEELLKATDEEEKNRLLLEAMMLLAELELEEWIEKWREKYKDDEEILKLLDEIEELMEKAEEAEDEDELTKILKQANFLASEAVLRISLREYEEILKDDEEAYKEAKELIKELKEALKKGDGEKALVNALKIQLLVLYL(GSGSHHWGSTHHHHHH)**	66267.1647
G8	miniCXCR4	(M)GGMVDEALTLLGRAIMLMSAQPEEEQKKYREQLQPLARRLAELIKEKLEEAMKEDIEKAFEWAQKLAQLLTLGDLLGDTEWVKKEVEELEPKLRELVKKQIELLKAQAEELKDDAIAVARNLAVALAMAKAALPKEEYEKVAKEIKERFEEILDELIKALKERAEEILDEGFSAFVELFQDILDFMGAAKLLGDEEGLKKLQEAAKELLPIYEEAAERYREEHAGSALAELEAENFRTMATIILEDEESAEKAIEELKEFIKENLEAVVEELEEMRERGELSLEEYAVLTALLLLMAKYVGKEELAEKLKEKLKEITKELADKLVREAEELKGKDPDRLALILTQLLQLILLLRALGEDELADELLDFVLRLSREMLEPFLEALQRKVEELERE(GSGGGSGGGS)*SMKEPCFREENANFNKIFLPTIYGQKGRPFWAVDAVANWYFGNFLCKAVHVIYTVNLYRGRPGALLLTIPDFIFANVSEADDRYICDRFYPNDLWVVVVFQFQHIMVLSDRGWLPYYIGISIDSFILLEIIKQGCEFENTVHKWISITEAL*(GSGSHHWGSTHHHHHH)	65348.8493
HeR_E2	Heliorhodopsin	(M)SGNIEEFKAMMDLAIEILELFAQVALGAPKEEIRAEADELIESLIKLTNIVTSTEDETAKLLHNALGLTLILGFEVAVASIIGAPKEEVIKKIKKYLKEIEENVKKAAEKLDGLDKSLLLFIFEFLRLIVETGLLILEGKSKEEVIKTLKDEVKDMIDAILKAGEEEDAANAEFYKHMAKAFEHLMEAAVAAISGEPVEKVVEHLRKAIEHVEKAIDIAVKQYNEEQAEAMRGMGKALSYALEMLAASVAGAPLEEQKKLFEEFLKALEEMLDAIAKMLDEENSALVKMLSEILLFFFEATYLVFLNAPKEKVDELLEKHTEKAQKYLRELFKLQGKPEEEIELGVKLLTVSLKTLRLLLTQAPKEEIYEQFKEYLEVIIEAFEARAEEEDEETKELLELLKEMLELALKLLEALYKGAKKEEIRKIIKEIIKKIKEAVEKLAEMMKKEDYQ(GGGGSGGGGSGGGGSGGGGS)*TENEEINFRKFRIFNGIMGVIHLIQVFLVLYLSNNFSLPITVNKPVYNEITNSISPVAETLFSIEIGPLVAMFLFISATAHILIATVLYYRYVQNLKNHMNPYRWFEYSISASFMIVIIAMLTTIYDLGTLLALFTLTAVMNLMGLMMELHNQTTQNTNWTSYIIGCIAGFVPWIVIFIPLISAESVPDFVIYIFISIAIFFNCFAINMYLQYKKIGKWKNYLHGEKVYIILSLVAKSALAWQVFAGTLRPM*(GSGSHHWGSTHHHHHH)	83428.2397

HeR_E3	Heliorhodopsin	(M)SGNEEEDELFLKAILNFIKAQQLAAAGAPRERVEELTEKAIEAINKIAEIESKSLDADDAAIVTAYYKIIALAVRLAVIQLAGGDDEEIIEAIKELLKALEDFEKAVTKTEDAKNAAIAKLIVLFGKLFLETGILILKGEPKEKIVKKFKEYVKEIVKALKDLAKELDAGEAKAVNLAAEVLENVVKAAVLIIEGGPKEEILKQIENAQKNLDALTKEVAAIYPADLGKLATALLEAFRNALQAALLGLIGADRDELADLLRKAFDALRDFYVALLELGSEALAPLAKEVRETLTAARDRFLALFDGGTKEDFAEGLALLVRAVYLLLIGFLVEDGAPEEEIEEMREIADLVAKVVRLLVLGAPLEEVEPYIVSLTEKLLNKFLEKAKEISEEEAKVAEVMIRAVKESYRLVKETAPSGDQEKILELMEKLNELIDELVNTAIETIKELDYQ(GGGGSGGGGSGGGGSGGGGS)*TENEEINFRKFRIFNGIMGVIHLIQVFLVLYLSNNFSLPITVNKPVYNEITNSISPVAETLFSIEIGPLVAMFLFISATAHILIATVLYYRYVQNLKNHMNPYRWFEYSISASFMIVIIAMLTTIYDLGTLLALFTLTAVMNLMGLMMELHNQTTQNTNWTSYIIGCIAGFVPWIVIFIPLISAESVPDFVIYIFISIAIFFNCFAINMYLQYKKIGKWKNYLHGEKVYIILSLVAKSALAWQVFAGTLRPM*(GSGSHHWGSTHHHHHH)	81786.8227
HeR_E5	Heliorhodopsin	(M)SGNIEEEKYMLEAIIEIAKLFFDVALGAPKEVIAKHLDALIDAITKYYDEMAKEEDEAGKLAIEILRLSMLLGLKVVKFALTGAPVEELEKEIEKILKEIEENLKKYEKLTDPKDAALLKFIVYFMHMITKTGLAILKGKSQEEILKIIEEESKKAIEALEKLAEELDPEQGEITREAARAFRLIVEMGLAMIKGEPEEKIRKLLKELEEELDRLRELILKTEPADQGAMMAEMVEALSLAIRMAVESLLGAPQELHDKLFEQFLARLQALRDIVEKTLPPEQAAEVSLVLTAVIDVIKIVKAFFSGAPKEELAKLFEEHARTLQEGTAELLRTLGEPEEEIKRAEELIEASVHMALLIALNAPKEEILEAGEKFLELVKKAMEEIVKKLGEEIKEEVELINKIMEESLELAKLIAEGAPKEEIRKKAEEILKLLKELAEKIIKWLKEKDYQ(GGGGSGGGGSGGGGSGGGGS)*TENEEINFRKFRIFNGIMGVIHLIQVFLVLYLSNNFSLPITVNKPVYNEITNSISPVAETLFSIEIGPLVAMFLFISATAHILIATVLYYRYVQNLKNHMNPYRWFEYSISASFMIVIIAMLTTIYDLGTLLALFTLTAVMNLMGLMMELHNQTTQNTNWTSYIIGCIAGFVPWIVIFIPLISAESVPDFVIYIFISIAIFFNCFAINMYLQYKKIGKWKNYLHGEKVYIILSLVAKSALAWQVFAGTLRPM*(GSGSHHWGSTHHHHHH)	83332.3397
MBP_barrel_HeR_G12	Heliorhodopsin	(M)SGKIEEGKLVIWINGDKGYNGLAEVGKKFEKDTGIKVTVEHPDKLEEKFPQVAATGDGPDIIFWAHDRFGGYAQSGLLAEITPDKAFQDKLYPFTWDAVRYNGKLIAYPIAVEALSLIYNKDLLPNPPKTWEEIPALDKELKAKGKSALMFNLQEPYFTWPLIAADGGYAFKYENGKYDIKDVGVDNAGAKAGLTFLVDLIKNKHMNADTDYSIAEAAFNKGETAMTINGPWAWSNIDTSKVNYGVTVLPTFKGQPSKPFVGVLSAGINAASPNKELAKEFLENYLLTDEGLEAVNKDKPLGAVALKSYEEELAKDPRIAATMENAQKGEIMPNIPQMSAFWYAVRTAVINAASGRQTVDEALKDAQTRITKENLYFQSKIEEG*TENEEINFRKFRIFNGIMGVIHLIQVFLVLYLSNNFSLPITVNKPVYNEITNSISPVAETLFSIEIGPLVAMFLFISATAHILIATVLYYRYVQNLKNHMNPYRWFEYSISASFMIVIIAMLTTIYDLGTLLALFTLTAVMNLMGLMMELHNQTTQNTNWTSYIIGCIAGFVPWIVIFIPLISAESVPDFVIYIFISIAIFFNCFAINMYLQYKKIGKWKNYLHGEKVYIILSLVAKSALAWQVFAGTLRPMVY*(GGGGSGGGGS)AKTYKMKMKGKLESEDITLEFEAEAKVTITPDEVKVEIKVKVKDVDSGIEGKGELKVEVKLGKEIKQKIELKIEIEDETLSAKYESELKKEDGKWIHKSK	111962.7942

		LEIKDKEDETVLEIKAESEISKDGKLKQKIEAKEVESGSKLKIKTEVKVVDPKTAKFKKEIELEDKSGKKDSIKIEGEVKVETGKIKVKAEVELTSGKKIKIELEAETEYDEEKGTVKSKVKIKSSTGLKVEAEMELNVDDLKKGAFKFKLKIKSSDGLKIELETYISVDKEKLEIKLKSKFKSTNKEFTFEVESELKIDKEKGEIEVKIKATDYEGNVKEAKMKIKMEFEKE(GSGSHHWGSTHHHHHH)	
Aqp_A1	Aquaporin M	(M)SGELEEAFKLITQLARDFFKTAAEIFREQGNELGAKIMEVYAKYFEEMIKNPEEAPRLFLKMHADVARVIAEFARKRGDLAAAKMAEAAAKFFELLAENPEEFQEAFLKGAALLMRVGAEEAREAGDEALAEALERAAELFEEALKDPSKAGEYLVEAMRVLAEALAELAEERGDLATARALRTLVETLKEIEKNKDNLHLEVERLTLRLMLVNLRAAAEDGWTEFLRVADILEKAIEALYGSEEDRKRYTLEVAAELARVVSRYARRVGDELGARLFEVVAERLEKALETLDKIEEEMSKALVDMLRAAAEWVRTQGNEDAAEILDLLAKIMESIINNPENFAKEAKEYLEKIKDLSIKSLEKRGDTDGVKLFSVLSDSFIKLLEFEIEKDYQGDRS(GGGGSGGGGS)*VSLTKRCIAEFIGTFILVFFGAGSAAVTLMIASGGTSPNPFNIGIGLLGGLGDWVAIGLAFGFAIAASIYALGNISGCHINPAVTIGLWSVKKFPGREVVPYIIAQLLGAAFGSFIFLQCAGIGAATVGGLGATAPFPGISYWQAMLAEVVGTFLLMITIMGIAVDERAPKGFAGIIIGLTVAGIITTLGNISGSSLNPARTFGPYLNDMIFAGTDLWNYYSIYVIGPIVGAVLAALTYQYLTSEGSRDRS*(GSGSHHWGSTHHHHHH)	73384.8977
Aqp_A2	Aquaporin M	(M)SGSHEEAMKVILKNAKKLFDTYAEIAKEQGNMDGAALMEAIAKSLEEAMKNPEEGVQMLFKLYADIMKVLAEINKKKGDKLGYIMNEKAAELFERLAENPEDFGKVLLEGFRDLAKAMAKYYKEKGDKENAEAAEKVAELLDKAVKDTENLGEYILDLIITMLEYLAEKAEELGDTATAEVLRVAAEQLKEILENPENLFEEILKTFAGMYNVQAEHLRKEGDEEGAAAMETIAELFNVLAENPENAREEVIELLRRLREEMRAYMEKIGRTVMGRMLEVLLDHLAEALEDEENAQREALLGFADVFRVLAEAMREQGNEDGAEIADALAKTFEEAAKKLEDMVKVLLKGLKEVLELIAEQAKEQGNETAAKLAETFAEILEEMLEALEKEDYQGDRS(GGGGSGGGGS)*VSLTKRCIAEFIGTFILVFFGAGSAAVTLMIASGGTSPNPFNIGIGLLGGLGDWVAIGLAFGFAIAASIYALGNISGCHINPAVTIGLWSVKKFPGREVVPYIIAQLLGAAFGSFIFLQCAGIGAATVGGLGATAPFPGISYWQAMLAEVVGTFLLMITIMGIAVDERAPKGFAGIIIGLTVAGIITTLGNISGSSLNPARTFGPYLNDMIFAGTDLWNYYSIYVIGPIVGAVLAALTYQYLTSEGSRDRS*(GSGSHHWGSTHHHHHH)	73164.5984
Aqp_A3	Aquaporin M	(M)SGELEETTRLIAEMQARLYQTMADILKAQGNKFGAELMQLAADGYKEMAENVEEALKVFFKLQADLAKLIAKHNKEKGDKEAYELNELQAKLFEKLAENPEKIQEVVLETFLEIMKLMAEYYKEKGEMELAELYEKIAEKLEEALENKETAGEHLLEAFILLLEALAKEAEKVGMKEEAEILRAAADTLREDYEAGKDPRTMSINVMATMFEVAAEHLKKEGETDYAAVMEQIAAMFKAYAEDPENAKPKVIEQLKKLAEHLAKAAETSANSLTLKVNKVLAEYLVKAMEDEKNMQEIALLGLAEAFEVIAEEYLAQGNEDGAEVALKLAEIFRILAKDPVENIEDVLKGLRDLMKEVAEQARKAGDEKAAKLADTFAKILEDWLKHLKEQ((GGGGSGGGGS)*VSLTKRCIAEFIGTFILVFFGAGSAAVTLMIASGGTSPNPFNIGIGLLGGLGDWVAIGLAFGFAIAASIYALGNISGCHINPAVTIGLWSVKKFPGREVVPYIIAQLLGAAFGSFIFLQCAGIGAATVGGLGATAPFPGISY*	77476.11

		WQAMLAEVVGTFLLMITIMGIAVDERAPKGFAGIIIGLTVAGIITTLGNISGSSLNPARTFGPYLNDMIFAGTDLWNYYSIYVIGPIVGAVLAALTYQYLTSEGSRDRSMKEVAEQARKAGDEKAAKLADTFAKILEDWLKHLKEQDYQGDRS(GSGSHHWGSTHHHHHH)	

Table S3. Cryo-EM data collection, processing, and analysis statistics for OmpA, GlpG, and TP proteins.

Data Collection	OmpA Helical WRAP	OmpA Barrel WRAP	WRAP-GlpG	TP0126-WRAP	WRAP-TP0698
**Microscope**	Titan Krios	Titan Krios	Titan Krios	Titan Krios	Titan Krios
**Voltage (kV)**	300	300	300	300	300
**Detector**	Gatan K3	Gatan K3	Gatan K3	Gatan K3	Gatan K3
**Energy filter**	Gatan BioQuantum Gif	Gatan BioQuantum Gif	Gatan BioQuantum Gif	Gatan BioQuantum Gif	Gatan BioQuantum Gif
**Recording mode**	Counting	Counting	Counting	SuperResolution	SuperResolution
**Magnification (kX)**	105	105	105,000 X	105,000 X	105,000 X
**Movie micrograph pixel size (Å)**	0.83	0.83	0.83	0.42→0.84 (½ F-crop)	0.42→0.84 (½ F-crop)
**Dose rate (e−/Å2/s)**	10	10	10	10	10
**No. of frames per movie micrograph**	99	99	99	99	99
**Frame exposure time (s)**	0.0505	0.0505	0.0505	0.0505	0.0505
**Movie micrograph exposure time (s)**	5.0	5.0	5.0	5.0	5.0
**Total dose (e−/Å2)**	50	50	50	50	50
**Under focus range (μm)**	0.7-1.7	0.8-1.8	0.8 - 1.8	0.8 - 1.8	0.8 - 1.8
**Number of movie micrographs**	1948	1512	4,074	4,368	4,233
**Tilt Angles (°)**	0	0	0	0	0
**Map Processing**					
**Symmetry imposed**	C1	C1	C1	NA	C1
**Extraction box size (pix)**	220	256	288	N/A	476
**Initial particle images (no.)**	277,641	3,232,065	1,115,682	N/A	131,770

Data Collection	OmpA Helical WRAP	OmpA Barrel WRAP	WRAP-GlpG	TP0126-WRAP	WRAP-TP0698
**Microscope**	Titan Krios	Titan Krios	Titan Krios	Titan Krios	Titan Krios
**Final particle images (no.)**	133,256	117,392	43,571	N/A	47,941
**Map resolution (Å)**	4.88	5.69	4.74	N/A	4.01
**FCS threshold**	0.143	0.143	0.143	N/A	0.143
**EMDB ID**	EMD-75291	EMD-75290	EMD-75346	N/A	EMD-75350

Table S4. Cryo-EM data collection, processing, and analysis statistics for WRAP-MspA_SBD

**Data Collection**	**WRAP-MspA_SBD**	
Microscope	Titan Krios	
Voltage (kV)	300	
Detector	Gatan K3	
Energy filter	Gatan BioQuantum Gif	
Recording mode	Counting	
Magnification (kX)	105,000 X	
Movie micrograph pixel size (Å)	0.83	
Dose rate ($e^{-}/Å^{2}/s$)	10	
No. of frames per movie micrograph	99	
Frame exposure time (s)	0.0505	
Movie micrograph exposure time (s)	5.0	
Total dose ($e^{-}/Å^{2}$)	50	
Under focus range (µm)	0.8 - 1.8	
Number of movie micrographs	13,653	
Tilt Angles (°)	0, 15, 25, 30	
**Map Processing**		
Symmetry imposed	D8	C1
Extraction box size (pix)	350	350
Initial particle images (no.)	6,748,083	6,748,083
Final particle images (no.)	64,941	64,941
Map resolution (Å)	2.95	2.95
FCS threshold	0.143	0.143
Map resolution range (Å)	2.55 - 3.13	2.92 - 3.81
**Refinement**		
PDB ID	9ORP	9OV5
EMDB ID	EMD-70780	EMD-70890
Initial model used	Computationally designed model	Computationally designed model
Map resolution (Å)	2.95	3.34
FCS threshold	0.143	0.143
Model resolution range (Å)	2.55 - 3.13	2.92 - 3.81
Map sharpening B factor	138.1	90.2
Model composition		
Non-hydrogen atoms	16,368	15,580
Protein Residues	2,144	2,089
Ligands	—	—
B factors (Å)		
Protein	36.80	58.46
Ligands	—	—
R.M.S. deviations		

Data Collection	WRAP-MspA_SBD	
Microscope	Titan Krios	
Bond lengths (Å)	0.003	0.003
Bond angles (°)	0.676	0.595
Validation		
MolProbity score	1.18	1.34
Clashscore	3.94	4.35
Rotamer outliers (%)	0.38	1.47
Ramachandran plot		
Favored (%)	100.00	98.37
Allowed (%)	0.00	1.63
Disallowed (%)	0.00	0.00

Table S5. Summary of Steady-state binding affinities (K_D) and kinetic parameters of miniCXCR4-WRAP to CXCL12 following pre-incubation under varying conditions.

Pre-treatment condition	Steady state K_D (nM)	Steady state K_D Error (nM)	Kinetic K_D (nM)	Kinetic K_D Error (nM)	k_{on} ($M^{-1}s^{-1}$)	K_{on} Error ($M^{-1}s^{-1}$)	k_{off} (s^{-1})	k_{off} Error (s^{-1})
**25°C (no pre-treatment)**	390.00	90.00	31.90	0.38	8.28E+03	4.49E+01	2.64E-04	2.84E-06
**55°C, 15 min → 25°C, 1 h**	230.00	84.00	18.20	0.23	1.49E+04	8.48E+01	2.72E-04	3.00E-06
**95°C, 15 min → 25 °C, 1 h**	53.00	20.00	9.71	0.11	3.46E+04	1.96E+02	3.36E-04	3.24E-06
**55°C, 15 min → 25°C, 12 h**	290.00	51.00	20.10	0.29	1.71E+04	1.31E+02	3.44E-04	4.15E-06
**95°C, 15 min → 25°C, 12 h**	67.00	15.00	8.24	0.08	3.84E+04	1.90E+02	3.16E-04	2.82E-06
**25°C, 1 week**	530.00	110.00	24.60	0.29	4.41E+04	4.40E+02	1.09E-03	6.78E-06
**Concentrated 25°C**	280.00	36.00	20.30	0.25	3.46E+04	3.32E+02	7.04E-04	5.50E-06

Proteins were pre-incubated under the indicated conditions prior to binding measurements. Thermal treatments were performed at 55°C or 95°C for 15 minutes (min), followed by equilibration at 25°C for 1 hour (h) or 12 hours. Long-term stability conditions correspond to incubation at 25 for 1 week. All binding measurements were performed at 25°C using bio-layer interferometry (BLI). Because kinetic K_D values were not fully concordant with steady-state estimates, steady-state K_D values are used as the primary reported measure of binding affinity.
